# HIF1 activation safeguards cortical bone formation against impaired oxidative phosphorylation

**DOI:** 10.1172/jci.insight.182330

**Published:** 2024-08-01

**Authors:** Mohd P. Khan, Elena Sabini, Katherine Beigel, Giulia Lanzolla, Brittany Laslow, Dian Wang, Christophe Merceron, Amato Giaccia, Fanxin Long, Deanne Taylor, Ernestina Schipani

**Affiliations:** 1 Department of Orthopaedic Surgery, University of Pennsylvania, Perelman School of Medicine, Philadelphia, Pennsylvania, USA.; 2 Department of Orthopaedic Surgery, School of Medicine, University of Michigan, Ann Arbor, Michigan, USA.; 3 Department of Biomedical and Health Informatics, The Children’s Hospital of Philadelphia, Philadelphia, Pennsylvania, USA.; 4 Department of Oncology, University of Oxford, Oxford, United Kingdom.; 5 Department of Orthopaedic Surgery, The Children’s Hospital of Philadelphia, Philadelphia, Pennsylvania, USA.

**Keywords:** Bone biology, Bone development, Hypoxia, Mitochondria

## Abstract

Energy metabolism, through pathways such as oxidative phosphorylation (OxPhos) and glycolysis, plays a pivotal role in cellular differentiation and function. Our study investigates the impact of OxPhos disruption in cortical bone development by deleting mitochondrial transcription factor A (TFAM). TFAM controls OxPhos by regulating the transcription of mitochondrial genes. The cortical bone, constituting the long bones’ rigid shell, is sheathed by the periosteum, a connective tissue layer populated with skeletal progenitors that spawn osteoblasts, the bone-forming cells. TFAM-deficient mice presented with thinner cortical bone, spontaneous midshaft fractures, and compromised periosteal cell bioenergetics, characterized by reduced ATP levels. Additionally, they exhibited an enlarged periosteal progenitor cell pool with impaired osteoblast differentiation. Increasing hypoxia-inducible factor 1a (HIF1) activity within periosteal cells substantially mitigated the detrimental effects induced by TFAM deletion. HIF1 is known to promote glycolysis in all cell types. Our findings underscore the indispensability of OxPhos for the proper accrual of cortical bone mass and indicate a compensatory mechanism between OxPhos and glycolysis in periosteal cells. The study opens new avenues for understanding the relationship between energy metabolism and skeletal health and suggests that modulating bioenergetic pathways may provide a therapeutic avenue for conditions characterized by bone fragility.

## Abstract 2

Our study reveals that interfering with cellular energy processes causes thin, fragile bones. Enhancing a protein that supports energy under low oxygen improves bone strength, offering treatment insight for fragile bones.

## Introduction

Cells primarily utilize 2 methods for energy production: glycolysis coupled with lactic acid fermentation in the cytosol and the tricarboxylic acid (TCA) cycle/oxidative phosphorylation (OxPhos) in mitochondria. The choice between these energy sources during cellular differentiation can influence cell fate (1, 2) through a variety of modalities, including the production of adenosine triphosphate (ATP) (1–3).

Skeletal development depends on 2 mechanisms, intramembranous and endochondral. Flat skull bones arise through intramembranous ossification, whereas the remainder of the skeleton, including the long bones, is sculpted through the endochondral route (4–7). In the long bones, the endochondral pathway is initiated with mesenchymal cells in the limb bud differentiating into chondrocytes to form a cartilage anlage, the fetal growth plate, surrounded by a thin layer of fibroblast-like cells, the perichondrium. Perichondrial cells differentiate into osteoblasts, i.e., the bone-forming cells, giving origin to the “bone collar.” The cartilaginous anlage is eventually invaded by blood vessels originating from the bone collar, and this leads to an influx of skeletal progenitors, osteoblasts, and osteoclasts that results in the formation of trabecular bone. Osteoclasts originate from hematopoietic cells and are bone-resorbing cells.

The bone collar not only serves as the source for cortical bone, the rigid outer shell of long bones, but also plays a pivotal role in skeletal development. The outer surface of cortical bone is enveloped by a dense layer of connective tissue known as the periosteum. The periosteum is a thin yet critical tissue that contributes to bone development, postnatal appositional bone growth, adaptation of bone in response to mechanical loads, and repair of fractures. The periosteum is highly enriched in skeletal progenitors, which have the capacity to differentiate into osteoblasts. Contrary to the trabecular bone and the endosteal surface of cortical bone, the periosteum contains a negligible number of osteoclasts. On the inner surface, the cortical bone is lined by a thin layer of connective tissue known as endosteum, which is also rich in osteoblasts originating from skeletal progenitors present in the bone marrow. During the development of cortical bone, primarily postnatally, the mineralized bone matrix is deposited by periosteal osteoblasts on the outer surface and concurrently by endosteal osteoblasts on the inner surface. Meanwhile, the remodeling of bone is underway as the old bone matrix is being resorbed by endosteal osteoclasts. The proper balance between bone formation and bone resorption determines the final size and shape of cortical bone.

In vitro studies showed both glycolysis and OxPhos are relevant during osteoblast differentiation (2, 8). In vivo, factors like hypoxia-inducible factor 1a (HIF1) increase osteoblast activity and bone mass by promoting glycolysis (9). Conversely, the role of OxPhos in the control of bone mass in vivo remains understudied.

In this study, we generated mutant mice lacking mitochondrial transcription factor A (TFAM), a key regulator of OxPhos (10), in mesenchymal progenitors of the limb bud that give origin to the long bones. TFAM regulates the transcription of mitochondrial genes that encode 13 subunits of the electron transport chain and thus controls OxPhos. The TFAM impairment of OxPhos is the most powerful, most consistent, and best characterized biological consequence of loss of TFAM across a large variety of tissues and cell types, including growth plate chondrocytes (4, 11).

Corroborating recent findings (12), loss of osteoblastic TFAM resulted in a severe bone phenotype with the most dramatic feature being spontaneous fracture of the midshaft of long bones. The weakened structural integrity of long bones stemmed from substantial cortical bone thinning, mainly attributable to diminished periosteal bone formation. Associated with this was a decrease in ATP levels within periosteal cells and a skewed balance between skeletal progenitors and mature osteoblasts in the periosteum. Remarkably, augmenting osteoblastic HIF1 activity proved efficacious in substantially correcting the dramatic bone phenotype and restoring ATP concentrations in periosteal cells compromised by TFAM loss.

## Results

### Low cortical bone mass and spontaneous fractures are observed in mutant mice lacking TFAM in PRX1-Cre lineage cells.

We conditionally knocked out TFAM in mesenchymal progenitors of the limb bud and their descendants using a *PRX1*-Cre driver (13) (PRX) and a mouse homozygous for a floxed TFAM allele (*TFAM^fl/fl^*) (10, 14). These progenitors give rise to chondrocytes and osteoblasts in the long bones of the limbs, sterna, and parietal bones of the skull (13). Mutant mice, denoted as *PRX TFAM^fl/fl^* (TFAM), were born at the expected Mendelian frequency. Both male and female mutant mice, along with control (CTRL) littermates, were analyzed, with at least 5 mice in each group. A previous report from our laboratory indicated that although impairing mitochondrial respiration through the loss of TFAM preserves the overall structure of the developing growth plate, it significantly delays chondrocyte terminal differentiation and the replacement of the cartilage anlage by bone (4). Consistent with these previous findings, at birth, *PRX TFAM^fl/+^* (Het-TFAM) and *TFAM^fl/fl^* (CTRL) mice appeared phenotypically similar, but newborn TFAM mutant animals exhibited shorter limbs (data not shown).

**Figure 1:**
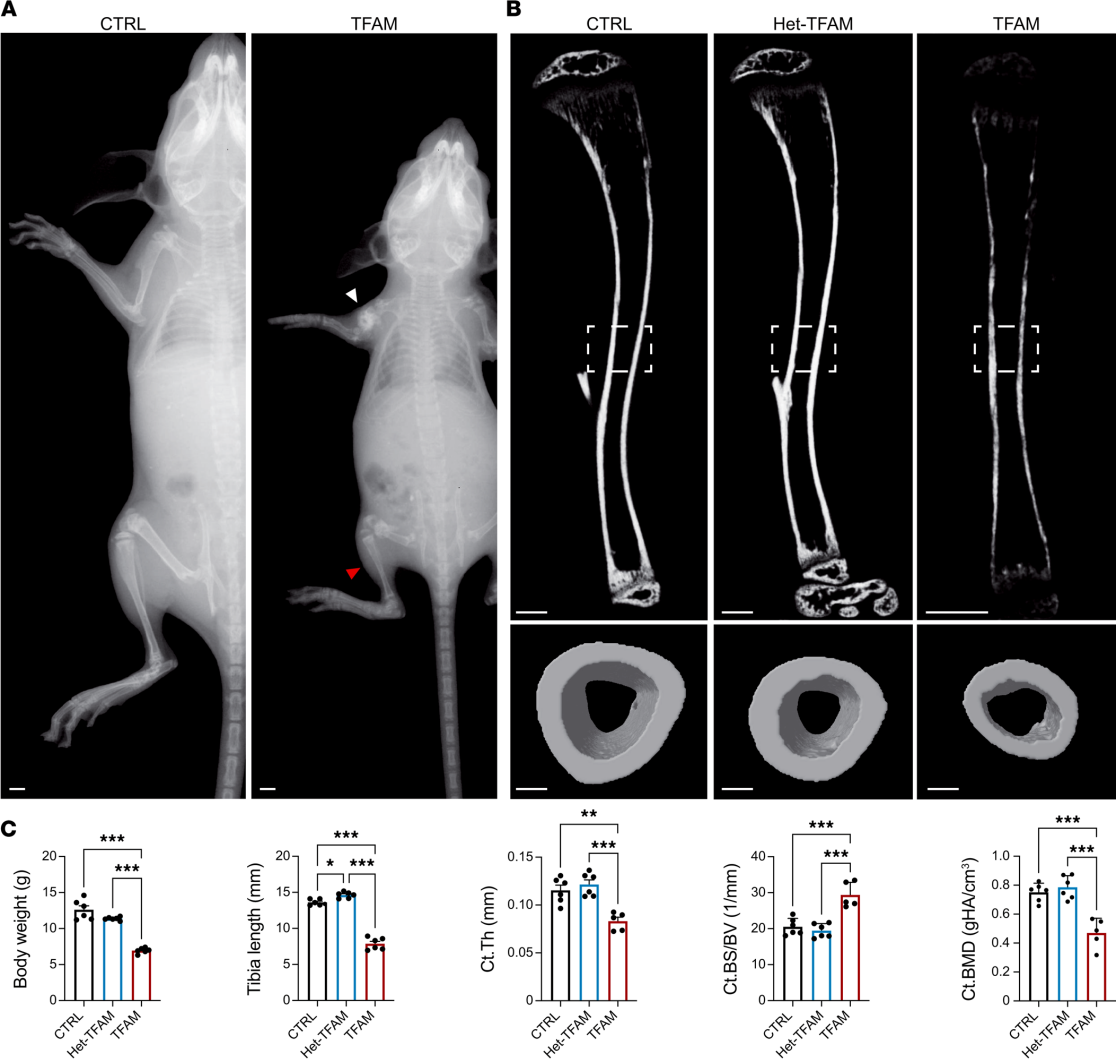
Loss of TFAM in PRX lineage cells results in low cortical bone mass and spontaneous fractures. (**A**) X-ray images of postnatal day 21 (p21) *TFAM^fl/fl^* (CTRL) and *PRX TFAM^fl/fl^* (TFAM) mutant female mice. The white arrow indicates multiple midshaft fractures in the forelimb of mutant mice, while the hind limb of the same mutant mouse shows no evidence of fractures (red arrow). Scale bars: 1 mm. (**B**) Longitudinal (top) and midshaft transverse (bottom) micro-CT scans of tibias isolated from CTRL, *PRX TFAM^fl/+^* (Het-TFAM), and TFAM p21 female mice. Scale bars: 500 μm for the longitudinal section and 100 μm for the transverse section. (**C**) Analysis of morphometric and micro-CT parameters, including body weight, tibia length, cortical thickness (Ct.Th), ratio of cortical bone surface to bone volume (Ct.BS/BV), and cortical bone mineral density (Ct.BMD). Evaluations were conducted on mutant mice and their respective CTRL littermates, with a minimum of 5 mice per group. gHA, grams hydroxyapatite. Statistical analysis employed 1-way ANOVA complemented with Bonferroni’s post hoc test for multiple comparisons. Data are presented as mean ± standard deviation (SD). Significance levels are denoted as *P ≤ 0.05, **P ≤ 0.01, ***P ≤ 0.001.

X-ray analysis at postnatal day 21 (p21) revealed multiple midshaft fractures in the long bones of both forelimbs and hind limbs of mutant mice, which began occurring independent of sex as early as 1 week after birth (Figure 1 and Supplemental Figure 1A; supplemental material available online with this article; https://doi.org/10.1172/jci.insight.182330DS1). Due to these multiple fractures that significantly hindered their mobility, TFAM mutant mice were euthanized by p21. These circumstances prevented a systematic investigation into whether the mutation affected the healing process of the fracture callus.

Notably, at p21, mutant mice were overall smaller, were lighter, and had shorter long bones than controls (Figure 1, A and C, and Supplemental Figure 1, A and C). This shortening of long bones verified that the loss of TFAM in PRX lineage cells delays endochondral bone development, and this delay persists postnatally.

As the number of fractures was significantly lower in hind limbs, consistent with higher transgene activity in the forelimbs of PRX mice (13), we focused our subsequent analysis exclusively on p21 mutant tibias without overt fractures. Additionally, since the presence of midshaft fractures indicates a severe cortical bone defect, we mainly studied cortical bone.

**Figure 2:**
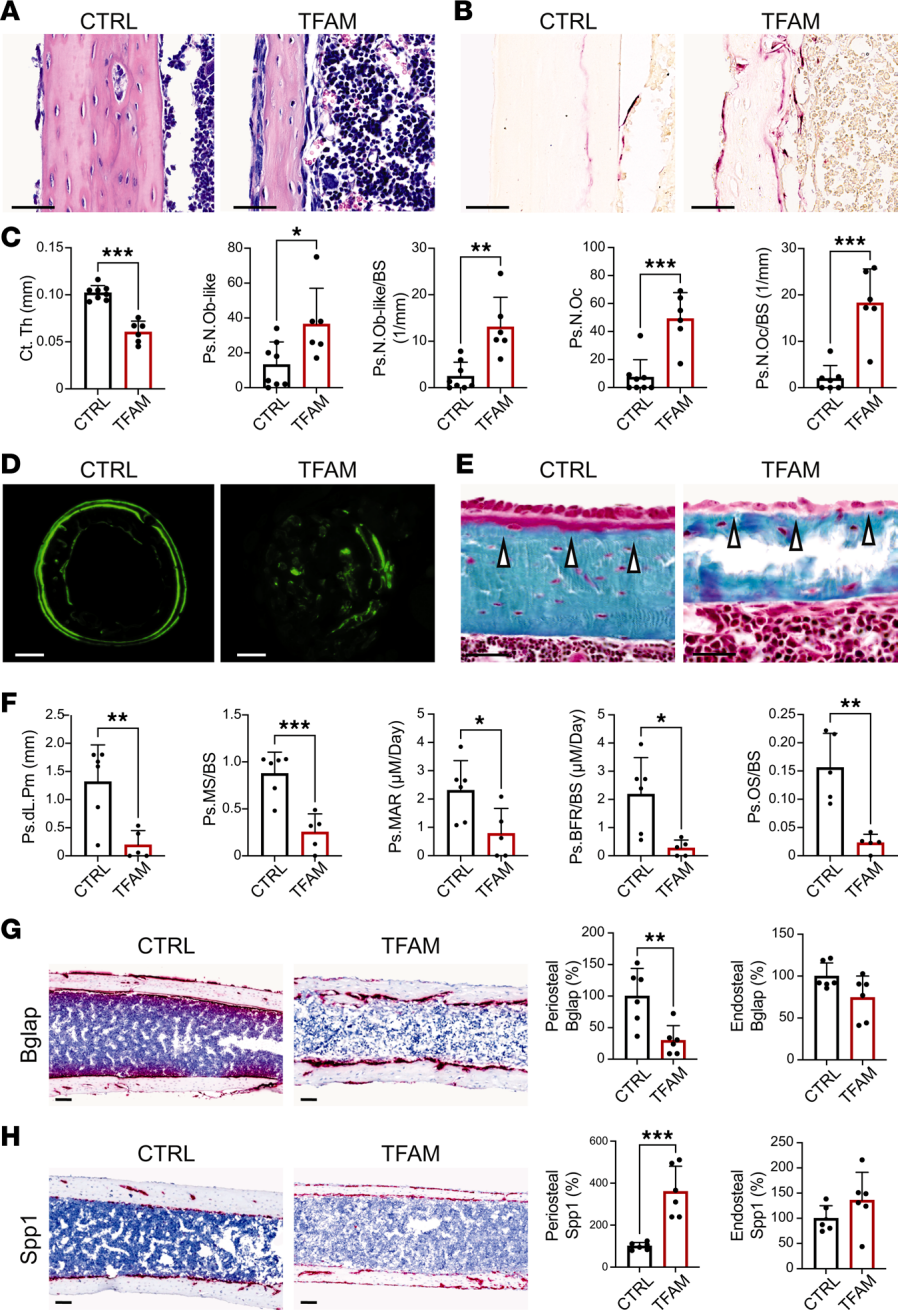
Loss of TFAM enhances cellularity but impairs osteoblast activity in the periosteum. (**A**) H&E staining of longitudinal paraffin sections of p21 tibias, illustrating cortical bone at midshaft. Scale bar: 100 μm. (**B**) Tartrate-resistant acid phosphatase (TRAP) staining of longitudinal paraffin sections of p21 tibias, visualizing osteoclasts in cortical bone. Scale bar: 100 μm. (**C**) Static histomorphometry analysis conducted on longitudinal paraffin sections of p21 tibias, quantifying cortical thickness (Ct.Th), number of periosteal osteoblast-like cells (Ps.N.Ob-like), osteoblast-like cells per bone surface (Ps.N.Ob-like/BS), number of periosteal osteoclasts (Ps.N.Oc), and osteoclast-to-bone surface ratio (Ps.N.Oc/BS). (**D**) Calcein labeling in transverse methylmethacrylate (MMA) sections of p21 tibias, depicting cortical bone at midshaft. Scale bar: 200 μm. (**E**) Goldner’s trichrome staining of longitudinal MMA sections of p21 tibias, showing cortical bone at midshaft. White arrows indicate the presence (CTRL) or virtual lack (TFAM) of osteoid. Scale bar: 100 μm. (**F**) Dynamic histomorphometry analysis performed on transverse MMA sections of p21 tibias, quantifying periosteal double-labeled surface (Ps.dL.PM), mineralizing surface over bone surface (Ps.MS/BS), mineral apposition rate (Ps.MAR), bone formation rate over bone surface (Ps.BFR/BS), and osteoid accumulation (Ps.OS/BS). (**G** and **H**) RNAScope analysis conducted on longitudinal paraffin sections of p21 tibias, showing cortical bone at midshaft. The levels of expression of bone gamma-carboxyglutamate protein (*Bglap*) (**G**) and secreted phosphoprotein 1 (*Spp1*) (**H**) mRNAs were investigated, with quantification of the signal provided in both periosteum and endosteum. Scale bar: 100 μm. Evaluations included a minimum of 5 mice per group for both mutants and their respective CTRL littermates. Data are expressed as mean ± SD. Statistical analysis utilized Student’s 2-tailed *t* test, with significance denoted as *P ≤ 0.05, **P ≤ 0.01, ***P ≤ 0.001.

Micro-CT analysis of p21 tibias showed significant differences in C.Th and cortical bone mineral density in TFAM mutants compared with Het-TFAM and CTRL in both sexes (Figure 1, B and C, and Supplemental Figure 1, B and C). Of note, cortical bone surface over bone volume, an indirect index of osteoclast activity, was significantly higher in mutants than controls (Figure 1C and Supplemental Figure 1C). Given the absence of major sex-based differences in macroscopic characterization and micro-CT analysis, subsequent histomorphometry analysis of cortical bone was conducted only in females at p21. Moreover, only TFAM and CTRL littermates were investigated, as no macroscopic or micro-CT differences could be detected between CTRL and Het-TFAM mice. Histomorphometry verified that mutant cortical bone was thinner than controls (Figure 2, A and C). Strikingly, cellularity of the mutant periosteum was higher compared with controls. Both the absolute number of periosteal osteoblastic cells and the relative number of these cells over bone surface (Figure 2C), as well as periosteal osteoclasts and their relative number over bone surface (Figure 2, B and C), were increased in mutants. However, despite the apparent increase in osteoblastic cells, their activity was significantly reduced in mutants, as shown by dynamic histomorphometric parameters, such as periosteal double-labeled surface, mineralizing surface/bone surface, mineral apposition rate, and bone formation rate/bone surface (Figure 2, D and F). Consistent with these data, the accumulation of osteoid was virtually absent in the periosteum of mutant tibias compared with controls (Figure 2, E and F). Osteoid is newly deposited yet unmineralized bone. Different from the periosteum, histomorphometry differences between mutant and control endosteum were either minor and at the limit of statistical significance or virtually negligible (Supplemental Table 1).

In agreement with histomorphometry analysis, RNAScope data showed that the expression of bone gamma-carboxyglutamate protein (*Bglap*) mRNA was significantly downregulated in the mutant periosteum compared with control, whereas the expression of secreted phosphoprotein 1 (*Spp1*) mRNA was significantly higher (Figure 2, G and H). No detectable differences in levels of expression of either *Bglap* or *Spp1* mRNAs could be noted in the mutant endosteum (Figure 2, G and H). *Bglap* mRNA is a marker of mature osteoblasts, whereas *Spp1* mRNA is already present in osteoprogenitors. Therefore, terminal differentiation of periosteal osteoblastic cells was halted in mutants. This also suggests the increased number of osteoblastic cells detected by histomorphometry was most likely contributed by immature cells of the osteoblast lineage rather than mature osteoblasts.

Though the focus of our study was cortical bone, to be thorough, we also analyzed trabecular bone by histomorphometry. This verified a low–bone mass phenotype in mutants. The likely contributors to this phenotype included a decrease in osteoblast activity, as indicated by significantly reduced osteoid accumulation, despite no apparent change in the relative number of osteoblastic cells over bone surface, and an increase in the number of osteoclasts when corrected for bone surface (Supplemental Figure 2, A–C). A significant augmentation in cartilage remnants within bony trabeculae could also be detected in mutants (Supplemental Figure 2D), suggestive of a disorganized and altered transition from cartilage to bone.

Whole-mount Alizarin red/Alcian blue staining revealed that the parietal bones were hypomineralized, and their osteogenic front was considerably delayed in mutant mice compared with controls (Supplemental Figure 3A). Since parietal bones develop through an intramembranous process, this finding indicates that the bone phenotype observed in the cortical compartment of long bones was not secondary to the altered growth plate development.

Last, no abnormalities could be detected by either micro-CT or routine histology in mutant vertebrae at p21 (Supplemental Figure 3, B–D). PRX lineage cells do not contribute to vertebrae development. This finding indicates that the long bone phenotype in TFAM mutants is not secondary to a circulating factor.

Together, our data show that a) loss of TFAM in PRX lineage impairs bone mass accrual in both the trabecular and cortical compartments of long bones irrespective of sex; b) it causes spontaneous midshaft fractures by p21; c) these fractures are most likely secondary to severe thinning of cortical bone; and d) the thinning of cortical bone is due to a dramatic decrease of periosteal bone formation secondary to impaired terminal differentiation of osteoblastic cells in the periosteum, though increased bone resorption may contribute as well.

### TFAM loss in periosteal cells expands the skeletal progenitor pool, hampers osteoblast differentiation, and triggers a cellular stress response.

Although TFAM does not directly function as a nuclear transcription factor, alterations in OxPhos function may indirectly affect the nuclear transcriptome through various mechanisms, including the modulation of ATP levels. To delve deeper into the effects of TFAM loss on periosteal cell activity, we conducted unbiased single-cell RNA-sequencing (scRNA-Seq) analysis on periosteal cells harvested from CTRL and TFAM mutant hind limbs at p21. The assay was performed in biological duplicates. Control samples comprised 1 male and 1 female, whereas mutant samples were both female. Importantly, bioinformatic analysis comparing the 2 control samples to each other confirmed that the transcriptome of the 2 samples showed negligible differences (data not shown).

**Figure 3:**
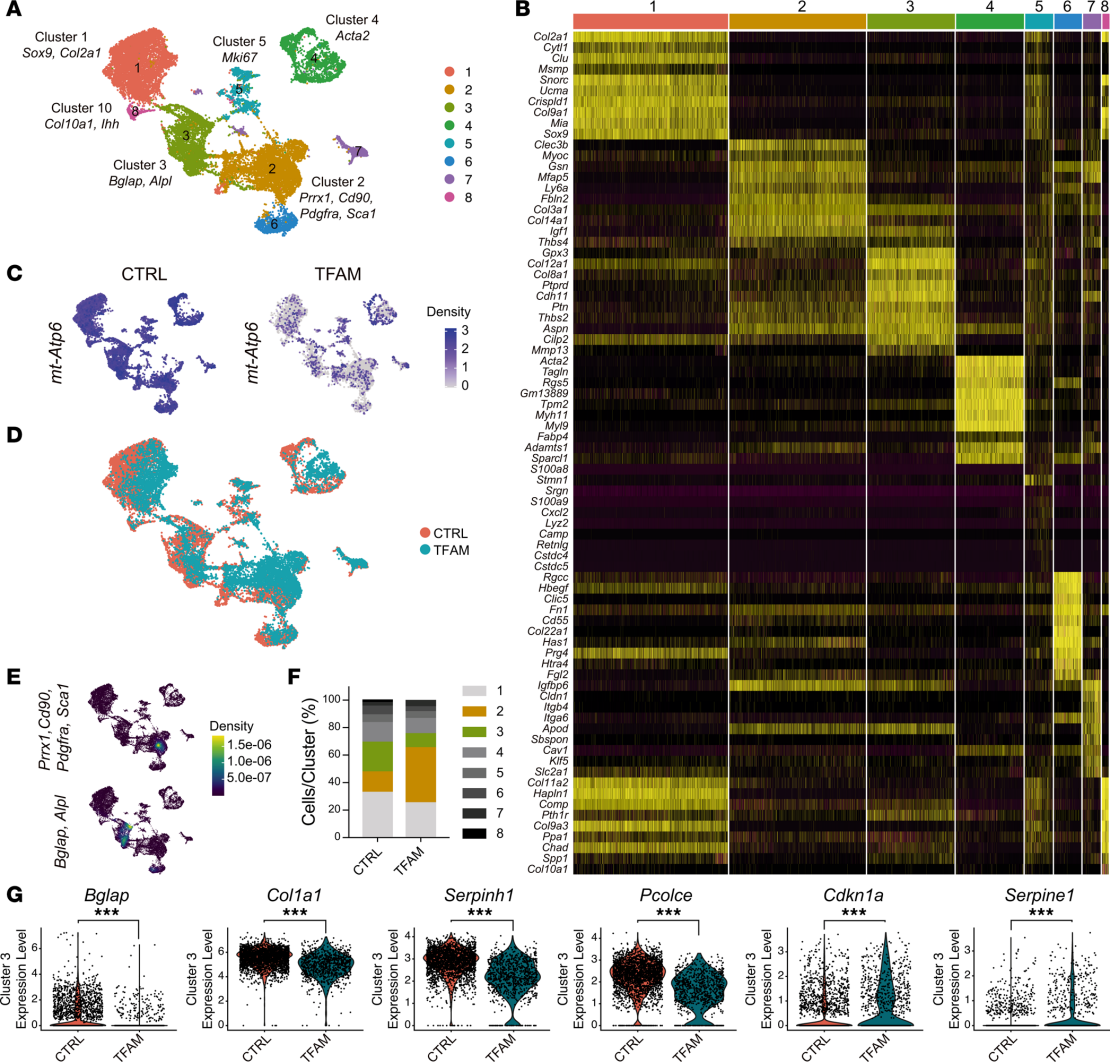
scRNA-Seq analysis of CTRL and TFAM mutant periosteal cells. (**A**) Uniform manifold approximation and projection (UMAP) visualization of aggregate clusters. (**B**) A heatmap displaying the top 10 genes expressed in each cell subcluster. (**C**) UMAP visualization of the mitochondrial marker ATP synthase subunit 6 (*mt-Atp6*), a downstream target of TFAM. (**D**) UMAP analysis depicting the distribution of cell populations across clusters, comparing CTRL cells (in orange) with TFAM mutant cells (in light blue). (**E**) Nebulosa plot illustrating the UMAP distribution of cells coexpressing skeletal progenitor markers such as paired related homeobox 1 (*Prx1*), Thy-1 cell surface antigen (*Cd90*), platelet-derived growth factor receptor alpha (*Pdgfra*), and stem cell antigen-1 (*Sca1*) (skeletal progenitors) alongside cells coexpressing *Bglap* and alkaline phosphatase (*Alpl*) (osteoblast-like cells). (**F**) A bar graph comparing the percentage of periosteal cell subtypes in CTRL versus TFAM, with skeletal progenitors highlighted in brown and osteoblast-like cells in green. (**G**) Violin plots illustrating the expression of representative markers differentially expressed between CTRL and TFAM, including *Bglap*, collagen type I alpha 1 chain (*Col1a1*), serpin family H member 1 (*Serpinh1*), procollagen C-endopeptidase enhancer (*Pcolce*), cyclin-dependent kinase inhibitor 1A (*Cdkn1a*), and serpin family E member 1 (*Serpine1*). The data in the violin plots are shown as distributions, with the width of the plot representing the density of data points at different expression levels. Biological and technical duplicates were used in the analysis. Statistical analysis employed Wilcoxon’s signed-rank test, with significance denoted as ***P ≤ 0.001.

Cellular aggregates of CTRL and TFAM were generated, and 16 distinct clusters at a resolution of 0.1 were identified (Supplemental Figure 4A). To specifically target periosteal cells, we then pursued an iterative process aimed at eliminating clusters expressing *CD45* and *CD31* mRNAs, namely hematopoietic and endothelial cells, and clusters with no significant decrease in mitochondrial gene expression in mutant cells, indicative of persistent TFAM activity and thus inefficient deletion of the TFAM-floxed allele (Supplemental Figure 4, B–D). We successfully isolated 8 distinct periosteal cell clusters (Figure 3, A and B), which closely resembled those described in a recent study (15). Cluster 1 exhibited an enrichment of cells expressing *Sox9* and *Col2a1* mRNAs. Cluster 2 was characterized by a predominance of cells expressing *Prrx1*, *Cd90*, *Pdgfra*, and *Sca1* mRNAs (Figure 3E). In cluster 3, a distinctive expression pattern of *Bglap* and *Alpl* mRNAs was observed (Figure 3E). Cluster 4 was marked by the prevalent expression of *Acta2* mRNA and likely related to the Acta2 lineage cells recently reported (16). Cluster 5 contained a highly proliferative cell population, as evidenced by the expression of *Mki67* mRNA (data not shown). Last, cluster 8 was enriched in *Col10a1* and *Ihh* mRNAs (data not shown). As outlined above, this degree of heterogeneity was consistent with recently published findings (15). Validation of TFAM recombination in the mutant cells was achieved by analyzing the expression of downstream mitochondrial genes of TFAM (Figure 3C).

Although cluster distribution remained consistent across CTRL and TFAM groups (Figure 3D), our data revealed a significant expansion of cluster 2 and a concomitant reduction of cluster 3 in mutants (Supplemental Table 2 and Figure 3F).

Gene expression analysis also showed significant differences between CTRL and TFAM cell populations (Supplemental Table 3). Using a threshold of 1.5-fold change, the expression of 280 and 247 genes was significantly upregulated in CTRL and TFAM, respectively (Supplemental Table 3).

The expression of numerous genes associated with osteoblast differentiation and collagen posttranslational modifications was found to be upregulated in the CTRL group when compared with TFAM within cluster 2 or cluster 3. Specifically, there was a significant increase in *Bglap* and *Bglap2* mRNAs in CTRL cluster 3 compared with TFAM, indicating a significant impairment in osteoblastic differentiation in TFAM periosteal cells (Supplemental Table 3 and Figure 3G). Additionally, levels of *Col1a1* and other collagen-related mRNAs were higher in CTRL clusters 2 and 3 (Supplemental Table 3 and Figure 3G).

The expression of the *Serpinh1* gene, known for its role in the proper folding and assembly of collagen molecules, was higher in CTRL clusters 2 and 3 versus TFAM (Figure 3G and Supplemental Table 3). This upregulation suggested the presence of aberrant collagen posttranslational modifications in TFAM mutants, potentially compromising bone collagen integrity, akin to observations in osteogenesis imperfecta (OI) models with *Serpinh1* mutations (17). Moreover, the expression of *Pcolce* gene, crucial for collagen fibril assembly and bone mechanical properties (18), was upregulated in both CTRL clusters (Supplemental Table 3 and Figure 3G). Additionally, secreted protein acidic and rich in cysteine (*Sparc*) mRNA was also expressed at higher levels in CTRL clusters 2 and 3 when compared with TFAM mutants (Supplemental Table 3). SPARC is integral to bone mineralization and matrix/cell signaling, with osteoblasts from patients with OI typically exhibiting reduced SPARC expression (19). Furthermore, mutations in SPARC have been identified in cases of recessive OI (19). Last, an upregulation of *Cdkn1a* mRNA expression was documented in TFAM cells across all clusters (Supplemental Table 3 and Figure 3G) along with *Serpine1* mRNA, whose expression was elevated particularly in clusters 2 and 3 of TFAM mutants. These findings are suggestive of an activation of the TGFB pathway in mutant cells as both genes are downstream targets of TGFB signaling, a hallmark of OI (20). To further investigate TGFB signaling, we performed immunohistochemistry (IHC) for SMAD2 and phosphorylated (phospho-) SMAD2, critical components of the canonical TGFB signaling pathway. Our results revealed no significant difference in SMAD2 and phospho-SMAD2 levels between mutant and control periosteal cells, suggesting that canonical TGFB signaling is not altered in these cells (Supplemental Figure 5, A and B).

A variety of genes were found to be overexpressed in TFAM mutants as well. The upregulation *Spp1* mRNA in TFAM mutant osteoblast-like cells (Supplemental Table 3), which aligned with the RNAScope findings (Figure 2H), could be indicative of either impaired differentiation of osteoprogenitor cells into mature osteoblasts or higher cellular stress signaling (21). Additionally, the upregulation of a group of stress response genes such as *metallothionein 1* and *2* (22), immediate early response gene 3 (*Ier3*), and Krüppel-like factor 4 (*Klf4*) mRNAs was suggestive of a coordinated stress cellular response to TFAM deficiency (Supplemental Table 3) (22, 23). Along those lines, the general upregulation of ribosomal genes (Supplemental Table 3) in TFAM mutant cells could be indicative of a compensatory mechanism to maintain protein synthesis despite mitochondrial impairment but could also reflect a stress response, as ribosomal proteins have been implicated in cellular stress signaling pathways (24). Consistent with a cellular stress response in TFAM-deficient cells, the expression of the heat shock protein A (HSPA) family of genes, particularly *Hspa9*, known for its chaperone activity in protein folding and protection against stress, was upregulated across all clusters, aligning with the need for enhanced protein quality control under stress conditions (Supplemental Table 3) (25). Last, the transcription factor activating transcription factor 3 (*Atf3*), which is known to be responsive to various stressors (26), emerged as one of the most notably upregulated genes in TFAM cells, particularly within cluster 2 (Supplemental Table 3). Curiously, we also noted higher levels of *Tnfsr11b* (*Opg*) mRNA in control cluster 1 when compared with the corresponding TFAM mutant cluster, with no changes of *Rankl* mRNA levels between mutant control groups across all clusters. Tnfsr11b acts as a decoy receptor for RANKL, thus preventing osteoclast maturation and bone resorption (27).

None of the mRNAs encoding glycolytic enzymes were increased in TFAM compared to control, with the exception of hexokinase 2 (*Hk2*), which was found to be increased by approximately 2-fold in cluster 2 (Supplemental Table 3). This finding indicates that changes in mRNAs encoding glycolytic enzymes do not contribute to the TFAM phenotype in periosteal cells.

Of note, scRNA-Seq analysis did not reveal significant changes in transcription factor *Runx2* mRNA expression between TFAM mutant and control cells.

Taken together, our scRNA-Seq data show that the loss of TFAM in periosteal cells expands the pool of cells enriched in skeletal progenitor markers, reduces the population of cells expressing osteoblastic markers, disrupts the expression of genes crucial for osteoblast maturation, and induces a broad cellular stress response.

### TFAM loss decreases steady-state intracellular ATP levels in periosteal cells.

**Figure 4:**
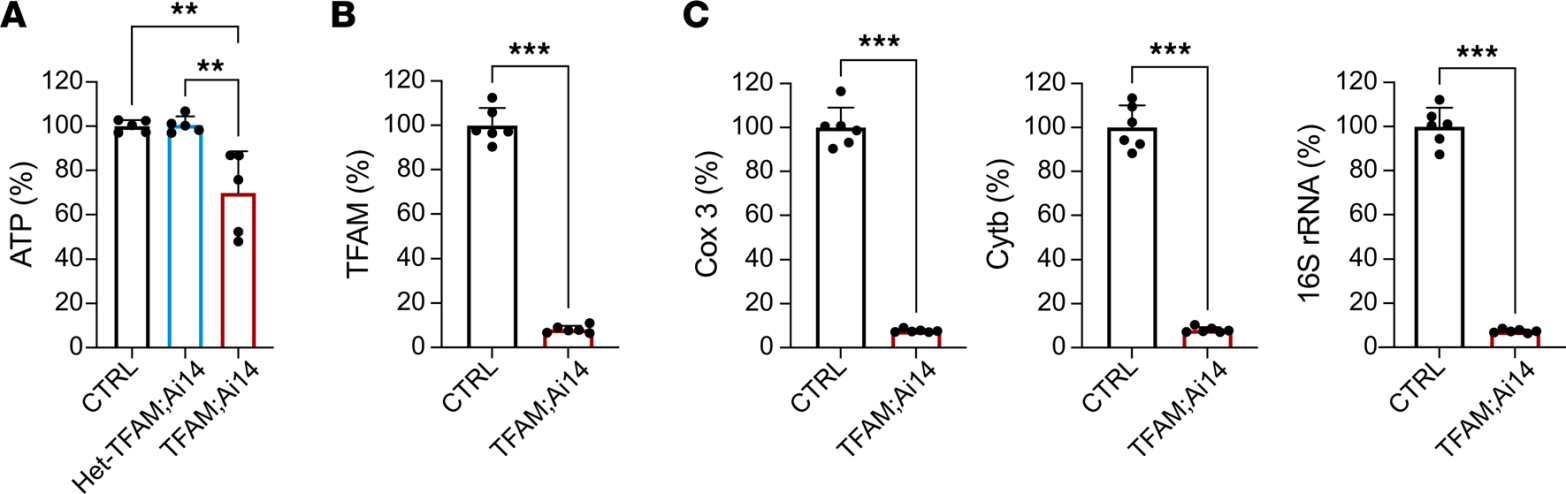
Loss of TFAM reduces steady-state intracellular ATP levels in periosteal cells. (**A**) Quantitative analysis of ATP concentrations in cultured periosteal cells selected by FACS for tdTomato expression. ATP levels were compared among 3 groups: *TFAM^fl/fl^ Ai14^fl/+^* (CTRL), *PRX TFAM^fl/+^ Ai14^fl/+^* (Het-TFAM Ai14), and *PRX TFAM^fl/fl^ Ai14^fl/+^* (TFAM Ai14) periosteal cells. (**B**) Quantification of the efficiency of TFAM-floxed allele recombination. (**C**) Quantification of mitochondrial genes by qPCR in CTRL and TFAM periosteal cells, focusing on cytochrome C oxidase 3 (*Cox3*), cytochrome B (*Cytb*), and 16S ribosomal RNA (*16S rRNA*) genes. Biological duplicates and technical duplicates or triplicates were used in experiments. Statistical significance was assessed using 1-way ANOVA with a Bonferroni post hoc test for comparisons among the 3 groups and an independent Student’s 2-tailed *t* test for pairwise comparisons. Data are presented as mean ± SD and normalized to CTRL levels to accommodate experimental variability. Significance levels are indicated as **P ≤ 0.01 and ***P ≤ 0.001.

To investigate whether the cortical bone phenotype observed in TFAM mutant mice could be attributed to ATP deficiency, we isolated periosteal cells from p21 *PRX TFAM^fl/+^ Ai14^fl/+^* (Het-TFAM Ai14) and *PRX TFAM^fl/fl^ Ai14^fl/+^* (TFAM Ai14) mice and sorted them by flow cytometry based on tdTomato expression. To be thorough, we also assayed unsorted cells from *TFAM^fl/fl^ Ai14^fl/+^* (CTRL). Cells were cultured in vitro for 7 days, and steady-state intracellular ATP levels were subsequently measured. Loss of TFAM significantly diminished steady-state intracellular ATP levels compared with Het-TFAM cells, while data generated from CTRL samples were similar to those from Het-TFAM (Figure 4A). Efficient recombination of the TFAM-floxed allele was verified via 2LoxP quantitative PCR (qPCR) of genomic DNA (Figure 4B). Furthermore, the loss of TFAM activity was validated by qPCR analysis of mitochondrial genes, including cytochrome C oxidase 3 (*Cox3*), cytochrome B (*Cytb*), and 16S ribosomal RNA (*16S rRNA*) genes (Figure 4C).

These findings underscore the crucial role of TFAM in maintaining normal ATP levels in periosteal cells, with its deficiency resulting in a significant bioenergetic deficit.

ATP deficiency did not lead to increased periosteal cell death in vivo, as evidenced by TUNEL staining (Supplemental Figure 6, A and B). Conversely, in vitro experiments demonstrated a significant increase in cell death among TFAM mutant periosteal cells by day 9, as verified by the trypan blue exclusion assay (Supplemental Figure 6C). By day 15, nearly all surviving cells were identified as wild-type because of the absence of recombination in the floxed allele in mutant cultures (Supplemental Figure 6, D and E). This outcome underscores the challenges in maintaining mutant cell viability in vitro over time. The reason for the discrepancy between the in vivo and in vitro behaviors of mutant cells remains elusive. Given the difficulties associated with culturing mutant periosteal cells in vitro, further exploration into their differentiation in vitro was not pursued.

Lowering ATP levels could increase AMPK activity and subsequently enhance degradation of Runx2, i.e., the master transcription factor of osteoblastogenesis, as previously reported (28). To explore the possibility that the TFAM bone phenotype could be mediated by an increased AMPK activity, we generated a mouse model lacking TFAM and carrying only 1 AMPK allele (*Prx1Cre TFAM^fl/fl^ AMPK^fl/+^*). Loss of 1 AMPK allele did not rescue the spontaneous fractures of TFAM mutant mice. Additionally, as expected since AMPK is a stress sensor, homozygous loss of AMPK worsened the TFAM phenotype (Supplemental Figure 7, A and B). Due to the lack of improvement, detailed micro-CT and histomorphometry analyses of *Prx1Cre TFAM^fl/fl^ AMPK^fl/+^* were not further pursued.

### Increasing HIF1 activity in PRX lineage cells prevents the low cortical bone mass and the spontaneous fractures observed in TFAM mutant mice.

Although impairment of OxPhos is the best characterized biological consequence of loss of TFAM across a large variety of tissues and cell types, TFAM also controls replication of mitochondrial DNA (mtDNA) (10), and mitochondria have functions that go beyond OxPhos and ATP production (29). Therefore, to establish if the impairment of OxPhos and, thus, the decreased intracellular ATP is the primary cause of the TFAM bone phenotype, we asked whether correcting the ATP levels through forced upregulation of glycolysis and lactate fermentation would prevent the low bone mass of TFAM mice. For this purpose, we exploited the fact that HIF1, a mediator of the cellular adaptation to low oxygen tension (30), promotes glycolysis and impairs OxPhos in virtually all the cell types and tissues where these metabolic pathways have been investigated (30). Therefore, we took advantage of the *HIF1dPA^fl/fl^* mice, in which a human cDNA encoding the HIF1dPA mutant protein is knocked into the ROSA26 locus and preceded by a stop cassette flanked by LoxP sites (31). HIF1dPA mutant protein cannot be hydroxylated by the oxygen sensors prolyl-4-hydroxylases 1, 2, and 3 (PHDs) because the 2 prolines that are the target of the PHDs are mutated to alanines. Therefore, HIF1dPA escapes proteasomal degradation, resulting in constitutive activation of HIF1 signaling, regardless of oxygen tension (31). Transcriptional properties of this mutant protein are indistinguishable from those of wild-type HIF1 (31).

First, we constitutively stabilized HIF1 in uncommitted mesenchymal progenitors of the limb bud and their descendants in vivo using the PRX transgenic mouse model. To achieve this, we crossed PRX mice with *HIF1dPA^fl/fl^* mice, resulting in the generation of *PRX HIF1dPA^fl/fl^* (HIF1dPA), *PRX HIF1dPA^fl/+^* (Het-HIF1dPA), and *HIF1dPA^fl/fl^* (CTRL) mice. Mutant and CTRL mice were born at the expected Mendelian frequency. At p21, macroscopic appearance, x-ray images, and body weight were similar across the groups in both females and males (Supplemental Figure 8, A and C, and Supplemental Figure 9, A and C).

Tibias were then isolated from p21 HIF1dPA, Het-HIF1dPA, and CTRL littermates, and analysis was performed on at least 5 mice from each group and sex according to the flowchart pursued for the analysis of TFAM mutants. Micro-CT analysis of cortical bone revealed HIF1dPA mutants, Het-HIF1dPA, and CTRL were virtually indistinguishable, regardless of sex (Supplemental Figure 8, B and C, and Supplemental Figure 9, B and C). Histomorphometric analysis of cortical bone performed exclusively in HIF1dPA and CTRL female specimens verified the micro-CT findings (Supplemental Figure 10, A and C; Supplemental Table 4; and Supplemental Figure 11, A–C). Furthermore, no differences were observed in indices of bone formation and matrix deposition (Supplemental Figure 10, D–F). Additionally, RNAScope analysis showed no difference in the expression levels of *Bglap* or *Spp1* mRNAs in either periosteum or endosteum between HIF1dPA and CTRL mice (Supplemental Figure 10, G and H). Likewise, no obvious abnormalities in the blood vessel network could be detected in HIF1dPA mutants by routine histology (data not shown).

Notably, in line with a previous report (9), micro-CT analysis of tibias isolated from 3-month-old HIF1dPA, Het-HIF1dPA, and CTRL littermates verified that the expression of constitutively stabilized HIF1 in osteoblastic cells substantially increased bone mass in both the trabecular and cortical compartments of mutant mice (data not shown).

Taken together, unlike observations in older mice, increasing HIF1 activity in osteoblastic cells in young adult mice does not lead to a detectable augmentation of bone mass accrual.

Subsequently, we generated double-mutant mice lacking TFAM and expressing the constitutively stabilized HIF1 protein in mesenchymal progenitors of the limb bud and their descendants (*PRX TFAM^fl/fl^ HIF1dPA^fl/fl^*) (referred to as TFAM HIF1dPA). The absence of an overt phenotype in p21 HIF1dPA single-mutant mice proved advantageous, enabling us to analyze the bone phenotype of double mutants without the confounding effect of any potential abnormalities attributable to the HIF1dPA mutation. Tibias were isolated from p21 TFAM HIF1dPA double mutants, *PRX TFAM^fl/fl^ HIF1dPA^fl/+^* (PRX TFAM Het-HIF1dPA), and *TFAM^fl/fl^ HIF1dPA^fl/fl^* (CTRL) littermate controls and studied using x-ray and micro-CT. At least 5 mice from each group were investigated. Since the bone phenotype of TFAM mutant males was similar to that observed in females, only females were analyzed.

**Figure 5:**
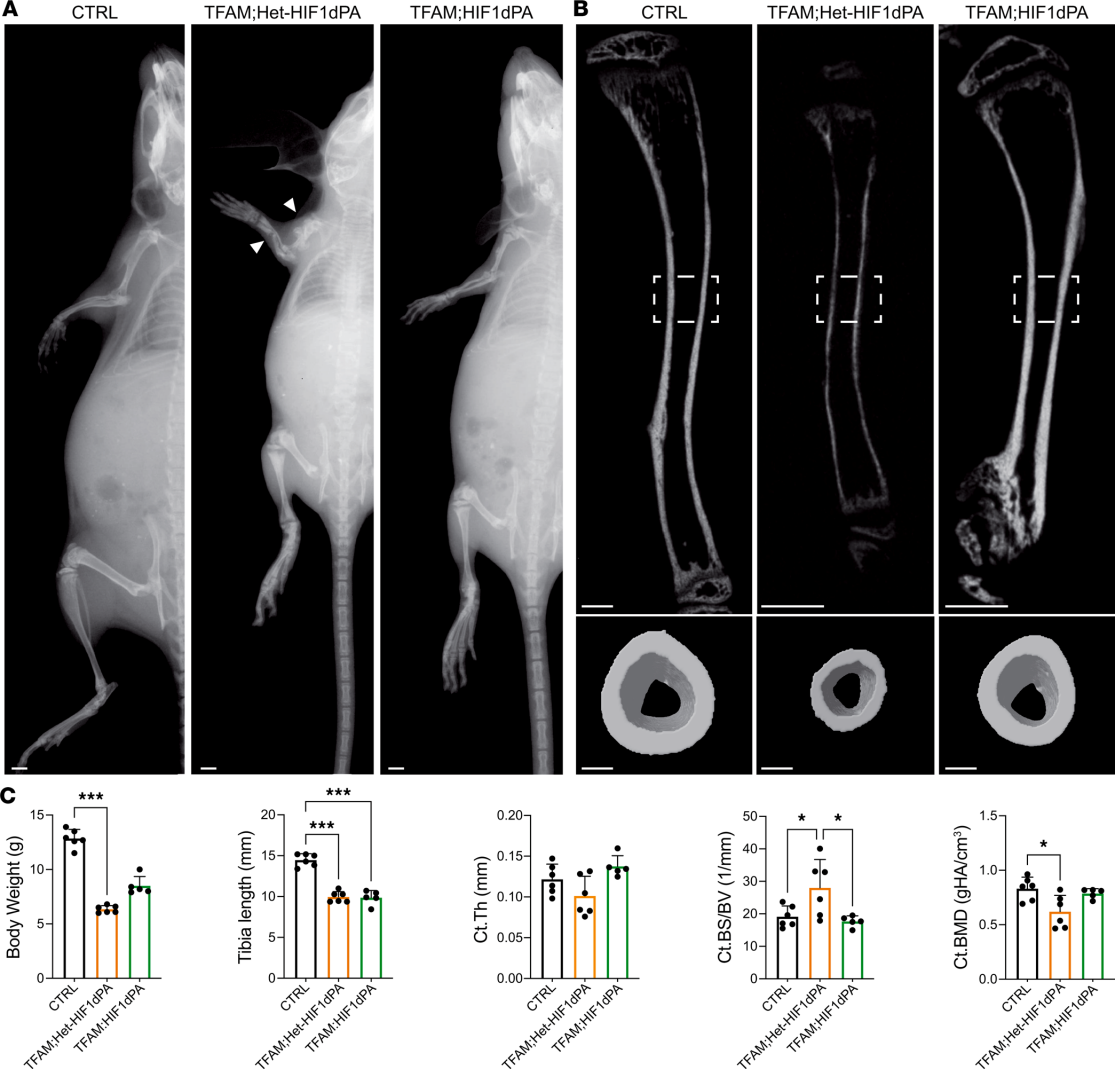
HIF1dPA corrects cortical defects observed in TFAM mutant mice but does not normalize their body weight and bone length. (**A**) X-ray images of p21 *TFAM^fl/fl^ HIF1dPA^fl/fl^* (CTRL), *PRX TFAM^fl/fl^ HIF1dPA^fl/+^* (TFAM Het-HIF1dPA), and *PRX TFAM^fl/fl^ HIF1dPA^fl/fl^* (TFAM HIF1dPA) mutant female mice. White arrows indicate multiple midshaft fractures in the forelimb of TFAM Het-HIF1dPA. Scale bars: 1 mm. (**B**) Longitudinal (top) and midshaft transverse (bottom) micro-CT scans of tibias isolated from CTRL, TFAM Het-HIF1dPA, and TFAM HIF1dPA p21 female mice. Scale bars: 500 μm for the longitudinal section and 100 μm for the transverse section. (**C**) Analysis of morphometric and micro-CT parameters, including body weight, tibia length, cortical thickness (Ct.Th), ratio of cortical bone surface to bone volume (Ct.BS/BV), and cortical bone mineral density (Ct.BMD) in CTRL, TFAM Het-HIF1dPA, and TFAM HIF1dPA p21 female mice. Scale bars: 100 μm. Evaluations were conducted on mutant mice and their respective CTRL littermates, with a minimum of 5 mice per group. Student’s 2-tailed *t* test was used to evaluate differences between groups, with data presented as mean ± SD. *P ≤ 0.05, ***P ≤ 0.001.

No spontaneous fractures were observed in p21 TFAM HIF1dPA double mutants (Figure 5A). Additionally, no significant differences were noted in indices of cortical bone mass between TFAM HIF1dPA double mutants and CTRL (Figure 5C). However, despite the absence of fractures, TFAM HIF1dPA double mutants were still smaller than controls, and their long bones were shorter and deformed (Figure 5, A–C), suggesting that growth plate abnormalities observed in TFAM mutants likely persisted in double mutants.

Like TFAM mutants and unlike PRX TFAM HIF1dPA mice, PRX TFAM Het-HIF1dPA mice exhibited multiple spontaneous fractures in the long bones of their limbs by p21 (Figure 5A). This observation demonstrated that with 1 gain-of-function HIF1dPA allele, the TFAM phenotype is not corrected, whereas homozygosity for the HIF1dPA allele fully rescues it. It is noteworthy that C.Th was slightly improved in PRX TFAM Het-HIF1dPA mice compared with TFAM mutants, though there was no improvement in bone mineral density (Figure 5, B and C). Given that PRX TFAM Het-HIF1dPA mice closely mirrored the TFAM phenotype, we did not include these mutants in our further analysis.

**Figure 6:**
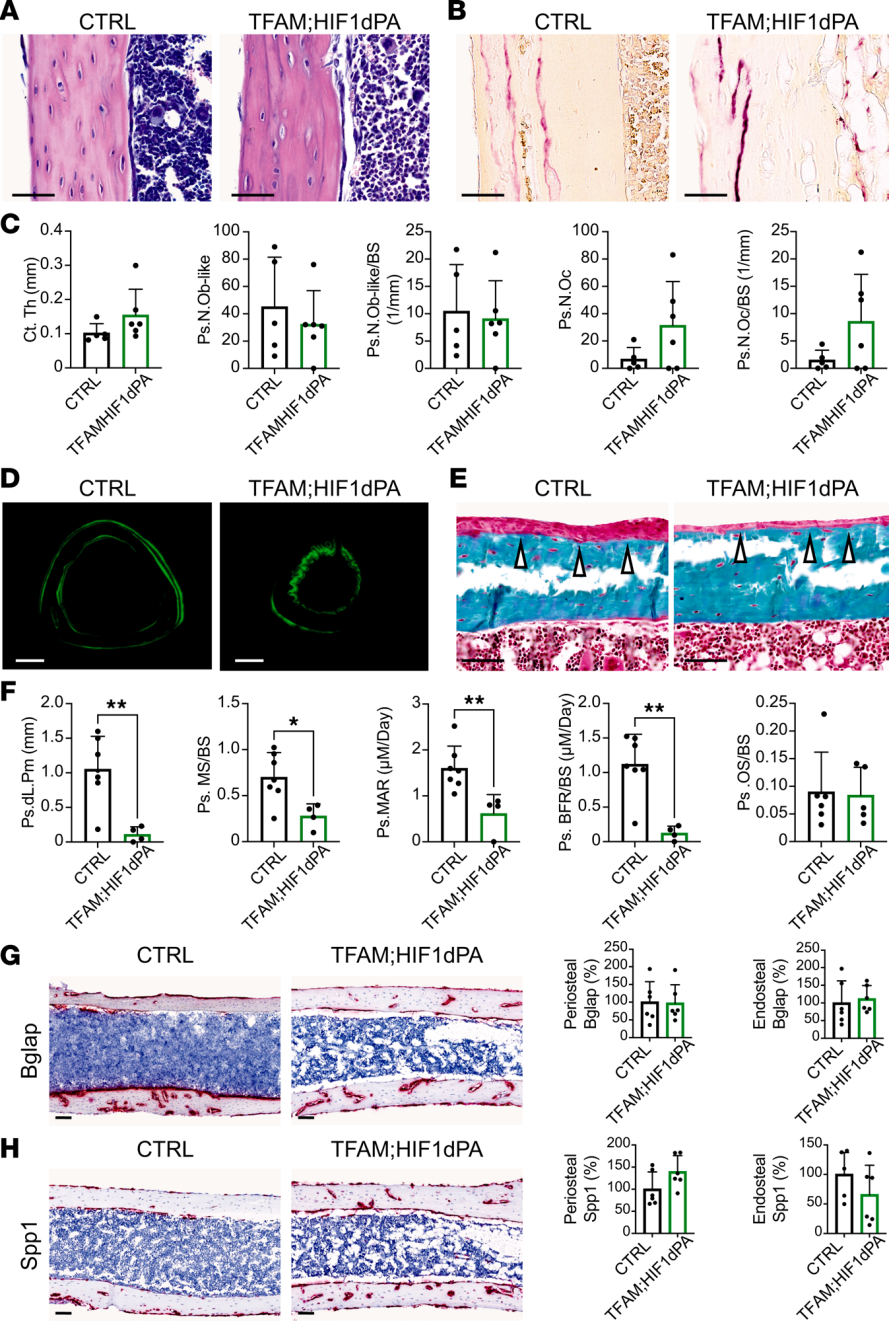
HIF1dPA largely corrects the cortical bone defects observed in TFAM mutant mice. (**A**) H&E staining of longitudinal paraffin sections of p21 tibias, illustrating cortical bone at midshaft. Scale bar: 100 μm. (**B**) TRAP staining of longitudinal paraffin sections of p21 tibias. Scale bar: 100 μm. (**C**) Static histomorphometry analysis conducted on longitudinal paraffin sections of p21 tibias, quantifying cortical thickness (Ct.Th), number of periosteal osteoblast-like cells (Ps.N.Ob-like), osteoblast-like cells per bone surface (Ps.N.Ob-like/BS), number of periosteal osteoclasts (Ps.N.Oc), and osteoclast-to-bone surface ratio (Ps.N.Oc/BS). (**D**) Calcein labeling in transverse MMA sections of p21 tibias isolated, depicting cortical bone at midshaft. Scale bar: 200 μm. (**E**) Goldner’s trichrome staining of longitudinal MMA sections of p21 tibias, showing cortical bone at midshaft. White arrows indicate the presence of osteoid. Scale bar: 100 μm. (**F**) Dynamic histomorphometry analysis performed on transverse MMA sections of p21 tibias, quantifying periosteal double-labeled surface (Ps.dL.PM), mineralizing surface over bone surface (Ps.MS/BS), mineral apposition rate (Ps.MAR), bone formation rate over bone surface (Ps.BFR/BS), and osteoid accumulation (Ps.OS/BS). (**G** and **H**) RNAScope analysis conducted on longitudinal paraffin sections of p21 tibias, demonstrating cortical bone at midshaft. The levels of expression of *Bglap* (**G**) and *Spp1* (**H**) mRNAs were investigated, with quantification of the signal provided in both periosteum and endosteum. Evaluations included a minimum of 5 mice per group for both mutants and their respective CTRL littermates. Scale bar: 100 μm. Data are presented as mean ± SD. Student’s 2-tailed *t* test was performed for statistical comparison. *P ≤ 0.05, **P ≤ 0.01.

Histomorphometric analysis verified that C.Th and periosteal cellularity, including both osteoblast-like cells and osteoclasts, were similar between TFAM HIF1dPA double mutants and controls (Figure 6, A–C, and Supplemental Table 5). Moreover, the ability of osteoblasts to produce and deposit matrix was comparable in double mutants and controls, as indicated by osteoid accumulation and RNAScope analysis of *Bglap* and *Spp1* mRNAs at the periosteal surface of cortical bone (Figure 6, E–H).

Surprisingly, however, indices of periosteal bone formation were consistently and significantly lower in TFAM HIF1dPA double mutants than in controls (Figure 6, D and F, and Supplemental Table 4).

Both histomorphometry and RNAScope did not reveal any substantial differences between TFAM HIF1dPA and CTRL cortical bone at its endosteal surface (Figure 6, G and H, and Supplemental Table 5). Moreover, trabecular bone indices of bone mass, number of osteoblast-like cells lining the bony trabeculae, and accumulation of osteoid were comparable across double mutants and controls (Supplemental Figure 12, A and C). However, different from cortical bone and similar to what was observed in TFAM mutants, the number of total osteoclasts and osteoclasts over bone surface were still significantly higher in double mutants (Supplemental Figure 12B). More importantly, a large number of cartilage remnants was present in the bony trabeculae of double mutants, indicating a disorganized transition from cartilage to bone and consistent with the shortening of TFAM HIF1dPA bones (Supplemental Figure 12D).

Taken together, our findings indicate that the low–bone mass phenotype and the spontaneous fractures caused by loss of TFAM, but not the shortening of the long bones, could be largely prevented by upregulating HIF1 activity in the same cells, though a significant impairment of bone formation rates persisted in the periosteum of double mutants.

### Increasing HIF1 activity in periosteal cells elevates glycolytic enzyme expression and partially reverses cellular and transcriptional changes caused by TFAM deficiency.

HIF1 is a pivotal nuclear transcription factor that induces the expression of a plethora of genes, crucial for diverse biological processes including glycolysis, bioenergetic metabolic reprogramming, angiogenesis, and posttranslational modifications of collagens (30, 32).

To explore whether HIF1 partially prevented the pronounced skeletal phenotype resulting from TFAM loss via transcriptomic changes, we performed comprehensive scRNA-Seq analyses on periosteal cells harvested from CTRL and TFAM HIF1dPA double-mutant hind limbs 3 weeks postnatally. The assay, carried out in biological duplicates, included both male and female samples in each group.

Echoing the methodology described earlier, cellular aggregates were generated, and 14 distinct clusters at a resolution of 0.1 were identified. As above, clusters expressing *CD45* and *CD31* mRNAs or with no significant differences in expression of mitochondrial genes between mutants and controls were excluded (data not shown).

**Figure 7:**
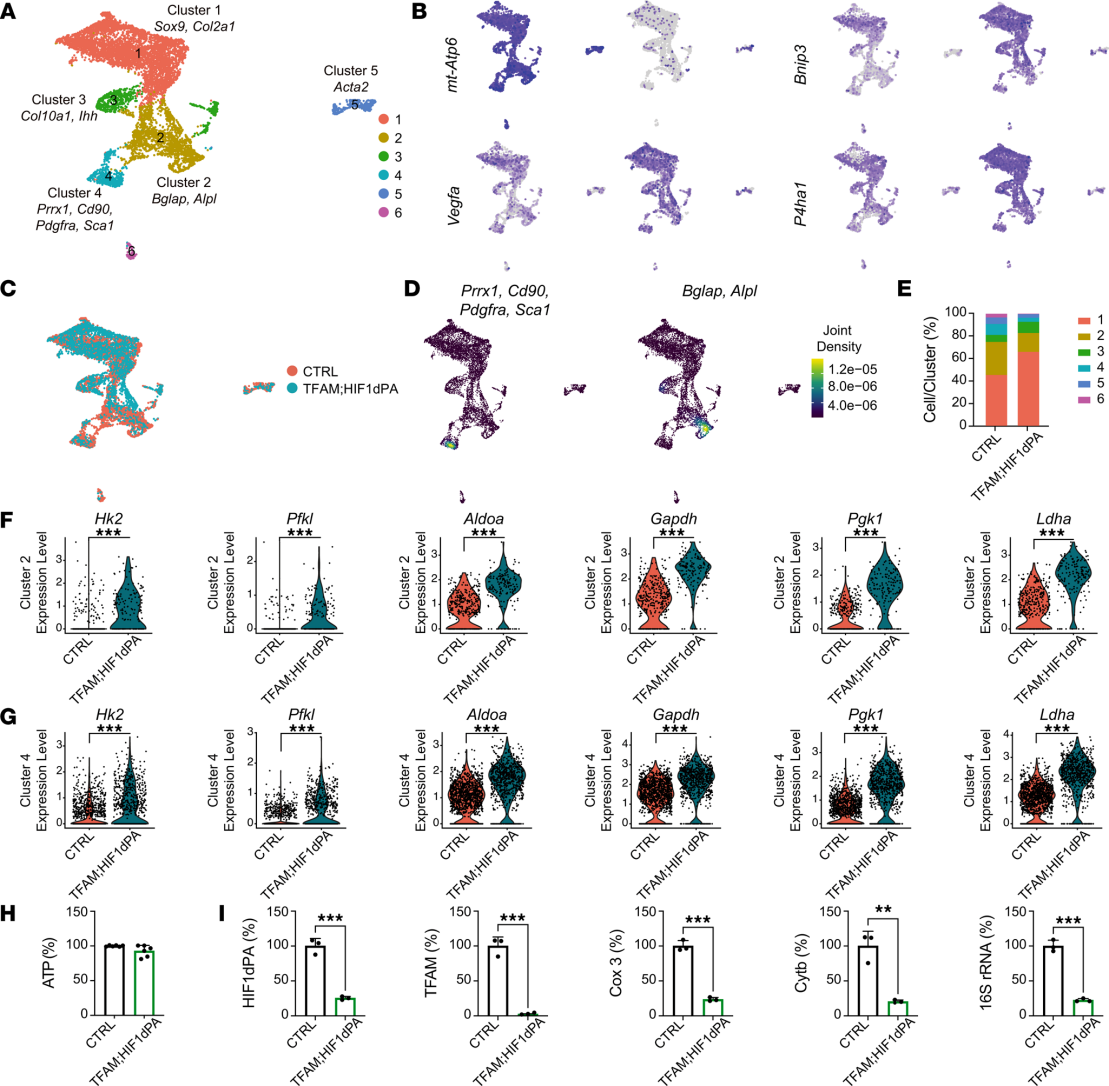
HIF1dpA enhances expression of glycolytic enzymes and restores ATP levels in TFAM-deficient periosteal cells. (**A**) UMAP visualization of aggregate clusters. (**B**) UMAP visualization of the mitochondrial marker ATP synthase subunit 6 (*mt-Atp6*), a downstream target of TFAM, and downstream targets of HIF1, including vascular endothelial growth factor A (*Vegfa*), Bcl2 interacting protein 3 (*Bnip3*), and prolyl-4-hydroxylase subunit alpha 1 (*P4ha1*). (**C**) UMAP analysis comparing the distribution of cells across clusters between CTRL (in orange) and TFAM HIF1dPA mutant (in light blue). (**D**) Nebulosa plots showing distribution of cells coexpressing skeletal progenitor markers — paired related homeobox 1 (*Prx1*), Thy-1 cell surface antigen (*Cd90*), platelet-derived growth factor receptor alpha (*Pdgfra*), stem cell antigen-1 (*Sca1*) — alongside osteoblast-like cell markers — bone gamma-carboxyglutamate protein (*Bglap*), alkaline phosphatase (*Alpl*). (**E**) Bar graph comparing percentage of periosteal cell subtypes in CTRL versus TFAM HIF1dPA, with cluster colors corresponding to UMAP distribution in **A**. (**F**) Violin plots showing increased expression of glycolytic markers in TFAM HIF1dPA mutant skeletal progenitors compared with CTRL, including hexokinase 2 (*Hk2*); phosphofructokinase, liver type (*Pfkl*); aldolase A (*Aldoa*); glyceraldehyde-3-phosphate dehydrogenase (*Gapdh*); phosphoglycerate kinase 1 (*Pgk1*); and lactate dehydrogenase A (*Ldha*). The data in the violin plots are shown as distributions, with the width of the plot representing the density of data points at different expression levels. (**G**) Violin plots illustrating increased expression of glycolytic markers in TFAM HIF1dPA mutant osteoblasts compared with CTRL. (**H**) Quantitative analysis of ATP in cultured CTRL and TFAM HIF1dPA periosteal cells. (**I**) Assessment of recombination efficiency at the ROSA26 locus in TFAM HIF1dPA and TFAM-floxed alleles. qPCR analysis of mitochondrial gene expression — cytochrome C oxidase 3 (*Cox3*), cytochrome B (*Cytb*), 16S ribosomal RNA (*16S rRNA*) — in CTRL and TFAM HIF1dPA mutant periosteal cells. Biological and technical duplicates were used in the analysis. scRNA-Seq data were analyzed using Wilcoxon’s signed-rank test. For ATP measurement, statistical significance was determined using either Wilcoxon’s matched pairs signed-rank test or an unpaired 2-tailed Student’s *t* test. Data were expressed as mean ± SD and normalized to CTRL levels. **P ≤ 0.01, ***P ≤ 0.001.

Consistent with our prior results, we identified 6 periosteal cell clusters, each defined by a unique gene expression signature as described above (Figure 7, A and D). We verified efficient TFAM recombination through the analysis of TFAM-regulated mitochondrial genes (Figure 7B). Moreover, HIF1’s constitutive activation was substantiated by the extensive upregulation across all clusters of its immediate downstream target genes, such as *Vegfa*, *Bnip3*, and *P4ha1* (Figure 7B and Supplemental Table 7).

In the TFAM HIF1dPA double mutant, unlike single TFAM mutants, we observed no shift in cellular distribution between skeletal progenitors (cluster 4) and the osteoblast-like cell (cluster 2) (Figure 7, C and E, and Supplemental Table 2). Curiously, however, in the mutant, we detected an expansion of the cluster enriched in *Sox9* and *Col2a1* mRNAs (cluster 1) (Figure 7E and Supplemental Table 2).

Differential gene expression analysis highlighted significant differences between CTRL and TFAM HIF1dPA cells. With a 2-fold change threshold, there were 2,044 upregulated genes in CTRL and 1,450 in the double mutant, respectively (Supplemental Tables 6 and 7). The TFAM HIF1dPA mutant consistently showed an increase in glycolytic enzyme mRNA levels, such as *Hk2*, *Pfkl*, *Aldoa*, *Gapdh*, *Pgk1*, and *Ldha*, across skeletal progenitor and osteoblast clusters (Figure 7, F and G). This is suggestive of an enhancement of glycolysis because of HIF1 activation. Additionally, HIF1 activation reversed certain genetic changes due to TFAM loss, particularly in cluster 2. For example, the reduced *Bglap* mRNA levels previously noted in TFAM mutant osteoblast-like cells were not detected in the double mutants, suggesting a role for HIF1 in promoting osteoblast differentiation. Similarly, *Serpinh1* and *Sparc* mRNA levels, which were lower in TFAM mutant osteoblast-like cells, were likely similar between controls and double mutants as indicated by their absence from Supplemental Tables 6 and 7. This absence may hint at a partial remediation of some OI-like features through HIF1 overexpression. Nonetheless, certain abnormalities persisted in double-mutant osteoblast-like cells. For instance, elevated expression of *Pcolce* mRNA in CTRL osteoblast-like cells suggested a persistent dysfunction of double-mutant osteoblast-like cells in their ability to build an adequate extracellular matrix (Supplemental Table 6). This was further supported by the high expression levels of *Cdkn1a* and *Serpin1* mRNAs in double-mutant osteoblast-like cells, indicative of a significant activation of the TGFB pathway, as observed in TFAM mutants (Supplemental Table 7). Interestingly, despite RNAScope data indicating no discernible difference between CTRL and double-mutant periosteum with respect to *Spp1* mRNA expression (Figure 6H), a modest increase in *Spp1* mRNA was still detectable in the double-mutant osteoblast cluster (Supplemental Table 7). This discrepancy between datasets may stem from the differing sensitivities of the assays used. Notably, double-mutant osteoblast-like cells exhibited a distinct expression profile of collagen mRNAs. Controls displayed increased levels of *Col1a1*, *Col1a2*, *Col3a1*, *Col5a1*, *Col5a2*, *Col6a5*, *Col8a1*, *Col8a2*, *Col12a1*, *Col13a1*, and *Col16a1* mRNAs (Supplemental Table 6), which are primarily associated with fibrillar collagens integral to bone formation and maintenance and thus reflective of standard osteoblastic activity (33). Conversely, double-mutant osteoblast-like cells exhibited higher expression of *Col2a1*, *Col9a1*, *Col9a2*, *Col9a3*, *Col11a2*, and *Col20a1* mRNAs (Supplemental Table 7), coding for collagens typically found in cartilaginous matrices, suggesting an altered state of differentiation. In addition, a subset of genes associated with cellular stress remained upregulated in the double-mutant osteoblast-like cells. This includes genes such as *Ier3* and *Klf4* and the HSPA family of genes, especially *Hspa9* (Supplemental Table 7). Additionally, expression of *Atf3* mRNA was still high in double-mutant osteoblast-like cells when compared with controls (Supplemental Table 7). Moreover, as in TFAM osteoblast-like cells, an increase in ribosomal gene expression was also observed in double-mutant osteoblast-like cells (Supplemental Table 7).

In cluster 1, no increase in *Tnfsr11b* mRNA levels was detected compared to the TFAM HIF1dPA mutant cluster. In contrast, elevated *Tnfsr11b* expression was observed in TFAM HIF1dPA mutant osteoblast-like cells relative to CTRL.

Taken together, our scRNA-Seq data indicate that increasing HIF1 activity corrected the expansion of the skeletal progenitor population and the impaired osteoblast differentiation caused by loss of TFAM. This corrective action is supported by the normalization of the *Bglap* mRNA expression. However, activation of HIF1 did not fully normalize the expression of genes relevant to osteoblast activity, which is consistent with a reduced bone formation rate still detectable in double-mutant periosteum. Last, the partial correction of the cellular and molecular phenotype of TFAM mutants was associated with a dramatic upregulation of glycolytic enzymes in double mutants.

### Increasing HIF1 activity in periosteal cells normalizes the reduced levels of steady-state intracellular ATP observed in TFAM mutant cells.

We then investigated if increasing HIF1 activity could restore the lowered ATP levels observed in TFAM-deficient periosteal cells. It is important to note that both HIF1dPA and Ai14 reporter constructs are knockins at the ROSA locus, which precludes using Ai14’s reporter function for isolating TFAM HIF1dPA double-mutant periosteal cells via FACS. Consequently, we measured intracellular ATP in an unsorted population of periosteal cells harvested from both CTRL and double-mutant mice and cultured in vitro for a few days, as delineated in the Methods section. Supporting the hypothesis that heightened HIF1 activity enhances glycolysis, no significant difference in ATP levels was observed between CTRL and double-mutant cells (Figure 7H). qPCR verified the successful recombination of the floxed TFAM and HIF1dPA alleles (Figure 7I). To rigorously compare ATP levels in TFAM and TFAM HIF1dPA mutant periosteal cells, intracellular ATP was also quantified in unsorted TFAM mutant and CTRL cells. This verified the initial finding; i.e., ATP levels in TFAM mutants were significantly diminished compared with controls (Supplemental Figure 13, A and B, with panel B showing the efficient recombination of TFAM allele), echoing the results from FACS-sorted cells (Figure 4A).

In summary, our findings demonstrate that enhancing HIF1 activity in periosteal cells effectively normalizes the altered steady-state intracellular ATP concentrations identified in TFAM mutant cells.

## Discussion

Our study presents a critical examination of the role of TFAM in the differentiation and function of periosteal osteoblasts and cortical bone mass accrual, offering insights into the bioenergetic demands of these skeletal cells. The loss of TFAM in mesenchymal progenitors of the limb bud and their progeny leads to a significant reduction in C.Th (Figure 1C) and an increased occurrence of spontaneous fractures, primarily due to disruptions in osteoblastic cell differentiation and impaired bone formation at the periosteum. This study represents the first comprehensive investigation to our knowledge into the role of TFAM and OxPhos in the biology of cortical bone and periosteal cells. Furthermore, while previous research has emphasized the essential role of OxPhos in providing energy for cellular differentiation both in vitro and in vivo, our findings also illuminate, for the first time to our knowledge, the compensatory mechanism by which upregulated glycolysis, mediated by HIF1 activity, can mitigate skeletal defects resulting from OxPhos impairments, highlighting the resilience and adaptability of skeletal cells.

A growing body of literature suggests that exposure to hypoxia may alleviate detrimental effects in experimental models of mitochondrial disorders. The mechanism by which this occurs, particularly whether it involves HIF1, is currently a topic of intense investigation (34). Our study unequivocally demonstrates that activation of HIF1 in skeletal precursors can compensate for OxPhos dysfunction, likely through the upregulation of glycolysis.

It has been recently reported that pharmacological inhibition of OxPhos in vitro in periosteal progenitors preserves their ability to proliferate and differentiate (35). The apparent discrepancy between these in vitro results and our in vivo findings accentuates the intricacy of living systems and reaffirms that in vitro conditions may not fully capture the vast network of microenvironmental factors that sculpt cellular conduct in a living organism.

It is crucial to note that deleting TFAM in PRX lineage cells results in the loss of TFAM across all chondro-osteoprogenitors during development, including growth plate chondrocytes. These cells are essential for the formation of the bone collar, which is the skeletal structure that initiates cortical bone development. Therefore, some of the periosteal abnormalities observed in the TFAM mutant mice may not be a direct result of TFAM loss in the periosteum alone. Instead, some of these abnormalities could be attributed to altered interactions between the growth plate chondrocytes and the bone collar.

Our scRNA-Seq data highlight the periosteal cell population’s diversity. In line with previous studies (15), our results verify the presence of a periosteal subpopulation enriched in *Col2a1* and *Sox9* mRNAs. Remarkably, under typical physiological conditions, chondrocytes are absent from the periosteum, suggesting that the implications of this discovery warrant further detailed exploration to elucidate its biological significance.

scRNA-Seq also suggests a pervasive stress response in TFAM mutant cells, as indicated by upregulated stress markers and chaperones in those cells. This is suggestive of a cellular environment grappling with bioenergetic stress. The upregulation of genes involved in the TGFB pathway hints at a compensatory mechanism aimed at mitigating the effects of stress and preserving cell viability, aligning with similar cellular responses observed in other systems under bioenergetic strain (36).

Although loss of TFAM profoundly impacts OxPhos, we recognize that TFAM influences mtDNA replication (10), and mitochondria possess roles beyond OxPhos and ATP synthesis (29). Nonetheless, the ability to substantially correct the severe periosteal phenotype by enhancing HIF1 activity, likely through an upsurge in glycolysis and lactate fermentation, clearly suggests that the low–bone mass phenotype associated with TFAM deficiency stems from OxPhos disruption and ATP deficiency. Moreover, it implies that, in principle, the HIF1 pathway could be targeted for therapeutic intervention in patients with mitochondropathies. Thus, our study opens the door to further exploration into how we can harness the plasticity of energy pathways to overcome genetic deficiencies.

However, not all transcriptomic changes and bone phenotype aspects, such as decreased bone formation rates, could be fully rectified by HIF1 activity enhancement. This suggests that TFAM loss might influence periosteal cell biology and cortical bone mass accrual through more complex molecular mechanisms that extend beyond ATP accumulation. Alternatively, there might be an interplay between TFAM loss and increased HIF1 activity that could limit the ability of HIF1 to completely counteract the cortical bone phenotype in TFAM mutants.

HIF1 is a transcription factor that directly controls the expression of a plethora of genes beyond those involved in the metabolic reprogramming, such as VEGF (37), which promotes angiogenesis, and genes implicated in the posttranslational modifications of collagens (38). Consequently, enhancing HIF1 activity may activate mechanisms other than the upregulation of glycolysis and normalization of ATP levels, potentially contributing to the HIF1-dependent mitigation of the TFAM phenotype. Notably, however, at 3 weeks of age, an increase in HIF1 activity does not seem to induce a phenotype on its own, yet it is capable of compensating for the TFAM deficiency. This observation strongly indicates that at this developmental stage, elevated HIF1 activity specifically addresses the deficits arising from the absence of TFAM. Given that a deficiency in TFAM leads to a marked reduction in intracellular ATP levels, which is averted by boosting HIF1 activity, it is highly plausible that the primary mechanism underlying the protective “rescue” effect of HIF1 is through the restoration of ATP concentrations.

At this stage, the direct link between ATP deficiency and defects in osteoblast differentiation and bone formation remains unclear. Wei et al. demonstrated that glucose uptake through Glut1 is crucial for suppressing AMPK activity and preventing Runx2 degradation (28). Therefore, it could be that ATP deficiency might activate AMPK, which would, in turn, enhance the degradation of Runx2, a key transcription factor essential for osteoblast differentiation and bone formation. However, in our model, the loss of 1 AMPK allele did not alleviate the bone defects associated with TFAM deficiency. This suggests that AMPK activation may not be the primary mechanism driving the bone phenotype in mice carrying the TFAM mutation. However, as discussed above, we cannot exclude the possibility that changes in intracellular ATP are not the only mechanism driving the TFAM phenotype.

Recent studies highlight the malate-aspartate shuttle’s (MAS) essential role in linking mitochondrial activity to glycolysis in skeletal cells (39). MAS facilitates the transfer of reducing equivalents (NADH) from the cytosol into the mitochondria, promoting OxPhos and efficient ATP production. Given that MAS relies on functional OxPhos, TFAM deficiency, by impairing this process, might be expected to reduce MAS activity and glycolytic flux. However, contrary to this expectation, it has been reported that impaired OxPhos leads to increased glycolysis and lactate fermentation, facilitated by a shift from TCA cycle glutamine utilization to reductive carboxylation, enabling NAD^+^ recycling from NADH (40).

Consistent with this conclusion, our recent publication (4) demonstrates that, unlike periosteal cells, growth plate chondrocytes lacking TFAM do not become ATP deficient, maintaining normal steady-state intracellular ATP levels. This observation suggests that periosteal cells, unlike chondrocytes, might lack mechanisms such as reductive carboxylation for NAD^+^ recycling, or they may exhibit higher ATP demands. To explore these differences, by conducting extensive metabolomics assays, would be informative. Unfortunately, the rapid decline in mutant periosteal cell viability after 7 days presented a significant challenge to these metabolic studies, rendering in vitro assays impractical at this stage.

scRNA-Seq analysis demonstrated the presence of a heterogeneous skeletal progenitor population within the periosteum, corroborating earlier findings (15). The biological significance of this heterogeneity, both under normal conditions and in pathological states such as fractures, remains to be determined. Intriguingly, activation of the HIF pathway within TFAM mutant cells not only rectifies the observed shift from osteoblast-like cells to progenitor-like cells in the mutant periosteum but also promotes the expansion of a chondrocyte-like cell cluster at this site. This aligns with prior observations that HIF plays an essential role in chondrocyte biology, with increased HIF activity inducing ectopic cartilage formation in the periosteum and perichondrium of developing mutant bones (41).

Our study also underscored an elevated number of osteoclasts in the periosteum of TFAM mutant regions where osteoclasts are scarcely found under normal conditions in growing animals. This aberrant phenotype was ameliorated by enhancing HIF1 activity. Importantly, scRNA-Seq analysis showed that loss of TFAM leads to a reduction in *Tnfsr11b* mRNA in chondrocyte-like cells without affecting *RANKL* mRNA expression. In contrast, HIF1dPA expression normalized *Tnfsr11b* levels in TFAM-deficient chondrocytes while increasing *Tnfsr11b* expression in mutant osteoblasts. Considering Tnfsr11b’s critical role in repressing osteoclast development, it is tempting to speculate that chondrocyte-like cells in the periosteum might possess a biological function linked to the regulation of osteoclastogenesis in this niche. Nevertheless, further investigation is necessary to substantiate this intriguing possibility.

In this study, we primarily addressed the consequences of TFAM loss in periosteal cells; however, we extended our investigation to the histomorphometric analysis of the endosteal surface, encompassing both cortical and trabecular bone. This analysis yielded results analogous to those observed at the periosteum, though the effects at the endosteum of cortical bone were decidedly less pronounced. Currently, we cannot provide a definitive explanation for this variation. Nonetheless, this finding underscores the biological distinction between the endosteum and periosteum and emphasizes the significance of cellular context and local microenvironments in influencing the outcomes of genetic mutations.

Our findings show that developing bones deficient in TFAM within mesenchymal progenitors of the limb bud and their progeny are shorter and fragile. Moreover, they exhibit diminished osteoblast activity, increased number of osteoclasts, and higher expression of TGFB downstream targets, particularly at the periosteal site. Remarkably, this phenotype bears a significant resemblance to the traits observed in murine models of OI, also known as brittle bone disease (42). OI is a genetic condition marked by easily fractured bones because of mutations that result in an aberrant collagen matrix. TGFB signaling has been identified as a contributory factor in OI pathophysiology (20). Our results introduce the compelling prospect that modulating bioenergetic metabolism may have therapeutic benefits in pathological states characterized by bone fragility, such as OI. Furthermore, despite the upregulation of downstream TGFB targets in the mutant cells, our IHC analysis for SMAD2 and phospho-SMAD2 showed no significant difference between mutant and CTRL periosteal cells. This suggests that the periosteal phenotype may not be driven by changes in canonical TGFB signaling but potentially through noncanonical pathways or other signaling mechanisms.

Last, building upon previous findings by us and others (4, 43), we have corroborated that the depletion of TFAM in mesenchymal progenitors of the limb bud and their lineage leads to growth plate anomalies, resulting in shorter bones. Although our investigation did not primarily focus on the growth plate, we provide definitive evidence that this phenotype not only manifests postnatally but also is associated with the abnormal retention of cartilage remnants in the bone marrow cavities of mutant specimens. This occurs despite an increased number of osteoclasts. Remarkably, the shortening of the long bones and the continued presence of cartilaginous remnants, consequent to TFAM loss, were not mitigated by enhanced HIF1 activity. This indicates that the interplay between TFAM and HIF1 markedly differs between osteoblasts and chondrocytes in vivo. Along those lines, it is interesting to note that, as outlined above and unlike periosteal cells, chondrocytes lacking TFAM do not experience energy deficiency (4). Further studies are warranted to understand this dichotomy.

## Methods

Detailed information regarding materials and methods can be found in Supplemental Methods of the supplemental material.

### Sex as a biological variable.

Our study examined male and female animals, and similar findings were reported for both sexes.

### Statistics.

All data in this study are presented as mean ± SD. The assumption of normality was assessed using the Shapiro-Wilk test for both pairwise and multiple-group comparisons. Pairwise comparisons between 2 groups were conducted using a 2-tailed, unpaired Student’s *t* test if the data met the normality assumption. In cases where normality was not met, Wilcoxon’s rank-sum test was used. For analyses involving 3 or more groups, 1-way ANOVA was initially performed. If the data satisfied the assumption of normality, post hoc pairwise comparisons were adjusted using Bonferroni’s correction to control the family-wise error rate. Conversely, if the normality assumption was violated, the Kruskal-Wallis test was applied, followed by Dunn’s multiple-comparison test for post hoc analyses. Graphical representations and statistical analyses were completed using GraphPad Prism software (version 10.1.1). A *P* value of less than or equal to 0.05 was considered statistically significant for all tests.

### Study approval.

All procedures involving mice were conducted in accordance with the NIH *Guide for the Care and Use of Laboratory Animals* (National Academies Press, 2011) and were approved by the University of Michigan IACUC (Protocol number: PRO00007215) and the University of Pennsylvania IACUC (Protocol number: 806981).

### Data availability.

In alignment with the NIH data-sharing policies, the scRNA-Seq have been deposited in NCBI’s Gene Expression Omnibus (GEO) and are accessible through GEO Series accession number GSE261114 (https://www.ncbi.nlm.nih.gov/geo/query/acc.cgi?acc=GSE261114). Values for all data points in graphs are reported in the Supporting Data Values file. Analytic codes generated during this study are deposited in the GitHub repository SchipaniLab (https://github.com/SchipaniLab/MPKhan-ESabini_2024; commit ID 2b634d2).

## Author contributions

MPK and E Sabini contributed to the conceptualization, data curation, formal analysis, investigation, methodology, validation, and writing of the original draft. KB contributed to the methodology. GL contributed to the review and editing of the manuscript. BL contributed to the methodology and review and editing of the manuscript. DW also contributed to the methodology. CM contributed to the review and editing of the manuscript. AG contributed to the visualization, review, and editing of the manuscript. FL contributed to the visualization, review, and editing of the manuscript. DT contributed to the methodology, visualization, review, and editing of the manuscript. E Schipani contributed to the conceptualization, funding acquisition, investigation, methodology, and resource management, as well as the manuscript’s supervision, visualization, writing, review, and editing.

## Supplementary Material

Supplemental data

Supporting data values

## Version 1 08/01/2024

In-Press Preview

## Version 2 09/24/2024

Electronic publication
