# Supplementary material for: HIF1 activation safeguards cortical bone formation against impaired oxidative phosphorylation
Source: JCI Insight. 2024 Aug 1;9(18):e182330. doi: 10.1172/jci.insight.182330 (PMC11457864; doi:10.1172/jci.insight.182330)
Supplement: Supplemental data [file jciinsight-9-182330-s027.pdf]

APPENDIX 1

MATERIAL AND METHODS

Generation of *PRX;TFAM$^{fl/fl}$*, *PRX;HIF1dPA$^{fl/fl}$*, *PRX;TFAM$^{fl/fl}$;HIF1dPA$^{fl/fl}$* and *PRX;TFAM$^{fl/fl}$;Ai14$^{fl/+}$* mutant mice.

Genotyping of *PRX* (*FVB/N*), *TFAM$^{fl/fl}$* (*C57BL/6*), *HIF1dPA$^{fl/fl}$* (*C57BL/6*), *Ai14$^{fl/fl}$* (*FVB/N*), and *AMPK$^{fl/fl}$* (*C57BL/6*) transgenic mice was conducted as previously described (1-5). *PRX* transgenic male mice were crossed with homozygous *TFAM$^{fl/fl}$* females to produce *PRX;TFAM$^{fl/+}$* male mice. These male offspring were then crossed with *TFAM$^{fl/fl}$* females to generate *PRX;TFAM$^{fl/fl}$* mutants as well as *PRX;TFAM$^{fl/+}$* and *TFAM$^{fl/fl}$* controls. For the generation of *PRX;HIF1dPA$^{fl/fl}$* mutant mice, PRX transgenic males were bred with homozygous *HIF1dPA$^{fl/fl}$* females to obtain *PRX;HIF1dPA$^{fl/fl}$* male mice. Subsequently, these male mice were mated with *HIF1dPA$^{fl/fl}$* females to produce *PRX;HIF1dPA$^{fl/fl}$* mutants along with *PRX;HIF1dPA$^{fl/+}$* and *HIF1dPA$^{fl/fl}$* controls. To generate *PRX;TFAM$^{fl/fl}$;HIF1dPA$^{fl/fl}$* mutant mice, *PRX* transgenic male mice were bred with homozygous *TFAM$^{fl/fl}$;HIF1dPA$^{fl/fl}$* females (obtained by mating *TFAM$^{fl/fl}$* males with *HIF1dPA$^{fl/fl}$* females) to yield *PRX;TFAM$^{fl/+}$;HIF1dPA$^{fl/+}$* male mice. These male mice were then crossed with *TFAM$^{fl/fl}$;HIF1dPA$^{fl/fl}$* females to produce *PRX;TFAM$^{fl/fl}$;HIF1dPA$^{fl/fl}$* double mutants, *PRX;TFAM$^{fl/fl}$;HIF1dPA$^{fl/+}$*, and *TFAM$^{fl/fl}$;HIF1dPA$^{fl/fl}$* controls. For the generation of *PRX;TFAM$^{fl/fl}$;Ai14$^{fl/+}$* mutant mice, *PRX* transgenic male mice were bred with homozygous *Ai14$^{fl/fl}$* females to obtain *PRX;Ai14$^{fl/+}$* male mice. These males were subsequently crossed with *TFAM$^{fl/fl}$* females to generate *PRX;TFAM$^{fl/fl}$;Ai14$^{fl/+}$* mutant reporter mice and *TFAM$^{fl/fl}$;Ai14$^{fl/+}$* controls. To generate *PRX;TFAM$^{fl/fl}$;AMPK$^{fl/fl}$* mutant mice, PRX transgenic male mice were bred with homozygous *TFAM$^{fl/fl}$;AMPK$^{fl/fl}$* females (obtained by mating *TFAM$^{fl/fl}$* males with *AMPK$^{fl/fl}$* females) to yield *PRX;TFAM$^{fl/+}$;AMPK$^{fl/+}$* male mice. These male mice were then crossed with *TFAM$^{fl/fl}$;AMPK$^{fl/fl}$* females to produce *PRX;TFAM$^{fl/fl}$;AMPK$^{fl/fl}$* double mutants and *TFAM$^{fl/fl}$;AMPK$^{fl/fl}$* controls.

All mice used in this study were obtained from The Jackson Laboratory, with the following stock numbers: *PRX* (005584), $TFAM^{fl/fl}$ (026123), $HIF1dPA^{fl/f}$ (009673), $Ai14^{fl/fl}$ (007914), and $AMPK^{fl/fl}$ (014141).

X-rays imaging.

Radiographic images of three weeks old mice were captured employing either a Faxitron X-ray cabinet or a digital microradiography system (Faxitron, Wheeling, IL, USA). Mice were subjected to an 8-second exposure of 20 keV radiation. Subsequently, the exposed films underwent development in a darkroom environment using a Hope MicroMax X-ray processor (Hope X Ray Products Inc., Warminster PA) and were scanned to facilitate quantitative analysis of fracture incidence.

Micro-CT analysis.

Tibiae harvested from three-week-old mice underwent meticulous dissection and cleansing to remove soft tissue while preserving the epiphyses. Subsequently, the left tibiae underwent scanning using a high-resolution micro-CT imaging system, the Bruker Skyscan 1176 (Bruker BioSpin, Kontich, Belgium), employing an isotropic voxel size of 9 μm, a rotation angle of 0.3°, 2-frame averaging, and a 0.5 mm aluminum filter, with a source voltage of 50 kV and a current of 800 μA. Subsequently, reconstruction of these images was performed using Skyscan NRecon software (Bruker BioSpin). Microarchitectural analysis was then conducted using CTAn software (Bruker BioSpin). Regions of interest (ROI) were delineated as a percentage of the total bone length, with cortical ROI lengths specifically defined as 10% of the overall bone length, centered at the midshaft of the tibiae.

Routine histology, H&E, TRAP, Safranin-O, RNAscope, Immunohistochemistry (IHC), and TUNEL assay.

Right tibiae from three-week-old mice were designated for comprehensive histological analyses, including H&E, Tartrate Resistant Acid Phosphatase (TRAP), Safranin-O, RNAscope, IHC stainings, and TUNEL assay. Initially, the specimens were fixed in a 4% paraformaldehyde (PFA) solution in phosphate-buffered saline (PBS).

Routine histology, H&E, TRAP, Safranin-O, RNAscope, IHC.

Following fixation, standard processing procedures were carried out, including paraffin embedding and longitudinal sectioning at a thickness of 5 microns, following established protocols (6). TRAP staining was performed according to the manufacturer's instructions (Sigma-Aldrich 387A-1KT). RNAscope analysis was conducted utilizing the RNAscope 2.5 HD Detection Reagent - RED kit (Advanced Cell Diagnostics, Hayward, CA; Catalog No. 322360); specific probes for mRNAs encoded by either *Bgalp* (Catalog No. 478941) or *Spp1* (Catalog No. 435191) genes were provided by the same company.

For IHC detection, paraffin sections were subjected to antigen retrieval using sodium citrate buffer at pH 6 and heat-treated at 95°C for 10 minutes. Subsequently, sections were incubated with primary antibodies, including anti-SMAD2 (12570-1-AP, Proteintech) at a dilution of 1:50 and anti-Phospho-SMAD2 (44-244G, Invitrogen) at a dilution of 1:20, overnight at 4°C. Following primary antibody incubation, sections were treated with the appropriate biotinylated secondary antibody (ab207995, Abcam) at dilution 1:1000 for 30 minutes at RT. Visualization was performed using the labeled streptavidin biotin (TSA) system according to the manufacturer's instructions (Perkin Elmer, Shelton CT, USA). Negative controls were included by omitting the primary antibody. The anti-Collagen I antibody (ab254113, Abcam) was used as a positive control.

TUNEL Assay.

The TUNEL assay was performed on fixed frozen specimens to identify apoptotic cells by labeling DNA strand breaks. Briefly, specimens were fixed in a (PFA) solution, as previously described.

Following fixation, the specimens were decalcified using an EDTA solution to remove mineral deposits, then embedded in an Optimal Cutting Temperature (OCT) compound. The embedded specimens were snap-frozen and stored at -20°C until further processing.

The TUNEL assay was conducted using an in situ Cell Death Detection Kit (Roche) according to the manufacturer's instructions and previously reported protocol (6). Images for the TUNEL assay were captured using filters for FITC (fluorescein isothiocyanate) and DAPI (4',6-diamidino-2-phenylindole) to visualize apoptotic nuclei and total nuclei, respectively.

Images of the stained sections with H&E, Safranin-O, and TRAP were captured using a Zeiss AxioScan Z1 slide scanner and 20X objective and then subjected to static histomorphometry analysis. RNAscope and IHC images were captured in a similar manner and the signal was quantified by ImageJ software (Version 2.14.0/1.54f) (7). TUNEL images were acquired using a Zeiss LSM 980 Confocal microscope.

Calcein labeling, generation of methylmethacrylate sections, and Goldner's trichrome staining.

Three-week-old mice were intraperitoneally injected with calcein (Sigma-C0875) at a dosage of 40 mg/kg, administered 10 and 3 days prior to euthanasia. Left tibiae, previously used for micro-CT, were embedded in methylmethachrylate (MMA) following previously described protocols (8). MMA-embedded samples were subsequently sectioned into 5- and 8-μm thicknesses using Polycut microtome (Reichert-Jung, Leica, Wetzlar, Germany). Sections of 5 μm thickness were stained using Goldner's trichrome staining, while the 8 μm sections were cover-slipped without staining for imaging under FITC-mounted filters to detect calcein. All images were acquired by using Zeiss AxioScan Z1 slide scanner and a 20X objective.

Histomorphometry.

Histomorphometric analysis of both trabecular and cortical bone was conducted on paraffin or methylmethacrylate sections of tibiae prepared as outlined previously (8). ROI was delineated as a percentage of the total bone length. Specifically, the trabecular ROI length was set at 15% of the total bone length, with its position defined by the closest edge located 5% away from the growth plate. Conversely, the cortical ROI was defined as 10% of the overall bone length, centered at the midsection of the tibiae. Histomorphometric measurements were carried out in a randomized and blinded manner using Bioquant Osteo software V17.2.6 (Bioquant Image Analysis Corp., Nashville, TN), following established protocols (8).

Static histomorphometry.

H&E stained sections were utilized to quantify several cortical and trabecular parameters. TRAP staining facilitated the visualization and accurate counting of osteoclasts in both cortical and trabecular bone. Safranin-O stained sections were employed to assess the presence of cartilage remnants within bony trabeculae. Furthermore, Goldner's trichrome staining of both trabecular and cortical undecalcified MMA sections enabled the measurement of the osteoid content.

Dynamic histomorphometry.

Calcein labels were examined throughout the entire cortex, including both periosteal and endosteal surfaces. Parameters such as single-labeled surface (sLS), double-labeled surface (dLS), mineralizing surface/bone surface (MS/BS), mineral apposition rate (MAR), and bone formation rate/bone surface (BFR/BS) were quantified from undecalcified, unstained methylmethacrylate sections.

Alcian Blue and Alizarin Red whole-mount staining.

The Alcian Blue and Alizarin Red S whole-mount staining procedures were conducted on newly born pups following previously established protocols (6).

Periosteal cells isolation.

Periosteal cells were isolated from hindlimbs of *TFAM*$^{fl/fl}$ and *PRX;TFAM*$^{fl/fl}$ mice, as well as from *TFAM*$^{fl/fl}$*;HIF1dPA*$^{fl/fl}$ and *PRX;TFAM*$^{fl/fl}$*;HIF1dPA*$^{fl/fl}$ mice at p21, following an established procedure (9). Briefly, femurs and tibias were dissected to remove muscle and connective tissues under aseptic conditions. The epiphyses were covered with 5% low melting point agarose (SeaPlaque, Lonza, Verviers, Belgium) to protect them from enzymatic degradation. Periosteal cells were isolated via collagenase-dispase enzymatic digestion, consisting of 3 mg/ml collagenase type IA (MilliporeSigma, Rockville, MD) and 4 mg/ml dispase 2 (MilliporeSigma) in a-minimal essential medium (a-MEM) with 2 mM glutaMAX-I (Gibco, Invitrogen, Carlsbad, CA). Cells from the initial 10-minute digestion phase were discarded to remove contamination with residual muscle and connective tissue cells. Subsequently, a one-hour digestion with collagenase was performed to release the periosteal cells. Efficient recombination of the TFAM and HIF1dPA floxed allele was quantified by 2-LoxP qPCR of genomic DNA (10). The sequences of the primers utilized for qPCR are provided in Supplemental Table 8.

Ai14/tdTomato-positive cell isolation.

Periosteal cells were isolated from p21 *PRX;TFAM*$^{fl/+}$*;Ai14*$^{fl/+}$ and *PRX;TFAM*$^{fl/fl}$*;Ai14*$^{fl/+}$ mice following the previously discussed protocol to ensure a single-cell suspension amenable to sorting. These cells were then subjected to FACS (FACSAria™ sorter, BD Biosciences). The tdTomato protein was excited at approximately 554 nm, and its emission was detected at approximately 581 nm. Post-sorting, cells were collected in a-minimal essential medium (a-MEM) supplemented with 2 mM GlutaMAX-I (Gibco) and 10% fetal bovine serum (FBS) (Cytiva HyClone™, Logan, UT). They were kept on ice to minimize cellular stress and degradation, then seeded and cultured for a few days in a-MEM supplemented with 2 mM GlutaMAX-I and 10% FBS.

Analysis of mitochondrial DNA content.

Mitochondrial DNA content was monitored via qRT-PCR. Mitochondrial DNA and genomic DNA were extracted using RIPA buffer from ex-vivo cultured control or mutant periosteal cells. Mitochondrial-specific primers targeting Cytochrome B, 16S rRNA, and Cytochrome c oxidase subunit 3 were employed to assess mitochondrial content (10). Normalization was achieved by amplifying the nuclear b2-microglobulin gene. The sequences of the primers utilized for qRT-PCR are provided in Supplemental Table 8.

Measurement of intracellular ATP.

Periosteal cells isolated from the hindlimbs were seeded into flat-bottom, 24-well plates (Corning Primaria™, catalog no. 353847) at a minimum density of 1 x 10^5 cells per well in 1 mL of a-minimal essential medium (a-MEM) supplemented with 2 mM GlutaMAX-I (Gibco) and 10% FBS (Cytiva). The medium was refreshed every other day. Cells were cultured at 37°C in a 5% CO_2 atmosphere for 7 days. ATP levels were quantified using the CellTiter-Glo Luminescent Cell Viability Assay (Promega, catalog no. G7570), adhering to the manufacturer's protocol. Luminescence was measured with an EnVision plate reader, ensuring that each condition was represented by 2 or 3 technical replicates.

Cell Viability Assay.

The number of viable and dead cells was determined by trypan blue exclusion assay. This assay involves staining dead cells with trypan blue dye, as viable cells exclude the dye while dead cells do not. Images for the cell viability assay were captured using a Nikon Eclipse TS100 microscope.

Single Cell RNA-Sequencing.

Periosteal cells were isolated from hindlimbs of *TFAM*$^{fl/fl}$ and *PRX;TFAM*$^{fl/fl}$ mice, as well as from *TFAM*$^{fl/fl}$*;HIF1dPA*$^{fl/fl}$ and *PRX;TFAM*$^{fl/fl}$*;HIF1dPA*$^{fl/fl}$ mice at p21 as previously described. Cells

were resuspended in phosphate-buffered saline (PBS) (Gibco) supplemented with 10% fetal bovine serum FBS (Cytiva) and subsequently filtered through a 40 µm nylon mesh (Flowmi™ Cell Strainer, MilliporeSigma) to clear tissue debris. The volume of the suspension was then adjusted to achieve the optimal cell concentration for sequencing.

Sequencing was performed at the CAG Sequencing Core at Children's Hospital of Philadelphia. Next-generation sequencing libraries were prepared using the 10x Genomics Chromium Single Cell 3' Reagent Kit v3 according to the manufacturer's instructions. Libraries were uniquely indexed using the Chromium Dual Index Kit, pooled, and sequenced on an Illumina NovaSeq 6000 sequencer using a paired-end, dual-indexing run strategy. Sequencing for each library aimed for 20,000 mean reads per cell. The data were then processed using the Cell Ranger pipeline (10x Genomics, version 6.1.2) for demultiplexing, alignment of sequencing reads to the mm10 mouse transcriptome, and generation of feature-barcode matrices (11). Each genotype was processed in biological duplicates.

Seurat Analysis.

ScRNA-Seq data were analyzed using the Seurat package (version 5.0.2) in R. Quality control started with individual assessments of duplicate samples to validate data integrity, followed by their consolidation into a unified dataset. SoupX was integrated to address ambient RNA contamination prior to advancing through the canonical Seurat processing pipeline (12). An initial clustering resolution of 0.8 was selected for both preprocessing and identifying potential doublets via DoubletFinder. This tool was utilized not only to flag doublets but also to investigate their nature, particularly the potential representation of multinucleated osteoclasts. Post-preprocessing, data integration, and clustering were conducted at varying resolutions (0.1, 0.2, and 0.8) to refine the clustering process and ensure robust cluster formation. A threshold was set to exclude cells exhibiting over 15% mitochondrial gene expression to mitigate biases from apoptotic or damaged cells. Further, cells with fewer than 200 detected features were filtered out

to enhance clustering precision. Differential expression analysis employed a Wilcoxon rank-sum test, setting significance at an adjusted p-value of less than 0.05 and requiring a log-fold change greater than 1.5. Visualization of key markers and co-expression patterns was achieved through feature plots, violin plots, and nebula plots, providing a multifaceted view of the gene expression data (13).

REFERENCES

1. Logan M, Martin J, Nagy A, Lobe C, Olsen E, and Tabin C. Expression of Cre recombinase in the developing mouse limb bud driven by a Prx1 enhancer. *Genesis.* 2002;33:77-80.
2. Larsson NG, Wang J, Wilhelmsson H, Oldfors A, Rustin P, Lewandoski M, et al. Mitochondrial transcription factor A is necessary for mtDNA maintenance and embryogenesis in mice. *Nat Genet.* 1998;18(3):231-6.
3. Kim JW, Tchernyshyov I, Semenza GL, and Dang CV. HIF-1-mediated expression of pyruvate dehydrogenase kinase: a metabolic switch required for cellular adaptation to hypoxia. *Cell metabolism.* 2006;3(3):177-85.
4. Madisen L, Zwingman TA, Sunkin SM, Oh SW, Zariwala HA, Gu H, et al. A robust and high-throughput Cre reporting and characterization system for the whole mouse brain. *Nat Neurosci.* 2010;13(1):133-40.
5. Nakada D, Saunders TL, and Morrison SJ. Lkb1 regulates cell cycle and energy metabolism in haematopoietic stem cells. *Nature.* 2010;468(7324):653-8.
6. Mangiavini L, Merceron C, and Schipani E. Analysis of Mouse Growth Plate Development. *Curr Protoc Mouse Biol.* 2016;6(1):67-130.
7. Schindelin J, Arganda-Carreras I, Frise E, Kaynig V, Longair M, Pietzsch T, et al. Fiji: an open-source platform for biological-image analysis. *Nat Methods.* 2012;9(7):676-82.
8. Merceron C, Ranganathan K, Wang E, Tata Z, Makkapati S, Khan MP, et al. Hypoxia-inducible factor 2alpha is a negative regulator of osteoblastogenesis and bone mass accrual. *Bone Res.* 2019;7:7.
9. van Gastel N, Torrekens S, Roberts SJ, Moermans K, Schrooten J, Carmeliet P, et al. Engineering vascularized bone: osteogenic and proangiogenic potential of murine periosteal cells. *Stem Cells.* 2012;30(11):2460-71.
10. Yao Q, Khan MP, Merceron C, LaGory EL, Tata Z, Mangiavini L, et al. Suppressing Mitochondrial Respiration Is Critical for Hypoxia Tolerance in the Fetal Growth Plate. *Dev Cell.* 2019;49(5):748-63 e7.
11. Zheng GX, Terry JM, Belgrader P, Ryvkin P, Bent ZW, Wilson R, et al. Massively parallel digital transcriptional profiling of single cells. *Nat Commun.* 2017;8:14049.
12. Young MD, and Behjati S. SoupX removes ambient RNA contamination from droplet-based single-cell RNA sequencing data. *Gigascience.* 2020;9(12).
13. Hao Y, Stuart T, Kowalski MH, Choudhary S, Hoffman P, Hartman A, et al. Dictionary learning for integrative, multimodal and scalable single-cell analysis. *Nat Biotechnol.* 2024;42(2):293-304.

Table S1: TFAM Cortical Bone Histomorphometry Data. Values represent the mean ± SD of at least 5 animals per group unless otherwise stated. An asterisk (*) indicates a significant difference between groups.

Histomorphometric Data	CTRL	TFAM	CTRL *vs* TFAM
	Mean±SD	*Mean±SD*	*p-value*<br>*CI*
Cortical Thickness (mm)	0.08±0.007	0.038±0.024	**0.0027***<br>(-0.065 to -0.019)
Cortical Bone Surface/Bone Volume (1/mm)	20.52±2.319	29.37±3.513	**0.0007***<br>(4.863 to 12.84)
Cortical Bone Mineral Density (gHA/cm^3)	0.750±0.063	0.469±0.101	**0.0003***<br>(-0.393 to -0.168)
Periosteal Osteoblast-like Cell Number	13.25±12.96	36.50±20.54	**0.0231***<br>(3.781 to 42.72)
Periosteal Osteoblast-like Cell Number/Bone Surface (1/mm)	2.468±3.027	13.08±6.374	**0.0013***<br>(5.059 to 16.17)
Periosteal Osteoclast Number	7.375±12.53	49.17±18.63	**0.0003***<br>(23.71 to 59.88)
Periosteal Osteoclast Number/Bone Surface (1/mm)	1.985±2.799	18.30±7.305	**0.0002***<br>(9.77 to 22.85)
Periosteal Perimeter (mm)	4.169±0.631	2.743±0.315	**0.0004***<br>(-2.054 to -0.798)
Periosteal Single Labelled Perimeter (mm)	0.321±0.556	0.339±0.276	0.9505<br>(-0.603 to 0.638)
Periosteal Double Labelled Perimeter (mm)	1.318±0.654	0.197±0.25	**0.0058***<br>(-1.826 to -0.414)
Periosteal Osteoid Surface (mm)	1.586±0.95	0.125±0.107	**0.0091***<br>(-2.448 to -0.474)
Periosteal Osteoid Surface/Bone Surface (%)	15.62±6.056	2.299±1.497	**0.0014***<br>(-19.76 to -6.89)
Periosteal Osteoid Volume/Bone Volume (%)	9.973±2.3	14.19±13.41	0.508<br>(-9.818 to 18.25)
Periosteal Osteoid Width (mm)	3.723±1.305	3.024±0.697	0.321<br>(-2.225 to 0.82)
Periosteal Mineral Apposition Rate (μM/Day)	2.31±1.044	0.783±0.884	**0.0296***<br>(-2.865 to -0.1895)
Periosteal Mineralizing Surface/Bone Surface	0.876±0.227	0.254±0.192	**0.0009***<br>(-0.913 to -0.331)
Periosteal Bone Formation Rate/Bone Surface (μM/Day)	2.191±1.296	0.275±0.284	**0.0106***<br>(-3.263 to -0.566)
Endosteal Osteoblast-like Number	68.43±31.35	64.17±32.79	0.8153<br>(-43.46 to 34.94)
Endosteal Osteoblast-like Number/Bone Surface (1/mm)	25.13±11.29	37.42±19.22	0.1796<br>(-6.582 to 31.15)
Endosteal Osteoclast Number	42.43±23.61	54±25	0.4095<br>(-18.13 to 41.27)
Endosteal Osteoclast Number/Bone Surface (1/mm)	15.59±8.655	31.57±15.54	**0.0392***<br>(0.95 to 31.01)
Endosteal Perimeter (mm)	2.725±0.133	1.731±0.169	**<0.0001***<br>(-1.169 to -0.8181)
Endosteal Single Labelled Perimeter (mm)	0.375±0.216	0.289±0.35	0.6269<br>(-0.474 to 0.302)
Endosteal Double Labelled Perimeter (mm)	0.365±0.45	0.088±0.148	0.2235<br>(-0.756 to 0.202)
Endosteal Osteoid Surface (mm)	0.924±0.685	0.393±0.269	0.1456<br>(-1.291 to 0.228)

Endosteal Osteoid Surface/Bone Surface (%)	8.058±3.376	5.135±2.282	0.1473<br>(-7.126 to 1.279)
Endosteal Osteoid Volume/Bone Volume (%)	2.616±1.045	5.31±2.71	0.0718<br>(-0.301 to 5.69)
Endosteal Osteoid Width (mm)	6.1±2.467	2.429±0.722	**0.0128***<br>(-6.322 to -1.020)
Endosteal Mineral Apposition Rate (μM/Day)	1.041±0.45	0.488±0.7	0.1471<br>(-1.34 to 0.235)
Endosteal Mineralizing Surface/Bone Surface	0.453±0.29	0.245±0.2896	0.2660<br>(-0.606 to 0.189)
Endosteal Bone Formation Rate/Bone Surface (μM/Day)	0.568±0.655	0.274±0.396	0.4046<br>(-1.055 to 0.466)
Osteocyte Number	351.6±48.63	223.7±49.01	**0.0006***<br>(-187.7 to -68.14)
Osteocyte Number/Bone Surface (1/mm)	66.27±12.62	92.76±14.01	**0.0064***<br>(9.277 to 43.71)

Table S2: Distribution of cellular populations across identified clusters in Single-Cell RNA Sequencing Analysis. Percentages in bold represent average values across biological replicates, percentages in parentheses represent individual sample values.

Genotype	Cluster Number	Cell Type	Cell Percentage CTRL	Cell Percentage Mutant
**PRX;TFAM**	1	Chondrocyte-like Cells	**33.37%** (30.97% - 35.77%)	**23.61%** (34.47 - 12.75%)
	2	Mesenchymal Progenitor-like Cells	**14.79%** (15.03% – 14.55%)	**40.66%** (36.56% - 44.76%)
	3	Osteoblast-like Cells	**20.90%** (23.82% - 17.99%)	**10.35%** (9.21% - 11.50%)
	4	Smooth Muscle-like Cells	**14.00%** (15.03% - 12.97%)	**11.50%** (8.54% - 14.46%)
	5	Proliferative Cells	**5.77%** (5.10% - 6.44%)	**4.89%** (5.06% - 4.72%)
	6	Other Cells	**6.51%** (6.04% - 6.99%)	**3.81%** (2.56% - 5.07%)
	7	Other Cells	**2.61%** (2.25% - 2.97%)	**4.80%** (3.16% - 6.43%)
	8	Pre-Hypertrophic/Hypertrophic-like Cells	**2.04%** (1.76% - 2.32%)	**0.38%** (0.44% - 0.32%)
**PRX;TFAM;HIF1dPA**	1	Chondrocyte-like Cells	**45.29%** (40.39% - 50.19%)	**65.96%** (63.72% - 68.21%)
	2	Osteoblast-like Cells	**29.47%** (29.21% - 29.72%)	**16.79%** (17.72% - 15.86%)
	3	Pre-Hypertrophic/Hypertrophic-like Cells	**6.25%** (5.10% - 7.40%)	**9.94%** (9.75% - 10.14%)
	4	Mesenchymal Progenitor-like Cells	**9.82%** (13.78% - 5.86%)	**3.66%** (4.57% - 2.76%)
	5	Smooth Muscle-like Cells	**5.77%** (8.34% - 3.21%)	**3.25%** (3.95% - 2.55%)
	6	Other Cells	**3.40%** (3.18% - 3.63%)	**0.39%** (0.29% - 0.48%)

Table S3: List of differentially expressed genes identified in scRNA-Sequencing comparing CTRL and TFAM Group. Included genes meet the criteria of having a fold change (FC) ≥ 1.5 and a p-value ≤ 0.05.

Control Upregulated Genes

Gene Symbol	Cluster 1 FC	Cluster 2 FC	Cluster 3 FC	Cluster 4 FC	Cluster 5 FC	Cluster 6 FC	Cluster 7 FC	Cluster 8 FC
Abcb1a							2.10	
Acan	1.57				2.24			
Adamts15				1.51				
Alpl					1.73			
Angptl1		1.56						
Anxa2		1.58	1.50					
Aoc3				1.51				
Apcdd1							1.76	
Arhgap29				1.53				
Atp1a2				1.65				
Bcam				1.56				
Bglap			2.42					
Bglap2			2.27					
Bgn	1.63							
Bmp1		1.50						
Bsg								2.14
C130074G19Rik							1.55	
C1qtnf3								2.60
Calm1		1.58						
Calr		1.69	1.63					1.83
Carmn				1.56				
Cavin3				1.64				
Ccn1	2.52			2.35	2.12			
Ccn2	2.34			1.55	1.89	1.81	1.93	

Ccn3		1.96	2.20					
Cd248		1.59						
Cd34		1.51						
Cd55		1.53						
Cenpf					1.52			
Cfb		1.57						
Chad	1.95	3.37	2.11		2.49			
Chst11	1.50							
Chst3	1.68							
Cilp	2.22	1.69						
Cilp2	1.89	1.94			1.99			
Clec11a		1.93	1.68		1.86			1.82
Clec3b		1.76						
Clic5						1.79		
Clu		1.93	1.54					
Cnmd	1.51				2.18			
Col11a1	1.77	2.11		1.54	2.75	1.81	2.16	
Col11a2	2.40	2.06			3.15			
Col12a1		1.71			1.69		1.67	
Col15a1	1.84	1.78						
Col16a1			1.94		2.10			
Col1a1	1.65	2.00	1.60			2.62		
Col1a2		1.63				2.00		
Col27a1					1.72			
Col2a1	2.91	2.97		4.65	4.24			
Col3a1	1.51		1.59			1.60		
Col5a2	1.85							
Col6a2				1.52				
Col8a1		1.52	2.53					

Col8a2							1.51	
Col9a1	1.87				2.79			
Col9a2	1.66				2.27			
Col9a3	2.34				3.61			
Comp	1.59	2.42			2.33	1.93		
Cox4i2	1.77				1.88			2.28
Cp						1.74		
Cpe					1.56			
Cpxm2							4.39	
Cpz			1.66					
Crabp1			1.83					
Crip1	1.75	2.72		1.72	1.56		1.78	
Crip2		1.69						
Crispld1					1.61			
Cst3						1.90		
Cthrc1		1.54	1.52					
Ctsh						1.55		
Cxcl12				1.66		2.14		
Cxcl14	1.58	2.13	2.58		1.50			
Dbi							1.52	
Dcn			1.55			2.20		
Dlk1				2.05				
Drp2							1.51	
Dusp1	1.92							
Ecm1		1.66						
Ecm2		1.52						
Ecrg4	1.59	2.83	2.13		2.55	2.46	2.92	
Efemp2								1.67
Eln			2.75					

Emp3		1.78						
Eny2								1.68
F5								1.74
Fam162a	1.78							1.86
Fam180a					1.63		1.55	
Fam3c							1.51	
Fap	1.51				1.57			
Fbln5						1.53		
Fbln7						1.74		
Fbn1		1.74	1.84					
Fbxl22				1.53				
Fdps					1.53			
Fibin	1.55				1.71			
Fkbp11			1.53					2.73
Fkbp7			1.65					
Fmod	1.52	1.75			2.06	1.93	2.35	
Fos	1.53			1.93				
Fst				2.08				
Fstl1		1.60		1.52				
Fxyd3	1.52							
Fxyd5		1.57						
Fzd4				1.57				
Gchfr						1.72		
Gdf10	1.62							
Gja1			1.67					
Gm42418				1.62		1.57		
Gng11		1.61					1.95	
Gpha2			2.33					
Gpx3						2.61		

Grb14								1.91
Grip2				1.51				
Gucy1a1				1.57				
H2afv					1.55			
Hapln1	1.69				2.58			
Hes1				2.12				
Hhip	1.63				1.62			
Higd1a								2.20
Hmgb2					1.56			
Hmgcs1					1.58			
Hsp90b1		1.50	1.53					
Hspa1a				2.24				
Hspa1b				2.25				
Htra3	1.58		1.51					
Htra4						1.79		
Id1							2.08	
Id2	1.76				1.52		1.92	
Igfbp2							1.60	
Igfbp3			1.66					
Igfbp4						1.76		
Igfbp5			1.91	1.87		1.53	1.84	
Igfbp6		2.05						
Igfbp7			1.58					
Islr		1.68						
Itgb5							1.74	
Itih5				1.58			4.91	
Itm2a			1.55		1.67			
Jun				1.70				
Kctd12							1.60	

Kera		2.45						
Lbp		2.14	2.07					
Ldha								2.18
Lgals1							1.67	
Loxl1						1.56		
Loxl2	1.61							
Loxl3	1.70							
Ltbp4		1.52						
Lum			1.63					
Luzp2							2.06	
Ly6a		1.53				1.92		
Ly6c1		1.71				2.07		
M1ap								1.50
Maged2		1.78		1.72				
Manf		1.54	1.54					1.92
Matn1								4.27
Matn2		1.52						
Matn3					2.04			
Matn4			1.68			1.62		
Mdk			1.56					
Meg3					1.65			
Meox2		1.53		1.62			1.70	
Mfap4			1.67					
Mfge8					3.17			2.39
Mgp				1.71		2.22		
Mia	1.97				2.70			
Mif	1.61							
Mob2				1.64				
Myadm		1.75						

Myl9				1.52				
Ndufa4l2		1.96	1.57		2.66			3.25
Nme1								1.76
Notum							1.77	
Npy1r				1.66				
Nr1d1	1.51							
Nrep		1.61		1.65				
Nrip2				1.91				
Ntrk2				1.51				
Olfml2a				1.81				
Olfr558				2.30				
Ooep								1.62
Ostc		1.51	1.57					
Ostn			1.89					
P4ha1	1.61	1.60			1.54			1.97
P4ha2	1.67							
Pcolce		1.70	1.91				1.67	
Pcolce2							1.56	
Pcp4l1				1.77				
Pcsk6						1.69		
Pdgfrl			1.53		1.63		1.67	
Pgam1								2.12
Phlda1	1.52							
Pla1a						1.91		
Plat			1.50					
Pln				2.18				
Plod2	1.89				2.03			
Postn			1.79					
Ppp1r12b				1.62				

Prelp	1.69	1.64						
Prg4		2.37	1.80		2.24			
Prrx1				1.53				
Ptn		1.61	2.00				2.48	
Ptp4a3				2.00				
Ptprd							1.51	
Rarb							1.59	
Rarres2							2.48	
Rasl11a				1.78				
Rbp1							2.12	
Rcan2				1.84				
Rcn3	2.03	1.81	1.83		1.99	1.59	1.96	2.00
Rflnb		1.54						
Rgs4				2.30				
Rgs5				1.69				
Rian					1.60			
Rora	1.67							
Rspo3							1.72	
S100a10		1.79	1.52					
S100a11		1.59	1.56				1.61	
S100a13		1.55						
S100a4		1.82	1.69			2.15		
S100a6		1.56	1.52					
Sbsn		1.50						
Scrg1	1.57				1.95			
Scx		1.83						
Sec11c								1.51
Sec61b								2.11
Sec61g								1.95

Selenom		1.55	1.54					1.86
Serf1			1.50					
Serf2			1.54					1.75
Serp1								1.78
Serpine2				1.57				
Serpinf1			1.70					
Serping1						1.64		
Serpinh1		2.01	1.79		1.94		1.64	2.06
Sfrp4			1.55					
Slc16a3								1.55
Slc20a1	1.58							
Smpd3	1.59				1.56			
Snorc	1.63				2.71			
Sorbs2				2.21				
Sox9					2.07			
Sparc	1.67	1.64	1.52		1.92		1.79	
Spon2		2.32	2.23				1.57	
Srgn						1.91		
Ssr1								1.84
Steap4				1.55				
Stmn1					1.81			
Stra6							2.05	
Sulf2	1.77							
Susd5					1.65			
Tbx2				1.51				
Tcf15				1.69				
Tent5a	1.63							
Tesc				2.13				
Thbs4	1.75	1.94						

Thsd4							2.32	
Thy1		1.59						
Timp3						1.52		
Tinagl1				1.57				
Tmem140							1.51	
Tmsb10			1.81				1.65	
Tnfrsf11b	2.13				1.58			
Tnmd		2.10						
Tnn					1.64			
Tnxb		1.53						
Tomm7								1.65
Top2a					1.53			
Tpi1								1.99
Tppp3		1.83		1.57				
Tuba1a		1.76	1.85	1.51		1.77	1.69	
Txnip				1.65		2.52		
Uba2				1.59				
Vcan		1.78						
Vit	1.81				1.79			
Vkorc1	1.58		1.51					
Wif1	2.31	1.83	1.88		2.68			
Wnt5a							1.63	
Wwp2	1.97				2.11			
Xist			2.24	2.25	1.85	2.28	1.75	

Mutant Upregulated Genes

Gene Symbol	Cluster 1 FC	Cluster 2 FC	Cluster 3 FC	Cluster 4 FC	Cluster 5 FC	Cluster 6 FC	Cluster 7 FC	Cluster 8 FC
1110038B12Rik		1.51						
2410006H16Rik		2.10	2.15	2.08	1.70		1.65	
Abi3bp			1.52					

Actg1						1.67	
Actg2				1.63			
Adamts1		2.01		2.69	2.06		2.52
Adamts4				4.33			1.76
Adamts9				2.00			
Aebp1	1.59	1.88		1.64	1.57		
Akap12				2.08			
Akap13							1.63
Aplp2			1.55				
Apod			2.07				
Apoe				1.75			
Apold1				2.38			
App				1.68			
Arl4a							1.85
Asns				1.69		1.56	
Asprv1					2.24		
Atf3		4.41	1.86			3.18	1.69
Atf4				1.51			1.64
Atf5			1.55	4.01	1.55	2.01	2.77
B3gnt2							1.84
Bach1				1.69			
Bag3		1.56		1.62			
Bcl3				1.55			1.69
Bhlhe40		1.50					
Brd2				1.76			1.64
Btg1		1.52				1.59	
Btg2		3.11	2.54			2.04	
Ccl2		2.57		3.30	2.57		2.74
Ccl7		2.47		1.72	1.75		2.69

Ccnd2				1.59			
Ccnl1		1.97				1.56	
Cd44				1.61	1.50		1.72
Cdkn1a	1.75	2.00	1.83	3.26	1.76	2.35	2.54
Cdkn1c	1.74						
Cebpb	2.11	3.60	1.95	1.99	1.93	2.88	2.41
Cebpd		2.77	2.83		1.77	1.57	2.01
Chchd10		3.39		4.73	1.63	2.25	3.58
Chil1	2.25						
Clic4							2.25
Coq10b		1.59					
Cox4i1	1.63						
Cox6a2				1.73			
Cox7a2l	1.60	1.61	1.52				
Cp							1.73
Crabp2							2.07
Crem				1.56			
Crispld2				2.19			
Cryab	2.80						
Csrnp1		1.64		1.67		1.59	
Cstb				1.94			1.88
Cstdc4					3.11		
Cstdc5					5.85		
Ctsl	1.56			1.85	1.67		
Cxcl1		4.20		3.23	3.36	2.38	1.93
Cxcl12					1.56		
Cxcl2		1.81		2.33	2.34		
Cyb5r1				1.55			
Ddit3			1.67				

Ddit4				1.73			
Ddx3x							1.60
Ddx3y				1.67			
Dlk1		1.58					
Dnaja1	1.74		1.58	1.61			1.60
Dnajb1		1.83		1.54			
Dot1l				1.65			1.51
Dusp1		2.11				2.52	
Dusp5							1.86
Ednrb				2.44			
Eef1a1		1.61		1.70			
Eef1b2		1.71		1.81			
Eef2		1.83	1.69	1.65			
Egr1		2.57	2.31			2.34	
Eif1				1.52			
Eif3c			1.53	1.54	1.62		
Eif3f	1.54	1.56	1.50				
Eif4ebp1					1.60		
Eif5				1.58			1.51
Eif5a							1.59
Entpd2						1.54	
Eprs				2.05			
Erf							1.58
Errfi1		2.00	1.78			1.76	
Esd					1.51		
F3							1.81
Fabp4				1.59			
Fabp5				1.84			
Fau	1.67	1.78	1.72	1.75	1.58		

Fbln2					2.38	1.93	
Fgl2		1.95					
Fkbp5							1.68
Flnc				1.66			
Fmo2		1.51					
Fn1				1.62			
Fos		2.18	1.62			2.32	
Fosb		1.74	1.69				
Fosl2		1.84		1.60			2.07
Fst		1.65					
Fth1	2.11	1.69		1.58	1.78	1.55	1.77
Ftl1	1.69	1.71		1.73	1.71		1.61
Gadd45a		1.57	1.66				
Gadd45b		2.25	1.89				
Gadd45g		2.78				1.69	
Gas5		2.10	2.10	2.12			1.55
Gclc				1.75			
Gdf15				1.55		1.53	
Gem		2.10			1.73		1.85
Glul						1.55	
Gm10076				1.53			
Gm10260	1.69	1.58			1.64		
Gm13889				1.82		1.84	
Gm26532			2.47	1.67			1.58
Gpc1			1.59				
Gpnmb		2.06			1.62		
Gprc5a						1.61	
Gpx3	1.79						
Has1		3.24			2.11	2.84	2.06

Hbegf						1.85	
Hes1		1.56					
Hilpda				1.72			1.68
Hipk1							1.62
Hk2		2.04		1.68			1.53
Hmox1		1.57					1.71
Hsp90aa1	1.95	1.97	1.63				
Hsp90ab1	1.56				1.56		
Hspa1a	1.60						
Hspa8		1.58				1.66	
Hspa9		1.61	1.64	2.10	1.67	1.51	1.80
Hspb1						2.03	
Hsph1							1.61
Ibsp	2.07						
Icam1		1.66					
Id3		1.88					
Ier2		1.78	1.62			1.70	
Ier3		3.44	2.63	2.23	1.84	2.49	
Ier5		1.61		1.53		1.83	1.63
Ifitm3	1.75						
Ifrd1		2.29	1.64	2.02		1.73	2.41
Igf2	1.64	1.59					
Igfbp2				2.39			
Il1b					1.76		
Il6		1.93		2.77	1.62		
Impdh2				1.78			
Inhba						2.43	
Irf1		1.93					1.54
Irs2		1.59		1.74			

Isg15				1.51			
Itga5						1.51	
Itga6							1.79
Jak1							1.55
Jun		2.15					
Junb		3.77	1.75			3.08	
Jund		1.91	1.74			1.70	
Kdm6b				1.62			1.84
Klf2		1.65	2.16			2.38	
Klf4		2.36	2.71	3.11		2.60	1.56
Klf6							1.66
Klf9		1.63	1.53				2.08
Lgals3			1.54	1.61	1.54		
Litaf							1.69
Lmna				1.60			1.99
Mafb	1.72						
Mat2a				1.72		1.58	1.79
Mcl1			1.57	1.67	1.60		1.68
Mfap5					1.68		
Midn	1.51			1.66			1.57
Mif				1.82			
Msmp	2.85						
Mt1	2.36	4.56	3.34	3.62	3.54	3.95	3.66
Mt2	1.76	3.14	1.77	3.56	2.82	4.64	3.35
Mthfd2				1.71			
Myc		1.76					
Myd88							1.58
Neat1		1.75	1.63	2.46			2.47
Nfe2l2				1.67			1.79

Nfil3		1.53					
Nfkb1				1.85			
Nfkbia		2.61	1.53	2.40	1.99	2.20	
Nfkbiz		1.76		1.59			
Npm1		1.59		1.63			
Nr4a1		2.38	1.78			1.80	1.88
Nr4a2		1.70		1.85			1.83
Nr4a3		1.52		2.36			2.06
Nrp2	1.51						
Nsa2	1.61	1.71	1.62	1.74			
Nupr1				1.97			
Odc1				1.62			
Pabpc1		1.64		1.77	1.53	1.56	
Pcdh19							1.53
Peli1							1.66
Penk	1.69		1.69				
Phlda1		1.66				2.26	
Pim1		1.59					1.56
Pla2g2e						1.52	
Plec							1.63
Plpp3					1.54		
Plscr1							1.60
Pnp		1.73					
Pnrc1		1.62					
Ppp1r15a		1.92					
Pqlc1				1.79			
Procr				2.25			
Ptgs2		1.87					
Ptx3		3.58			2.19		

Pvr							1.51	
Rack1	1.62	1.89	1.69	2.05	1.66	1.51		
Rbbp6							1.61	
Rbm3	1.62							
Rbm39								1.60
Rcan1		1.64				2.84		
Rgcc			1.83			1.68		
Rgs16			1.62				1.65	
Rgs2			1.61					
Rnf19b			1.62					
Rpl10	1.58	1.58	1.50	1.70	1.58			
Rpl10a		1.56		1.58				
Rpl12	1.83	2.33	2.03	2.63	2.06	1.67	1.55	
Rpl13	1.65	1.72	1.50	1.72	1.56			
Rpl13a	1.57	1.75	1.71	1.96	1.62			
Rpl14	1.61	1.80	1.58	1.67	1.61			
Rpl15		1.68		1.71				
Rpl17	1.73	1.93	1.81	1.93	1.74	1.55		
Rpl18	1.62	1.77	1.56	1.77	1.56			
Rpl18a	1.58	1.90	1.63	1.79	1.56			
Rpl19		1.67		1.68				
Rpl21	1.52	1.60	1.52	1.71				
Rpl22				1.56				
Rpl23	1.72	1.96	1.73	2.10	1.65	1.61		
Rpl23a		1.54	1.55	1.66				
Rpl24	1.64	1.71	1.54	1.73	1.55			
Rpl26	1.51	1.73	1.55	1.77				
Rpl27a		1.67	1.51	1.61				
Rpl28	1.50	1.59		1.60				

Rpl29	1.62	1.68		1.57	1.51	
Rpl3		1.59		1.51		
Rpl30	1.60	1.69	1.63	1.83	1.60	
Rpl32	1.68	1.95	1.62	1.93	1.69	
Rpl34		1.63	1.52	1.62		
Rpl35		1.50		1.60		
Rpl35a		1.55		1.53		
Rpl36		1.67		1.56		
Rpl36a		1.59	1.55	1.73		
Rpl37		1.54	1.51	1.52		
Rpl37a		1.52				
Rpl38				1.57		
Rpl39		1.64		1.85		
Rpl4	1.50	1.76	1.56	1.80	1.50	
Rpl5	1.73	2.08	1.96	2.07	1.75	1.65
Rpl6		1.64		1.62		
Rpl7		1.57		1.61		
Rpl8	1.63	1.59		1.61		
Rpl9	1.53	1.62		1.53		
Rplp0		1.79		1.89		
Rplp1		1.81	1.52	1.95		1.52
Rplp2	1.58	1.70	1.53	1.74		
Rps10	1.67	1.78	1.56	1.74	1.58	
Rps11		1.55		1.65		
Rps12		2.01	1.72	2.20	1.64	1.61
Rps13	1.66	1.72	1.55	1.76	1.54	
Rps14	1.75	1.88	1.72	1.77	1.67	
Rps15	1.56	1.81	1.71	1.95	1.58	1.59
Rps15a		1.81	1.58	1.83	1.56	

Rps16	1.71	1.89	1.62	1.75	1.55		
Rps17		1.50					
Rps18	1.56	1.79	1.57	1.74	1.57		
Rps19	1.77	1.99	1.72	2.06	1.55	1.53	
Rps2		1.75	1.53	1.97	1.60		
Rps20		1.81	1.53	2.08	1.52		
Rps21		1.60		1.62			
Rps23	1.61	1.77	1.61	1.84	1.62		
Rps24	1.50	1.91	1.59	2.18		1.54	
Rps25	1.60	1.80	1.67	1.71	1.54		
Rps26		1.63	1.53	1.73			
Rps27	1.53	1.64	1.63	1.74			
Rps27a	1.52	1.61		1.63			
Rps28		1.74	1.57	1.84			
Rps3		1.79		1.69			
Rps3a1	1.50	1.69		1.81			
Rps4x	1.59	1.83	1.53	1.77	1.56		
Rps5	1.75	2.08	1.77	2.07	1.67		
Rps6	1.67	1.94	1.63	1.90	1.66	1.51	
Rps7	1.62	1.89	1.63	1.96	1.56		
Rps8	1.61	1.80	1.64	1.93			
Rps9		1.56					
Rpsa		1.65		1.84			
Rrad		1.76	1.60				
Sars				1.64			
Sash1				1.59			
Sat1		2.16	1.57			2.00	2.52
Sbno2		1.93		1.84	1.51		1.50
Scara3	1.51						

Sdc4				1.72			
Serpine1		1.50	1.78			2.48	
Slc38a2		1.68	1.54	1.55			
Slc3a2		1.57		1.89		1.57	1.72
Slc6a17				1.93			
Slc6a6							1.66
Slc7a1							1.53
Slpi					1.65		
Smarca5				1.79			
Sned1	1.56						
Snhg12		1.54					
Snhg6		1.56	1.79	1.51			
Snhg8		1.68	1.62				
Socs1							1.57
Socs3		2.37				2.04	
Spp1	1.95		3.74				
Sqstm1		1.58		1.60	1.55		
Stat3							1.51
Stfa1					2.83		
Stfa2					4.46		
Stfa2l1					3.38		
Stfa3					2.47		
Tenm2							1.54
Tgfb2	1.61						
Tgif1				1.66			
Tgm2						1.54	
Thbd						1.91	
Thbs1				1.61			
Thbs4			1.57				

Timp2	1.58						
Tiparp		2.23		2.16		1.63	2.46
Tm4sf1	1.63						
Tmbim1				1.54			1.60
Tnc				1.64			
Tnfaip2				2.38			
Tnfaip6		3.06	1.63	1.55	2.38	3.29	
Tnfsf8							1.50
Top1							1.65
Tpt1	1.61	1.88	1.66	2.08	1.69	1.58	1.65
Tsc22d3			2.15				
Uap1				2.12			1.65
Uba52		1.76	1.70	2.00		1.50	
Ubb		1.75		1.52		1.53	
Ubc		2.35	1.63			2.25	
Ucp2						1.59	
Ugdh		1.65		2.57		1.85	3.20
Vat1				1.63			
Vim					1.64		
Ywhag							1.51
Zbtb16							1.51
Zfas1			1.59				
Zfp36		3.52	1.80			2.58	
Zfp36l1		1.86	1.92		1.67	1.66	1.99
Zfp703				1.62			

Table S4: PRX;HIF1dPA Cortical Bone Histomorphometry Data. Values represent the mean ± SD of at least 5 animals per group unless otherwise stated.

Histomorphometric Data	CTRL	HIF1dPA	CTRL *vs* HIF1dPA
	Mean±SD	*Mean±SD*	*p-value*<br>*CI*
Cortical Thickness (mm)	0.093±0.031	0.088±0.018	0.757<br>(-0.040 to 0.030)
Cortical Bone Surface/Bone Volume (1/mm)	21.60±5.672	17.92±2.758	0.2282<br>(-10.19 to 2.824)
Cortical Bone Mineral Density (gHA/cm^3)	0.85±0.146	0.923±0.0713	0.3420<br>(-0.09 to 0.2358)
Periosteal Osteoblast-like Cell Number	52.33±26.70	45±24.49	0.649<br>(-42.59 to 27.93)
Periosteal Osteoblast-like Cell Number/Bone Surface (1/mm)	12.76±6.525	10.92±5.805	0.6362<br>(-10.36 to 6.672)
Periosteal Osteoclast Number	6.8±4.658	7.8±5.975	0.775<br>(-6.813 to 8.813)
Periosteal Osteoclast Number/Bone Surface (1/mm)	3.953±5.848	1.884±1.424	0.463<br>(-8.18 to 4.042)
Periosteal Perimeter (mm)	4.089±0.280	4.120±0.426	0.8887<br>(-0.452 to 0.514)
Periosteal Single Labelled Perimeter (mm)	0.351±0.632	0.346±0.381	0.9866<br>(-0.676 to 0.666)
Periosteal Double Labelled Perimeter (mm)	0.945±0.836	1.117±0.514	0.6768<br>(-0.721 to 1.065)
Periosteal Osteoid Surface (mm)	0.776±0.493	0.993±0.495	0.5075<br>(-0.504 to 0.938)
Periosteal Osteoid Surface/Bone Surface (%)	6.244±3.982	15.35±9.135	0.0752<br>(-1.167 to 19.39)
Periosteal Osteoid Volume/Bone Volume (%)	1379±8.865	11.40±5.492	0.6134<br>(-12.74 to 7.954)
Periosteal Osteoid Width (mm)	3.697±1.153	4.604±1.865	0.382<br>(-1.354 to 3.168)
Periosteal Mineral Apposition Rate (μM/Day)	1.192±0.854	1.208±0.702	0.9732<br>(-0.99 to 1.022)
Periosteal Mineralizing Surface/Bone Surface	0.6494±0.4178	0.803±0.183	0.427<br>(-0.2607 to 0.569)
Periosteal Bone Formation Rate/Bone Surface (μM/Day)	0.925±0.824	1.042±0.793	0.8076<br>(-0.923 to 1.157)
Endosteal Osteoblast-like Number	143.8±28.74	120.6±14.45	0.136<br>(-55.41 to 8.94)
Endosteal Osteoblast-like Number/Bone Surface (1/mm)	55.68±9.532	47.60±3.74	0.1103<br>(-18.39 to 2.239)
Endosteal Osteoclast Number	30.50±27.28	46.40±34.40	0.413<br>(-26.08 to 57.88)
Endosteal Osteoclast Number/Bone Surface (1/mm)	11.85±10.11	19.05±16.03	0.386<br>(-10.71 to 25.11)
Endosteal Perimeter (mm)	2.582±0.25	2.532±0.215	0.7351<br>(-0.371 to 0.272)
Endosteal Single Labelled Perimeter (mm)	0.51±0.231	0.344±0.139	0.1635<br>(-0.411 to 0.079)
Endosteal Double Labelled Perimeter (mm)	0.54±0.164	0.364±0.268	0.2003<br>(-0.463 to 0.1103)
Endosteal Osteoid Surface (mm)	1.628±0.437	1.149±0.312	0.0713<br>(-1.009 to 0.051)

Endosteal Osteoid Surface/Bone Surface (%)	15.19±7.105	18.72±7.033	0.4323<br>(-6.168 to 13.21)
Endosteal Osteoid Volume/Bone Volume (%)	5.569±3.532	3.547±2.917	0.3341<br>(-6.505 to 2.46)
Endosteal Osteoid Width (mm)	6.057±2.309	6.367±1.61	0.806<br>(-2.469 to 3.089)
Endosteal Mineral Apposition Rate (μM/Day)	0.806±0.255	0.656±0.398	0.4548<br>(-0.58 to 0.28)
Endosteal Mineralizing Surface/Bone Surface	0.65±0.142	0.511±0.304	0.3358<br>(-0.444 to 0.167)
Endosteal Bone Formation Rate/Bone Surface (μM/Day)	0.519±0.205	0.366±0.223	0.2453<br>(-0.428 to 0.122)
Osteocyte Number	295.7±84.76	298.4±87.61	0.9593<br>(-115.1 to 120.6)
Osteocyte Number/Bone Surface (1/mm)	66.59±20.93	73.16±13.50	0.561<br>(-18.10 to 31.25)

Table S5: PRX;TFAM;HIF1dPA Cortical Bone Histomorphometry Data. Values represent the mean ± SD of at least 5 animals per group unless otherwise stated. An asterisk (*) indicates a significant difference between groups.

**Histomorphometric Data**	**CTRL**	**TFAM;HIF1dPA**	**CTRL *vs* TFAM;HIF1dPA**
	Mean±SD	*Mean±SD*	*p-value*<br>*CI*
Cortical Thickness (mm)	0.121±0.018	0.137±0.013	0.1145<br>(-0.065 to 0.038)
Cortical Bone Surface/Bone Volume (1/mm)	19.65±2.799	17.60±1.528	0.1804<br>(-5.22 to 1.139)
Cortical Bone Mineral Density (gHA/cm^3)	0.831±0.105	0.786±0.044	0.397<br>(-0.159 to 0.069)
Periosteal Osteoblast-like Cell Number	45.20±36.22	32.33±24.66	0.5015<br>(-54.44 to 28.71)
Periosteal Osteoblast-like Cell Number/Bone Surface (1/mm)	10.47±8.525	9.097±6.933	0.774<br>(-11.90 to 9.146)
Periosteal Osteoclast Number	6.8±8.526	31.50±32.07	0.1312<br>(-8.953 to 58.35)
Periosteal Osteoclast Number/Bone Surface (1/mm)	1.533±1.793	8.594±8.598	0.1070<br>(-1.86 to 15.98)
Periosteal Perimeter (mm)	4.268±0.355	3.748±0.508	0.0869<br>(-1.133 to 0.092)
Periosteal Single Labelled Perimeter (mm)	0.3078±0.237	0.193±0.211	0.4454<br>(-0.439 to 0.2105)
Periosteal Double Labelled Perimeter (mm)	1.053±0.474	0.111±0.106	**0.004***<br>(-1.498 to -0.385)
Periosteal Osteoid Surface (mm)	1.081±0.269	0.938±0.418	0.5107<br>(-0.613 to 0.328)
Periosteal Osteoid Surface/Bone Surface (%)	7.647±8.058	5.602±4.964	0.634<br>(-11.44 to 7.348)
Periosteal Osteoid Volume/Bone Volume (%)	9.973±2.3	14.19±13.41	0.508<br>(-9.818 to 18.25)
Periosteal Osteoid Width (mm)	4.55±2.365	3.68±0.869	0.458<br>(-3.412 to 1.671)
Periosteal Mineral Apposition Rate (μM/Day)	1.602±0.482	0.616±0.413	**0.0077***<br>(-1.639 to -0.333)
Periosteal Mineralizing Surface/Bone Surface	0.701±0.267	0.278±0.131	**0.017***<br>(-0.749 to -0.094)
Periosteal Bone Formation Rate/Bone Surface (μM/Day)	1.121±0.433	0.324±0.297	**0.0102***<br>(-1.355 to -0.239)
Endosteal Osteoblast-like Number	163±63.55	115.3±63.51	0.246<br>(-134.7 to 39.35)
Endosteal Osteoblast-like Number/Bone Surface (1/mm)	60.59±19.32	57.87±24.52	0.845<br>(-33.35 to 27.91)
Endosteal Osteoclast Number	24.6±18.65	16.50±25.45	0.569<br>(-39.16 to 22.96)
Endosteal Osteoclast Number/Bone Surface (1/mm)	9.375±7.642	8.573±11.23	0.849<br>(-14.14 to 12.54)
Endosteal Perimeter (mm)	2.65±0.216	2.090±0.733	0.136<br>(-1.335 to 0.213)
Endosteal Single Labelled Perimeter (mm)	0.387±0.174	0.302±0.274	0.539<br>(-0.387 to 0.217)
Endosteal Double Labelled Perimeter (mm)	0.506±0.376	0.453±0.317	0.8157<br>(-0.56 to 0.453)

Endosteal Osteoid Surface (mm)	1.35±0.396	0.871±0.305	0.077<br>(-1.021 to 0.065)
Endosteal Osteoid Surface/Bone Surface (%)	8.454±5.418	6.215±4.144	0.505<br>(-9.651 to 5.172)
Endosteal Osteoid Volume/Bone Volume (%)	3.698±3.228	2.074±1.639	0.385<br>(-5.706 to 2.457)
Endosteal Osteoid Width (mm)	7.319±1.975	3.537±0.386	**0.006***<br>(-6.133 to -1.431)
Endosteal Mineral Apposition Rate (μM/Day)	1.036±0.371	0.795±0.636	0.4407<br>(-0.91 to 0.434)
Endosteal Mineralizing Surface/Bone Surface	0.562±0.313	0.873±0.487	0.2232<br>(-0.227 to 0.85)
Endosteal Bone Formation Rate/Bone Surface (μM/Day)	0.665±0.599	0.921±1.030	0.6088<br>(-0.836 to 1.348)
Osteocyte Number	326±102.5	409.5±102	0.2105<br>(-56.58 to 223.6)
Osteocyte Number/Bone Surface (1/mm)	72.80±14.68	124.2±8383	0.2122<br>(-35.21 to 138.1)

Table S6: Upregulated Gene Profile in TFAM;HIF1dPA Control versus Mutant from scRNA-Seq Data. Included genes meet the criteria of having a fold change (FC) ≥ 2 and a p-value ≤ 0.05.

Control Upregulated Genes

Gene Symbol	Cluster 1 FC	Cluster 2 FC	Cluster 3 FC	Cluster 4 FC	Cluster 5 FC	Cluster 6 FC
Aak1	2.83	2.40		2.34		
Abca9		2.59				
Abcc9					3.66	
Abhd14b		2.52				
Acad10	2.13					
Acads		2.19				
Accs	2.02	2.05				
Ackr4		5.94				
Acod1	3.63					
Acot11	2.06	2.12				
Acox1	2.90					
Acp5		5.98	3.03			
Acta2		3.44	5.14			
Actn1		2.08	2.00			
Actr5	2.51					
Acvr1b	2.76					
Acvrl1	2.84					
Adam12	2.04	6.66				
Adam19		3.42	3.65	4.02		
Adam22		6.02			6.66	
Adam23	2.83					
Adam33				4.60		
Adamts10		2.61				
Adamts12	3.95		2.71	5.87	3.58	
Adamts14		2.03				
Adamts15		6.57				
Adamts17		4.25				
Adamts2	2.51	2.28	2.91			
Adamts3	2.50	3.37				
Adamts4		3.86				
Adamts5	3.83			2.43		
Adamts7		3.28				
Adamtsl1		6.74	4.07			
Adamtsl3				3.33		

Adap2					4.41
Adarb1		2.65			
Adcy5		3.50			
Adcy7		2.09			
Adcy9	2.46	2.01	5.11	3.38	4.23
Add3		2.14			
Adgra2	2.66	2.27			
Adgrb2		2.88			
Adgrf5		2.75			
Adh7	4.86				
Ado			2.17		
Adprhl2	2.03				
Aff1	2.46				
Aff3				2.15	
Agap3	2.44				
Agmo	5.42	2.49			
Ago4	2.81				
Akap10	2.12				
Akap17b		2.26			
Akap6					2.63
Akt3	2.55			2.59	
Aldh3a1		6.27			
Alkbh1	2.51		2.06		
Alpl		2.66			
Ambra1	2.72			2.54	
Amigo1		2.91			
Ammecr1		2.60			
Ammecr1l	3.82				
Amot		2.55	2.68		
Amotl2		2.23			
Angpt1	2.76	4.18		2.53	
Angptl2		2.95			
Angptl7		19.83			
Ank	2.78				
Ank2				2.19	
Ankrd16	2.21				
Ankrd17	2.11				
Ankrd29		3.26	3.43		
Ankrd37	2.13				
Ankrd44		2.17			

Ankrd9		2.07			
Anks1	2.09		2.06		
Ano6					2.08
Ano8		2.03			
Anpep		2.35		2.48	
Antxr1		3.09	2.95	2.56	2.40
Antxr2				2.32	
Anxa11	2.75				
Anxa11os	9.34				
Anxa6		2.36			
Aoc3		13.28			
Aopep	2.69		2.02	2.52	2.09
Ap4e1	2.23				
Ap4m1		2.09	2.42		
Apba1	2.26				
Apbb1	2.32	2.23			
Apbb2					2.17
Apcdd1		2.23			
Aplp1		7.26			
Apod	6.06	3.84			
Aqp1		2.29	3.88		
Ar		2.73			
Arfgap1	2.21	2.40		2.59	
Arfgap2	2.10				
Arfgef1	2.08				
Arfgef3	2.77				
Arhgap10				2.34	3.10
Arhgap15	2.22				
Arhgap20		5.30			
Arhgap23	2.12				
Arhgap24				2.45	4.12
Arhgap28		4.16	3.34		
Arhgap31	2.35	2.04	2.21		
Arhgap32	2.06	2.35	2.09	2.49	2.85
Arhgap35	2.04				
Arhgap42		2.75	2.76	2.12	2.14
Arhgap6				2.45	2.69
Arhgef10		2.29	2.22		
Arhgef11	2.00	2.16	2.15		
Arhgef12	2.02				

Arid3a	2.24	3.41			
Arid3b	3.20				
Arih1	2.40				
Arl4d		3.10			
Arl5a		2.40			
Armcx4		2.33			
Armh3	2.07			3.13	
Arnt	2.89				
Arrb1		2.14			
Arrdc3				3.33	
Arrdc4		2.67			
Asap1			2.46		2.57
Asap3	2.90	3.80	3.09	5.82	
Aspa		2.97			
Aspn	3.17	4.11	12.16		
Asxl1	4.36		2.53		2.58
Asxl2	2.15				
Asxl3	3.76	2.85			
Atf7	2.08				
Atg13	2.39				
Atg16l1	2.02				
Atg2a	2.47				
Atoh8	2.95				
Atp10a	2.28				
Atp13a3	2.01				
Atp1b1	2.99				
Atp8a1		2.08			
Atp8b1		2.87			
Atp9a		2.49			
Atp9b				2.08	
Atxn2	2.08				
Atxn3	2.03				
Atxn7l1	2.02				
Atxn7l2	2.95				
Aunip			2.15		
Avl9	2.00				
Avpr1a	4.96				
Axl	2.46				
B130024G19Rik					4.48
B3galt1					6.06

B3gnt9		2.02			
B4galnt1		2.20			
B4galt2		2.52			
BC029722			2.34		
Bach2	2.07				
Basp1		2.99	3.65		
Baz2a	2.55				
Bcar1	2.40				
Bcas3					2.43
Bckdhb			2.70		
Bcl2l1	3.13				
Bcl6	2.06				
Bcor	2.12				
Bdh1	5.27	3.61			
Best1		2.88			
Bet1l	2.16	2.10			
Bicc1		4.27	2.99	2.60	
Bicra	3.01	2.17	3.42		
Birc3	2.62				
Bmf	2.65				
Bmp1		2.46			
Bmp2k	2.00	3.25	2.97		
Bmp4	2.58				
Bmp5	5.09	3.08	2.26		6.28
Bmp6	2.51				
Bmpr1a	2.22		2.11		2.69
Bmpr2	2.71				
Bnc2	2.46	2.26	3.01		
Boc				2.79	
Bok			2.66		
Borcs6	2.04				
Brd4	2.07				
Brf1	2.38				
Brsk1	2.39	2.35			
Brwd3	2.03				
Bsdc1	2.55				
Btaf1	2.15				
Btbd7	2.07				
Btrc	2.06				
C130021I20Rik	6.92				

C130050O18Rik	3.31				
C1galt1			2.07		
C1qtnf1	2.45	3.12			
C1qtnf2		2.20		2.74	
C1qtnf3	4.68	5.56	2.35		
C1qtnf6		2.54			
C1rb		15.28			
C1s1		2.20			
C1s2		25.64			
C2		2.31			
C2cd4a	5.85				
C2cd4d	2.59				
C2cd5		2.30			
Cachd1		3.19			
Cacna1c		3.13			2.54
Cacna1d	2.07	2.55			
Cacna1e	4.23				
Cacna1g	4.21	3.13			
Cacna2d3		6.12			
Cacnb2		2.18			3.40
Cacnb3		2.87			
Cacul1	2.23				
Cald1		2.25	2.64		
Calhm5		2.09			
Calml3	2.25				
Caln1		10.47			
Camk2d	2.07				
Camk4	2.63	2.27			
Camp	14.02				
Camsap1	2.32				
Camsap2	2.26				
Capn5		3.03			
Car9	2.37				
Carmn	7.21	3.95			2.13
Casc3	2.01				
Cask				2.25	
Casr	6.28				
Castor2		2.10	2.28		
Cbfa2t2	3.02		2.05		
Cblb				3.18	

Ccdc102a	2.23				
Ccdc106	2.08				
Ccdc117	2.99		2.21		
Ccdc149		3.29			
Ccdc3		4.00	9.19		
Ccdc68		2.38			
Ccdc8		3.85			
Ccdc84			2.25		
Ccl3	3.23				
Ccn1		2.58			
Ccn3		20.28			
Ccn6	2.06				
Ccne1		2.67			
Ccnt1	2.42				
Ccser1		5.84			
Cd248		3.35			
Cd24a	2.29				
Cd53	3.45				
Cdc25a		2.10			
Cdc25b	2.81				
Cdc27	2.26				
Cdc42bpa				2.08	
Cdc42ep1		2.33			
Cdc42ep3	2.41				
Cdc42ep5			2.59		
Cdc45		2.33			
Cdh11		4.19	7.14		
Cdh2			4.25		4.69
Cdk17	2.23				
Cdk19	2.30				
Cdk5rap2		2.10			
Cdk7	2.26				
Cdkn1c	2.38	2.45			
Cdon				2.93	
Cds1	2.36				
Cdt1	2.40				
Cela1		7.64			
Celf1	2.37		2.01		2.17
Cemip2		3.72	2.39		
Cenpc1	2.05				

Cenpp	2.27				
Cercam		2.39			
Cers4		2.55			
Cers6		2.26			
Cfh		3.14	8.57		
Champ1	2.38				
Chd6	2.20				
Chn1		2.98			3.64
Chn2		9.21	3.44		
Chpf		2.26			
Chpf2		2.37			
Chrna7		7.01			
Chst12		2.41			
Chst8		3.64			
Cilp2		3.16			
Cish	8.55	2.63			
Cited1		9.70			
Clasp2	2.45				
Clcn6	2.19				
Clec11a		2.97			
Clec3a	2.11				
Clip2		2.41			
Clk4	2.29				
Clmp		2.45			
Clpx	2.27				
Clstn2	2.12				
Clstn3	2.33				
Cmbl		2.20			
Cmtm3			3.28		
Cmtm4		2.13	2.35		
Cnksr2	7.48				
Cnksr3		2.41			
Cnn2	2.77	5.00	4.78		
Cnn3		2.64			
Cnot2	2.07				
Cnot6	2.10				
Cnot6l	2.49				
Cnp		3.71			
Cntfr		2.83			
Cobll1	2.55				

Coil	2.01			
Col12a1		2.68		
Col13a1		4.40		
Col15a1	2.48			
Col16a1		2.28		
Col1a1		2.88	4.88	
Col1a2		2.66	2.61	
Col24a1	2.77			
Col3a1		3.15	3.99	
Col5a1		2.16		
Col5a2		2.31	2.27	
Col6a5		21.41		
Col6a6				7.20
Col8a1		5.97		
Col8a2		2.36		
Colec12				2.70
Commd9			2.33	
Copa				2.19
Copz2		2.35		
Coro2b		3.45	6.25	
Cpeb3	2.60	2.62		
Cped1		2.26		
Cpsf4	2.54			
Cpxm1		2.81	3.20	
Cpxm2		4.23		
Cpz		5.61		
Crabp1		3.73		
Crabp2		2.92	6.86	
Cramp1l	2.31		2.74	
Crebrf	3.58			
Crebzf	2.23		2.18	
Creld1	2.14			
Crispld2		2.02		
Crkl	2.21			
Crlf3	2.17			
Cryaa		15.65		
Csmd1		23.84		
Csnk1g1	2.72			2.21
Csrnp3	4.83	4.15	3.38	
Csrp1		2.03	2.11	

Ctdspl	2.14				
Cthrc1		2.62	2.11		
Ctnnal1	2.22				
Ctnnd1	2.27	2.28			
Ctsc		2.08			
Ctsk		2.42			
Ctu1	4.20				
Ctxn1		2.02			
Cxcl14		6.79			
Cxxc5	2.41				
Cybrd1		3.90			
Cygb		5.27			
Cyp26a1	5.73				
D030028A08Rik	3.64				
D130062J10Rik	4.14				
D17H6S53E	2.21				
D1Ertd622e	2.60				
D430019H16Rik		7.43			
Daam2		2.88		3.73	2.03
Dab2		2.84	2.04	3.77	
Dact1	4.23	2.04			
Dapk1				3.96	
Dapk2		2.85			
Dcaf12	2.21				
Dcaf12l1		3.07			
Dcaf5	2.23				
Dcbld1		2.19			
Dcbld2	2.19				
Dclk1		2.99			
Dclk2		2.59			
Dcn		4.44			
Dcp1a	2.47				
Dcun1d3	2.68				
Ddah1	2.18				
Ddah2		2.74	3.95		
Ddx3y	2.75				
Dedd2	3.45				
Dennd1a				2.88	
Dennd3	3.05				

Dennd4a	2.57		2.00		
Dennd4c	2.01				
Depdc5	2.28				
Des	3.65				
Dgka	2.65	2.05			
Dgke	2.67				
Dhx33	2.80				
Dhx57		2.16			
Dio3os	4.82				
Dkk2				2.58	
Dkk3		3.30	11.77		
Dlc1				2.13	2.27
Dleu2				2.43	
Dlg2	2.03	4.81	4.54		
Dlst	2.01				
Dlx5		2.77			
Dlx6		2.84			
Dlx6os1		2.62			
Dmac2	2.16				
Dmpk		2.25			
Dnm1				2.21	
Dnm3os	4.18	3.04	3.12		
Dnmbp	2.03				
Dock10	2.49	2.70			2.41
Dock11	2.44				
Dock4		2.16		3.92	
Dok1	2.86				
Dpagt1		2.33			
Dpep1		6.13			
Dpysl3	3.53	3.56	4.79		
Dsc3	6.73				
Dse	2.17		2.09		
Dtl		3.02			
Dtna	3.30				
Dtx2	3.12		2.00		
Duoxa1		3.26			
Dvl2	2.25				
Dyrk1a	2.42				
Dyrk1b		2.69			
Dzip1l		2.68			

E130102H24Rik	4.29				
E130114P18Rik	2.51				
E130308A19Rik		2.65			
E230001N04Rik	2.89				
E330013P04Rik	2.31				
Ebf1				3.01	2.34
Ebf2	6.11			2.48	3.21
Ebf3	3.18				
Ebf4		2.76			
Ece2		3.14			
Ecm2				2.25	
Eda		2.44			
Edc3	2.69				
Ednra		3.51			2.03
Efna1	2.05				
Efna4		2.69			
Efna5		5.20	4.51		
Egfl6		5.24			
Egfr		2.83	2.89	3.33	
Egr2	3.00				
Egr4	3.31				
Ehbp1				2.37	
Ehd3		2.76			
Eid2	2.53				
Eid3	2.54				
Eif2ak3	2.63				
Eif2b1			2.07		
Eif2s3y	2.10		2.19		
Elf2	2.01				
Ell	4.44				
Elmo1		2.91			
Eln		4.70			
Elovl5		2.22			
Emid1		3.84			
Emilin2	2.37				
Eml1			2.63		
Emx2	6.71				
Emx2os	10.95				
Enah	2.03	4.14			
Engase	2.14	3.77		10.06	

Eno2	2.83	2.10			
Eno3		2.66			
Enpp2		2.27			
Entpd2	3.08				
Entpd3		2.95			
Entpd5		2.07			
Epb41l3	2.67	3.31			
Epc1	2.18				
Epha3	6.36	3.49	8.27		
Epha4		4.51			
Ephb3		2.21			
Erbin	2.43				
Ercc8	3.21				
Esco1	2.15				
Esr1		2.34		5.17	
Etl4	2.42	3.18			
Etv6				2.10	2.14
Evc		2.18			
Evi2a		3.45			
Exo5	3.52				
Ext1		2.22		2.14	
Eya1	11.24	3.27	9.30		
Eya2	3.43	2.78	3.50		
Eya3	2.49				
Eya4		4.79			
Fads1		2.52			
Fads2		2.36			
Faim2		10.20			
Fam110b	2.01	2.06			
Fam135a				3.85	
Fam13a		3.91			
Fam13b	2.59				
Fam160b1	2.79	2.45			
Fam167a		2.42			
Fam171a1		2.70	3.71	3.15	
Fam171a2		3.44			
Fam180a	2.56				
Fam184a		2.57			
Fam185a	2.18				
Fam193a	2.41				

Fam193b	2.25				
Fam20a			4.56		
Fam222b	4.13				
Fam32a		2.04	2.72		
Fam3c		2.28			
Fam57a		2.89			
Fancf	3.08				
Fap		2.05			
Farp1	2.04			2.40	
Fastkd3	2.67				
Fat3		5.25			
Fat4		3.94			
Fbln1	2.57	6.80			
Fbln5		3.11			
Fbn2		2.09			
Fbxl19	2.06				
Fbxl20	2.27				
Fbxl8	2.15				
Fbxo42	2.76				
Fbxo46	2.50				
Fbxw11	2.08				
Fcer1g		8.29			
Fcgr3	3.29				
Fchsd2				2.25	
Fem1a		2.05			
Fen1	2.86				
Fes	2.58	3.05			
Fgd3		7.42	2.91		
Fgd4	2.64	2.30			
Fgfr2		2.41			
Fgl2		3.57			
Fhl2	3.88				
Fhl3	2.19				
Fhod1		2.24			
Fhod3		5.81			
Fignl2	2.59				
Filip1l					2.09
Fiz1	2.26				
Fjx1			2.86		
Fkbp11		2.15			

Fkbp14		2.27			
Fkbp7		3.30			
Fktn			2.03		
Flcn	2.26				
Fli1		2.27			
Flot2			2.33		
Flrt2	3.83	4.59	4.76		
Flrt3		2.06			
Fmnl2	2.91	3.28	3.05		
Fmnl3	2.11				
Fmo1		2.90			
Fnbp1l	2.08		2.33		
Fndc1	2.09	4.84	5.16	2.50	
Fndc4		3.78			
Fnip1	3.19				
Fntb	2.07				
Foxa3	2.44				
Foxd2	3.23				
Foxj3	2.93				
Foxk1	2.04				
Foxo1				2.13	
Foxp4	2.02				
Fpgs		2.13			
Fras1	2.79	3.66			
Frk		5.36			
Frmd4a					2.40
Frmd4b	2.84		2.21	3.54	
Frmd5		4.47			
Frmd7	3.06				
Frs2	2.59	2.31	2.33		
Fsd1l	2.02				
Fstl1		2.07			
Fto				2.26	2.05
Fut11		2.53			
Fzd1		2.17			
Fzd10	4.21				
Fzd2		2.19			
Gab1	2.82				
Gabra3		9.59			
Gabrb3		9.53			

Gadd45g	2.55				
Gal3st4		3.54			
Galns		2.21			
Galnt17		4.15			
Galnt5		3.86			
Galr2	4.66	3.07			
Gamt		2.85			
Gapvd1	2.16				
Garnl3		3.60			
Gask1a	3.85	10.52			
Gask1b	3.55	2.47			
Gatad2b	2.41				
Gbf1				2.46	
Gcnt2	2.59	2.41			
Gdf11		2.96			
Gdf5	2.44				
Gfra4		2.90			
Gga2		2.15			
Ghr				2.49	
Gigyf1	2.54				
Git1		2.32			
Gjc1	2.05	2.03	2.32		2.14
Gjc3	2.67				
Gli2			2.10		
Glis1	2.10				
Glt8d2		2.41			
Glul		2.63			
Gm10069	2.01				
Gm10130	2.23				
Gm10389	9.38				
Gm10550	3.49				
Gm10563	3.02				
Gm11714	3.07				
Gm11747	2.43				
Gm11837		2.37			
Gm12122		5.58			
Gm12905	2.01				
Gm13832		3.13			
Gm15551	10.26				
Gm15687	2.87				

Gm15708	4.36				
Gm15738	2.29				
Gm15787	2.40				
Gm16066	3.36				
Gm16068	4.50				
Gm16223	36.58				
Gm16552	2.61				
Gm16833		3.35			
Gm17435	2.60				
Gm17477	3.91				
Gm17529	2.47				
Gm19522	2.19				
Gm19583	3.19				
Gm19744	2.77				
Gm19967	5.80				
Gm20045	4.98				
Gm20594	10.51				
Gm20682	2.79				
Gm20712		2.30			
Gm20732	2.45				
Gm24362	4.35				
Gm266	2.02	11.97			
Gm26632					3.91
Gm26652	2.48				
Gm26724	3.17				
Gm26749	2.37	2.10			
Gm26827	3.09				
Gm27241	2.74				
Gm28054	2.17				
Gm28323		10.16			
Gm28501	3.09				
Gm28707	4.41				
Gm28791	3.19				
Gm29157		3.08			
Gm29417	3.67				
Gm29453		2.69			
Gm32219		3.31	2.94		
Gm34342	4.38				
Gm34728	3.32				
Gm35024		2.03			

Gm35082	8.61		
Gm36198	9.34		
Gm36827		6.92	
Gm37768	3.32		
Gm38115	4.79		
Gm38134	2.95		
Gm38190	4.69		
Gm38642	4.04		
Gm39090	3.04		
Gm39185	7.04		
Gm42517		4.59	
Gm42556	3.68		
Gm42659	2.88		
Gm42937	3.40		
Gm42941	3.86		
Gm43120	6.52		
Gm43138	4.66	2.92	
Gm43149	5.85	6.00	
Gm43328	3.28		
Gm43329	2.98		
Gm43330	4.59		
Gm43331	2.87		
Gm43387	2.39	2.72	
Gm43462	3.47	5.34	4.31
Gm43560	5.34		
Gm43623		4.75	
Gm43696	3.07	3.46	
Gm43707	5.24		
Gm43727	2.12	2.62	
Gm43774	3.95		
Gm43813	4.68	2.42	
Gm43920	4.16		
Gm44190	4.25		
Gm44686	2.88		
Gm44699	2.96		
Gm45025	2.97		
Gm47101		4.61	
Gm47167	2.85		
Gm47664		2.89	
Gm47689	8.26		

Gm48025		6.54			
Gm48678	3.48				
Gm48742		2.61			
Gm49189	3.72				
Gm49490		5.15			
Gm49692	3.03	3.85			
Gm525	5.27				
Gm527	3.35				
Gm6133	2.84				
Gm7292	56.20		7.60		
Gm7324	25.91	18.21	39.88		
Gm8797			2.66		
Gm9774	3.64				
Gmeb1	2.06				
Gmeb2	2.87				
Gmppb	2.02	3.61			
Gnai1		2.71			
Gnao1	3.19	4.50			
Gnasas1			4.06		
Gng12	2.04				
Gng8		3.70			
Gnptab		2.10			
Golph3l	2.05				
Gpank1	2.28				
Gpatch8	2.30			3.46	2.41
Gpc2		2.84			
Gpc3		2.07			
Gpc6					2.11
Gpha2		26.72			
Gpm6b	2.04	3.06	2.04		
Gpr135		2.26			
Gpr146	3.06				
Gpr153		2.40			
Gpr173		2.47			
Gpr180		2.40			
Gpsm2	2.00				
Gpsm3	2.05				
Gpx3		6.76	2.74		
Gpx7		2.39			
Grb10	2.24	2.29		2.15	

Grb14		2.88			
Gria1	4.85	4.04			
Grin2a		22.86			
Grk3	2.09				
Grm7		16.39			
Gsc		2.05			
Gsdmd		2.96			
Gsn				2.48	
Gstt1		2.88			
Gtf2f2	2.00				
Gtf2ird2	2.20				
Gtpbp3	2.27		4.07		
Gtpbp6	2.51				
Gucy1a1					2.23
Gucy1a2					2.22
Gulp1	2.61	3.00	3.19	2.90	
Gxylt2		2.97			
H2-DMb1		2.01			
Hacd4		2.25			
Hace1	2.06				
Haus6			2.97		
Hba-a2	4.98				
Hbp1	2.35				
Hcar2	2.59				
Hdhd3		3.15			
Heatr5a	2.00				
Heca	2.24				
Hectd2	3.65				
Hecw2		2.98			
Heg1				2.65	
Hic1		3.05			
Hic2	3.37				
Hip1	2.36				
Hipk2				3.25	
Hira	2.25				
Hivep1	2.38				
Hivep2	2.02				
Hjurp	2.77	2.13			
Hlx	4.09	2.26			
Hmcn1		3.85	6.28		

Hmgcl	2.12			
Hmgcll1	2.82			
Hnrnpll	2.49			
Hopx	2.33			
Hoxa10	2.11	2.49		
Hoxa11os				8.82
Hoxa5	2.92			
Hoxa9	2.25		2.02	
Hoxc4	2.14	2.84		
Hoxc5	2.79			
Hpcal1		2.21		
Hpgd		3.88		
Hpse2		39.67		
Hs2st1	2.96			
Hs3st3a1		4.91		
Hs3st3b1		2.17		
Hs6st2	2.92			
Htra3		2.34		
Htt	2.19			
Hyal2	2.54			
Hykk		2.04		
Icam1	2.14		7.00	
Id1	2.10			
Id3	2.48			
Ifitm10	2.05			
Ift81		2.21		
Igdcc4		2.23		
Igf1		4.48	15.59	2.56
Igf2bp2	2.40			
Igfals	3.21			
Igfbp3		6.42		
Igfbp6			5.59	
Ighm	9.44			
Igsf3		2.33		
Ikzf2		2.92		
Il13ra1		2.74		
Il16	2.25			
Il17rd		3.20		
Il1b	2.54			
Il1rn	5.41			

Il34					3.90
Inka1	3.29				
Insc		2.39			
Ints5	2.35				
Ints7	2.35				
Intu		4.04			
Invs	2.24	2.09			
Ip6k1	2.09				
Ip6k2	2.39				
Iqcb1	3.22				
Iqce		2.07			
Iqgap2	2.22				
Iqsec2	2.21	2.39	2.14	2.74	
Irf2	2.21				
Irf2bp1	2.57				
Irf2bpl	2.27				
Irs1		2.57			
Ism1				3.06	
Itch	2.02				
Itga11		2.67			
Itga8	3.39	3.10			
Itgbl1	3.08	4.51	7.99		
Itpkb	2.87	2.48	2.71		
Itsn1				2.12	
Itsn2	2.81				
Izumo4		2.54			
Jag1		2.03			
Jmjd1c	2.16				
Jph4		4.65			
Jrk	5.52				
Junb	2.09				
K230015D01Rik		2.28			
Kalrn	3.20		3.28		
Kansl1	3.54				2.17
Kansl3	2.05				
Kat14		2.08			
Kat2b	2.12	2.34		2.10	
Kat7	2.66				
Katnbl1	2.48				
Kcnb2		5.65			

Kcnj15		2.41			
Kcnj2		3.61			
Kcnj8					2.58
Kcnk1		2.60			
Kcnk6		3.87			
Kcnn1		5.37			
Kcnq3	4.30				
Kcnt2					5.22
Kctd1		2.57			
Kctd11		2.74			
Kctd12		2.68			
Kctd12b		2.31			
Kctd14		2.15			
Kctd4	2.45				
Kctd9	2.25		2.14		
Kdm2a	2.30				
Kdm4c	2.44				
Kdm5a	2.02				
Kdm5c	2.09				
Kdm5d	2.85				
Kdm6a	2.44				
Kera		11.58			
Khnyn	2.92				
Kif13a	2.01				
Kif13b				2.43	
Kif1a	5.11	8.80			
Kif21a	2.14				
Kif7	2.19	2.49			
Kirrel	2.03		2.06		
Kl	3.09				
Klf11	2.24				
Klf12				2.02	
Klf16	2.30				
Klf6	2.07				
Klf8		3.32			
Klhdc10	2.34				
Klhl12	2.21				
Klhl13				2.84	
Klhl18	2.09	2.01			
Klhl24	2.41				

Klhl26	2.22				
Klhl7		2.02			
Kmt2e	2.19				
Kremen1		2.20			
Krt75		20.08			
Krtcap3	2.66				
Ksr1		2.44			
Kti12	2.30				
Lama3		4.82			
Lama4				2.13	
Lamb1		2.04			
Lamc1			2.08		
Laptm4b	2.41				
Larp6	2.58	4.04			
Lbp		5.31		3.26	
Lca5l		2.96			
Lcmt2	2.93				
Lcn2		9.04			
Lcor	2.23				2.64
Lcp2		5.78			
Ldb2		2.45		6.62	2.03
Lef1	3.26				
Lemd3	2.88				
Lgals1			3.16		
Lhfp		3.04	2.65		
Lhfpl2			2.71		
Lifr		2.11			
Lilr4b	2.92				
Lima1		2.80	2.44		2.26
Limd1					2.27
Lims1					2.11
Lin52	2.09				
Lin54	3.08				
Lin7a					4.15
Lmbr1l	2.11				
Lmo7		2.71			
Lmx1a		5.10			
Lmx1b	3.52				
Lncpint	3.30			2.48	
Lncppara	4.38	2.60	2.75	6.46	

Lnx2		2.98	2.39		
Lonrf1	2.70				
Lonrf2		3.68			
Lox		3.73	2.81		
Loxl1		2.06	2.21		
Lpar1				2.23	
Lpcat3	2.35	2.10			
Lpl		2.03	2.77	3.76	
Lrch1	2.84				
Lrig3	2.31	2.47	2.19		
Lrp3		2.49			
Lrp4		2.41			
Lrrc1	2.82				
Lrrc15		7.16			
Lrrc17		3.75			
Lrrc4b		5.59			
Lrrc73	2.40				
Lrrn1		6.63			
Lsamp				8.16	
Ltbp1		2.29			
Ltbp4		2.29			
Ltf	16.55				
Lum		3.68	2.41		
Ly6c2	3.22				
Lyst		2.02			
Lyz2	2.13	6.16			
Mab21l2	2.35				
Maco1	2.39	2.12			
Macrod2		2.02			
Maea	2.13				
Magi2				2.36	
Majin	3.06				
Mamdc2	5.34				
Maml2				2.77	
Maml3	2.25				2.75
Man1a				2.02	
Man1c1		2.29			
Map1b		2.59			
Map2k6		4.45			

Map3k1	2.83				
Map3k2	2.11				
Map3k21	3.34				
Map3k5				6.34	2.33
Map4k3	2.16				
Map4k4	2.12				
Map4k5	2.58				
Mapk12	2.07	3.73			
Mapkapk3	2.53				
Marcks	2.77		2.78	2.43	
Mark1					2.11
Mark4			2.07		
Masp1		7.88			
Mast4		2.18			2.03
Matn1			3.33		
Mau2	2.32				
Max	2.02				
Mb21d2	3.43	2.16			
Mbd4		2.69			
Mboat1	2.04	4.74	3.23		
Mbtd1	3.45				
Mdk	2.32	2.66	2.24		
Mecp2	2.40				
Med4			2.14		
Mef2d	2.09				
Megf6	3.94	3.76	4.31		
Megf9		2.87			
Meis3		2.32			
Memo1	2.01				
Men1		2.03			
Meox2					2.04
Metap1	2.12				
Mettl24		2.80	5.66		
Mettl25	2.12				
Mex3a	4.40	2.31	3.48		
Mex3d	2.31				
Mfap2		3.56	2.33		
Mfap4	2.13	11.01	10.14		
Mfap5		3.03			
Mgat4c		7.31			

Mgst1	2.42				
Mgst3			4.41		
Miat	6.25	5.22	5.57		
Micall2	2.32				
Mid1		2.26			
Mid1ip1	2.03				
Mid2	2.18				
Mief2		2.31			
Mif4gd		2.26			
Mir100hg		2.06		2.10	
Mir99ahg				3.97	
Mitf		2.13		4.16	
Mki67		4.04			
Mkln1	2.34			2.70	
Mkx		3.47			
Mlxip	2.67	2.71			
Mmp11		3.24			
Mmp13	3.45	2.10	5.70		
Mmp14			2.17		
Mmp2			2.30		
Mmp23		5.75	4.28		
Mms19	2.70		2.16		
Mnt	2.68				
Mob2	2.09				
Mob3b		3.63			
Mob3c	4.05				
Mocs3	2.66				
Mon2	2.44		2.04		
Mpp2		2.78			
Mpp5	2.10				
Mpped2	2.56	2.75			
Mrgbp	2.06				
Mrgprf		3.30			
Mrpl50	2.28				
Mrtfa		3.04		3.05	3.34
Mrtfb	2.03				
Mrvi1					2.43
Msl2	2.03				
Msmp	19.07		544.88		
Mtmr10	2.21				

Mtmr11	2.83				
Mtmr12	2.26				
Mtmr14	2.50				
Mtmr3	3.35			2.58	
Mtss1	2.40				
Mtus1					3.21
Mxd4		2.14			
Myl9		5.66	4.48		
Mylk		5.49			
Myo16		11.87			
Myo19		2.45			
Myo1b		3.18	5.33		2.73
Myo7a		2.21			
Myo9a				2.06	
Myoc				4.89	
Myorg		5.23			
Myrip	2.35				
N4bp2		2.23			
N4bp3	3.88				
Naa15	2.31				
Naa16	2.17				
Naa25	2.43				
Naa40	2.46				
Naaladl2				3.05	
Nalcn	2.22	2.05			
Nanp		2.44			
Nav3		4.12			
Nbl1	3.94	2.91	3.14		
Ncald		2.96	4.42		
Ncam1		2.72			
Nck2	2.22		2.07		
Ncoa1	3.06	2.38		2.70	
Ncoa2				2.28	
Ncoa5	2.39				
Ncoa6	2.05				
Ncs1			2.06		
Ndnf		3.18			
Ndp	3.09				
Ndrg4		2.01			
Ndst2	2.65				

Ndufaf7			2.19		
Negr1				8.03	
Nelfa	2.17				
Neo1		2.38			
Neurl4	2.63				
Nfatc4	2.57	2.78	3.94		
Nfia				2.09	
Nfil3	2.05				
Nfkb1	2.01				
Nfrkb	2.14				
Nfxl1	2.29				
Nfyc	2.36				
Ngp	45.06				
Nhs					3.94
Nhsl1		2.00			
Nhsl2		3.35	2.33		
Nid1		2.08			
Nin	2.59	2.33			
Nipa1		2.66			
Nkapd1	2.01				
Nkd2		2.20			
Nlgn1	2.53	4.25			
Nlgn2			3.22		
Nlrp3	3.27				
Nmt2	2.05				
Nob1	2.08				
Nog		2.10			
Nol3		2.65			
Nop9	2.48				
Nova1				2.26	
Nox4		13.95			
Npas2	2.21	2.54			
Npat	2.00				
Npdc1			2.11		
Npepps	2.43				
Npnt	2.94	3.39	4.64		
Nppc		22.40			
Nptxr		4.32			
Npy1r					2.73
Nr2c2	2.29				

Nr2f2					2.42
Nr4a3	2.21				
Nr6a1	3.05				
Nr6a1os		7.30			
Nrbp2		2.03			
Nrep					2.13
Nrg1		5.56			
Nrgn		8.13			
Nrm		2.26			
Nrp2	2.33				
Nsd2	2.79		2.21		
Nsg1		4.17			
Nt5c2		3.03			
Nt5dc2		2.64			
Ntn3		2.09			
Ntng2	4.19				
Nuak1		2.12			
Nudt18		2.30			
Numb				2.33	
Nup153	2.86				
Nup98	2.22				
Nutf2	2.02				
Nxn		2.89	2.03		
Nynrin		2.12			
Ocel1		2.48			
Olfml1		2.87		3.77	
Olfml2b		3.07	4.24		
Olfml3	2.13	3.22		2.07	
Olfr558					2.06
Omd		2.17			
Ooep		3.09			
Ophn1	2.47	2.01		2.99	
Optn	2.31				
Orai3	2.49				
Osbp2		2.52			
Osbpl11	2.27	2.01	2.15		
Osbpl9				2.18	
Osr1		5.30			
Ostn		3.37			
Otor	2.95				

Otud1		2.02			
Otud7b	2.11				
Oxct1		2.46			
Oxld1	2.26				
Oxr1	2.08				
P2rx2		13.96			
P2ry1		3.05			
P2ry10b		2.36			
P3h2					2.55
P4ha3	4.04	5.94	5.90		
Pacs1	2.07		2.07		
Pak1		2.88			
Pak4	2.13				
Palld		3.60			
Palmd		3.12			
Pamr1		3.81	10.62		
Pan3	3.20			2.54	2.30
Papolg	2.12				
Pappa2	2.29				
Papss2	2.26				
Paqr4		3.90			
Pard6g		2.10			
Parm1	3.57	8.15			
Parp8	3.29	2.81	3.28		
Pawr		2.47			2.99
Pbx1		2.74	2.10	2.22	2.53
Pccb		2.35			
Pcdh18		2.73			
Pcdhb14		2.48			
Pcdhb17		2.09			
Pcgf5	2.16				
Pcnx	2.50				
Pcolce		2.03	2.07		
Pcp4l1	3.34				
Pcsk7			2.33		
Pde10a	2.00				
Pde1a					2.75
Pde3a	2.42				2.27
Pde3b			2.34		
Pde5a		2.41			2.20

Pde7a	2.02		2.05		
Pde8a	2.05				
Pdgfd		4.93		5.56	
Pdgfra	3.01		3.40	2.24	
Pdgfrb			2.39		
Pdgfrl		3.53		2.60	
Pdlim2		2.48			3.15
Pdlim5		2.12			2.27
Pds5b				2.54	
Pdzrn3			2.41		
Pdzrn4		5.50	5.33		
Peg12		2.15			
Peli2					2.40
Pelo	2.52				
Pgap1		3.07			
Pgm5		2.27	6.64		
Pgpep1			2.70		
Phc2	2.20				
Phc3	2.21				
Pheta2	2.98	3.07			
Phf12	2.11				
Phf13	4.14		2.34		
Phf21a	2.32			2.33	
Phf23	2.82				
Phf3	2.16				
Phf6	2.15				
Phip	2.22			3.08	
Phldb2		5.42			
Phlpp1		2.83			
Pi15		2.10			
Pi4k2b	2.22				
Pi4ka	2.07				
Pianp	3.87				
Pias1	2.47			2.46	
Pias2	2.29				
Pias4	2.30				
Pid1		2.30		2.34	
Piezo2		2.92			
Pigt		2.13			
Pik3ap1	3.55				

Pik3c2b		4.09			
Pik3r1		2.62		3.39	
Pik3r2			2.68		
Pik3r4		2.45			
Pip4k2a		3.22			
Pip5k1a	2.96				
Pitpnm3	2.89				
Pitx1	2.69		2.05		
Pkia		5.64			
Pknox2	2.43				
Platr25		2.16			
Plb1		2.68			
Plcb4		3.93			
Plcd3	2.53				
Plce1				2.05	3.10
Plcg2	2.23				
Plcl1	3.30	4.09			3.64
Plcl2	2.39				
Pld2		2.24			
Plekha1	2.11				
Plekha5	2.26			3.02	
Plekhf1	2.48	3.33			
Plekhg1		2.66		3.76	
Plekhg3	2.28		2.21		
Plekhg4		4.65			
Plpp1		2.28		2.17	
Plpp3				2.53	
Plpp7	3.36				
Plppr4	2.96	6.39	4.03		
Plppr5		12.66			
Plxdc2				3.44	
Plxna2		2.68			
Plxna4		5.12			
Pms2	2.30	2.48			
Podnl1		2.77	2.80		
Podxl2	3.22	5.28	6.77		
Polq			2.28		
Polr3e	2.03				
Pomgnt2	2.50				
Pop4			2.12		

Postn	5.64	7.18	17.23		3.87
Pou2f1	2.74			2.66	
Ppan	2.50				
Ppfia2		3.44			3.30
Ppfibp2		2.98			
Ppl				7.32	
Ppm1e					4.21
Ppnr	2.75				
Ppp1r12a					2.65
Ppp1r12b					2.68
Ppp2r2a	2.12				
Ppp2r5b	2.21				
Ppp4r1	2.45				
Ppp6r1	2.05				
Pprc1	2.55				
Pptc7	2.10				
Pradc1		2.02			
Prdm11	2.42		2.76		
Prdm5	2.00	2.09		4.04	
Prickle1		2.38		3.07	
Prickle2		3.20			
Prickle3	2.64				
Prkaa1	2.22				
Prkag2		2.32			
Prkd1				2.15	
Prkd2	2.29				
Prmt6	3.58				
Prorp	2.38				
Proser2	2.02				
Prpf3	2.36				
Prpf39	2.19				
Prr16		2.73			
Prr5		2.45			
Prrx1	2.08	2.68	3.17	2.11	2.47
Prrx2		2.56			
Prss23		3.01	2.96		
Prss35		3.89			
Prtg		2.84			
Prx		2.58			
Pstpip2	2.29				

Ptbp2	3.14		2.21		
Ptcd1	2.09				
Ptcd3	2.12				
Ptchd4	2.62				
Ptges3l		2.25			
Ptgfr		3.38			
Ptgfrn		3.13	2.32		
Pthlh		4.05			
Ptn		4.97	8.70		
Ptpn13		2.86	3.02		
Ptpn3	2.03				
Ptprd	2.09	2.98		3.16	
Ptprk					2.03
Ptprn2	3.48				
Ptpru		2.02			
Ptx3	2.03				
Pum2	2.04				
Pxk	2.05	2.94	2.38		
Pygo1		2.35			
Qrich1	2.22		2.05		
Qtrt1	2.33				
R3hcc1l	2.32				
Rab17	2.70				
Rab20	2.46				
Rab27b		4.24			
Rab29		3.74			
Rab30	2.48	3.98		4.81	
Rab31	2.05				
Rab3a	2.78	2.31			
Rab3gap2	2.45				
Rab3il1		2.66	4.55		
Rab40c	3.76				
Rab4a		2.00			
Rab6b	8.54	5.38			
Rab7b		2.27			
Rab8b	2.91				2.54
Rac2	3.40				
Radil			3.29		
Rai14	2.31	2.08	2.34		
Rai2		3.13			

Ranbp17	2.33	2.20			
Ranbp9	2.56		2.23		
Rap1gap2	2.61		2.83		
Rapgef2				2.43	
Rapgefl1	2.44				
Rarres2		3.95			2.20
Rasa2	2.05				
Rasal2		3.93	2.52		2.32
Rasgrf2		5.16			
Rasl11b		2.36			
Rassf2		3.01			
Rassf8		2.18			
Rbm6				2.32	
Rbms3					2.38
Rbp1		2.50			
Rbpms					2.67
Rcl1	2.03				
Rcn1		2.27			
Rcn3		2.28			
Rcor1	2.03				
Reck				2.34	
Reep1		4.18	2.96		
Rem1					3.88
Reps2	3.15	6.18			
Rere				2.02	2.16
Rerg	2.08				
Retnlg	3.34	10.09			
Retreg1		2.78	2.70		
Retreg3	2.26				
Rffl	3.04				
Rflnb		2.05			
Rftn1		3.00			
Rfx2			2.77		
Rfx5		2.63			
Rfx7	2.13				
Rgl1	2.54	3.70		3.63	
Rgs3		4.54			
Rgs6				5.65	5.55
Rhbdf2	3.08				
Rhno1	2.28				

Ric3		3.11			
Rictor	2.90				
Rilp	2.84				
Rims1		3.47			
Rin2	2.21			2.40	
Rin3	3.36	2.96	2.63		
Rinl	4.24				
Riok1	2.09				
Riox1	3.05				
Rit1	2.76				
Rnase4	2.15	3.14	2.93		
Rnf111	2.40				
Rnf113a2		2.04			
Rnf115	2.16				
Rnf122	2.38	4.57			
Rnf128	2.27				
Rnf144a	2.28	5.42			
Rnf144b	2.49				
Rnf157	2.32	2.64			
Rnf180	3.80				
Rnf182		6.82			
Rnf185	2.82				
Rnf2	2.03				
Rnf217			2.26		
Rnf38	3.82				
Rnf41	2.13				
Rngtt	2.14				
Robo2		2.37			
Ror1					3.66
Ror2		3.53	2.62		
Rpl39l	2.24				
Rprd2	2.46				
Rprml	2.97				
Rps6ka1	2.23				
Rpusd4	2.23				
Rreb1	2.27	2.09			
Rrnad1	2.55				
Rsbn1	2.29				
Rsbn1l	2.04				
Rsf1os1	2.56				

Rspo2		7.60		10.77	
Rspry1	2.45				
Rtn4r		4.22			
Rtn4rl1	2.13	3.62			
Rubcn	2.04				
Rufy3	2.40				
Runx1t1	2.64			2.55	2.92
Runx2	4.15	2.21			
Rusc2			2.63		
S100a16		2.60			
S100a4		2.05			
S100a9	3.30				
S1pr1		4.84	6.14		
Samd5	2.39	5.17			
Samhd1		2.55			
Sash1				2.36	
Sat2		3.09			
Scaf8	2.55			2.06	
Scara3				3.46	
Scmh1	3.28			2.77	
Scn1a	2.39				
Scn9a	8.34				
Scrn1		2.29			
Scube1	3.09				
Scube2	3.92	4.75	4.96		
Sdk1		3.25			
Sec16a	2.02				
Sec16b		3.09			
Sec22a		2.40			
Sec23a		2.03			
Sec24a	2.89				
Sec24b	2.01		2.02		
Sec24d	2.43	2.23		2.17	
Selenom		2.07			
Selenoo	2.36				
Selenop		2.99	2.96		
Sema3a		2.38			
Sema3b		2.43			
Sema3f		2.46			
Sema4c	2.02				

Sema6c		3.74			
Sema6d		2.56			
Serinc5		2.47			
Serpina3n	2.67				
Serpinb6b		4.83			
Serpinf1	2.05	3.79	3.90		
Serping1		6.26		2.05	
Sertad3	3.91	2.09			
Sesn1		2.19			
Sestd1	2.25		2.17		
Setbp1				2.33	3.13
Setd5	2.29				
Sez6l	3.74				
Sfmbt1	3.05				
Sfrp1	3.34	2.10	2.24		
Sfrp2		15.81			
Sfrp4		4.96			
Sgsh		2.58			
Sh3bgr	6.43				
Sh3bp2	2.96				
Sh3bp4	3.68		3.36		
Sh3bp5		2.29			
Sh3bp5l	2.17				
Sh3pxd2b		2.25			
Sh3rf1	2.29	2.11		5.53	
Sh3rf3	2.49	3.48			
Shank1		3.57			
Shc3	4.69				
Shisa4	2.20				
Shisal1	8.97	2.84			
Shld3	2.61				
Shox2	2.35	2.10			
Shprh			2.02		
Shq1	2.01				
Siah1a	2.46		2.02		
Sik1	2.06				
Sik3	2.17				
Sim2	2.56				
Six1		3.04			
Six2		10.97			

Six5		2.86		
Skp2	2.22			
Slc14a1	4.04			
Slc15a3	3.62			
Slc16a7		2.70		
Slc1a3		5.00		
Slc20a2		2.14		
Slc22a5	4.47	2.31		
Slc24a3				2.36
Slc24a4	4.82	8.31		
Slc25a27	2.06			
Slc25a29		7.11		
Slc25a35	4.34			
Slc25a47	2.04			
Slc25a53	2.05			
Slc2a4	3.97			
Slc35d1	2.72			
Slc39a13		2.32		
Slc40a1	4.18			
Slc41a3		2.39		
Slc43a1		2.39		
Slc43a2	3.26			
Slc4a3		2.80		
Slc6a17	3.27			
Slc8a1	3.37	5.90	5.99	
Slc8a3	2.40			
Slc9a5		3.58		
Slco3a1		2.39		
Slco5a1		2.03		
Slit2		3.71		
Slit3	2.90	2.72	5.61	
Slmap	2.86		2.15	2.08
Smad3	3.13			
Smad6	3.06			
Smarca1		2.11		
Smcr8	2.04			
Smg6				2.17
Smoc2		3.35		
Smurf2	2.02			
Snai1	2.93			

Snap23		2.21			
Snapc2	2.54				
Sncaip		6.80	9.82		
Sned1				2.67	
Snhg16	3.80				
Snhg17	2.99				
Snph	2.89				
Sntb2	2.11	2.11			
Snx18					2.37
Snx24				3.80	
Snx25	2.26				
Snx29				3.72	
Snx7		2.37			
Snx9	2.35				
Sobp	2.07				
Socs3	2.32				
Socs7	2.13				
Soga1	3.47	3.32	2.34		
Sorbs1					2.40
Sorbs2	3.08				
Sorbs2os	2.51				
Sorcs2		2.66			
Sorcs3	5.81				
Sorl1		3.52			
Sos2	3.19		2.73		
Sox4	2.33				
Sox5				3.24	2.31
Sox5os4					9.08
Sox7	6.07				
Sox8	2.04				
Sp7		2.33			
Spata7	2.58				
Spire1	2.18				
Spock2		3.49			
Spon1		2.98			
Spon2	3.09	5.61			
Sppl3	2.46				
Spred2	2.82	2.10			
Spry1	5.25				
Spsb2	3.09	2.15			

Srfbp1	2.06				
Srgap3		2.43		3.29	
Srp54c	2.15				
Srpx		2.04			
Srsf4	2.59				
Ssbp2	2.25				
Ssc5d		2.11			
Ssh2	2.07	2.50	3.10	3.09	
St3gal1				2.94	
St3gal4		2.55	2.52		
St3gal5		2.49			
St5	2.09	2.89	2.61		2.30
St6gal1		2.48			3.22
St6galnac2	2.46				
St6galnac3		2.31			
Stac					5.19
Stambpl1		2.50			
Stat3	2.47				
Stat5b	2.32	2.32		2.82	
Steap3				2.08	
Stil	2.40				
Stk17b		2.46			
Stk32c	2.01				
Stmn4		2.87			
Stox2			2.06		
Stradb		2.02			
Strip1	2.05				
Strn3	2.01				
Stx11	2.79		2.71		
Stx1a	2.78	2.60			
Stx3	3.73				
Stx5a	2.44				
Stxbp5	2.18				
Stxbp6				2.25	
Sufu	2.00	2.07			
Sugp1	2.07				
Sulf1		2.41			
Svep1	2.42			3.72	
Svil	2.55	3.72	2.72	2.39	
Sybu	3.48	4.85	3.58		

Syngr2			2.17		
Synj2	2.07	2.07	3.85		
Synm	2.51				
Synpo2					3.24
Syt9	6.58	6.14	3.77		
Sytl2		4.70			
Sytl5		6.11			
Tab3	2.08				
Tac1		9.63			
Tada1	2.20				
Taf1a	4.10				
Taf8	2.52				
Tagln		6.77			
Tanc1		2.44			
Tango2		2.18			
Tax1bp3			2.19		
Tbc1d10a	2.24				
Tbc1d23	2.66				
Tbc1d25	2.36	2.44			
Tbc1d5				2.11	
Tbc1d8b		2.63			
Tbcc	3.03	2.07			
Tbcd		2.11	2.23		
Tbkbp1	2.36				
Tbl1xr1			2.26		
Tbrg4			2.01		
Tbx15	2.34			2.70	
Tbx18	4.28	2.18	2.92		
Tbx2			2.65		
Tbx3	2.10				
Tbx3os1	2.95	5.12			
Tcf12	2.19		2.09	2.27	
Tcf7l1				2.94	3.35
Tec		3.09			
Tenm3	3.46	2.34		2.47	
Tent4a	2.58				
Tent5a		2.56			
Tet2	2.77				
Tfeb	2.07	2.18			
Tfip11	2.11				

Tgfb1i1		2.77			
Tgfb3		2.27			
Tgfbr3	2.57	2.09	2.05	2.43	
Tgif1	3.32				
Tgif2	2.87				
Thap11	2.39	2.09			
Thap4	2.28				
Thbd		2.04			
Thbs2	2.30	5.34	7.51		
Thbs3		2.52			
Thrb				4.34	
Thsd7a		2.92			
Thsd7b		12.16			
Thy1	7.20	2.46			
Ticam1	2.29	3.84			
Tifa	2.83				
Tigd5	8.27		6.89		
Timm29			2.07		
Timp2			2.03		
Tjap1	2.72				
Tlk1		2.13			
Tlk2	2.17				
Tll1	2.17				
Tln2		2.06			
Tlnrd1	2.24				
Tm4sf1		2.12			
Tmcc1				2.05	
Tmcc2		2.06			
Tmed3		2.44			
Tmeff1			2.34		
Tmem100	2.42	3.58			
Tmem11	2.80				
Tmem115	2.49		2.08		
Tmem119		2.76	3.60		
Tmem121	3.90	3.39			
Tmem131					2.09
Tmem132a		4.61			
Tmem132c		6.32			
Tmem135		2.08		2.97	
Tmem164	2.27			2.90	

Tmem176a					2.08
Tmem184b			2.10		
Tmem198b		2.13			
Tmem246	2.23	2.28			
Tmem26		5.26			
Tmem41b	2.23				
Tmem44		3.31			
Tmem47		2.15			
Tmem53		2.81			
Tmem87a				2.86	
Tmsb4x			2.59		
Tmtc2		3.45			
Tnc		2.36			
Tnfaip8l3		2.69			
Tnfrsf19		6.23			
Tnfsf12		2.09			
Tnmd	7.82	4.15	13.83		
Tnn		6.64	6.97		
Tnni2	2.80				
Tnxb	2.15				
Tom1	2.57				
Tom1l1		4.47			
Tom1l2				2.56	
Topors	2.10				
Tpm2		2.47	3.67		
Tpp2	2.03				
Tppp3		2.50	5.18		
Traf2	2.30				
Traf4	2.99				
Tram2	2.03				
Trappc12			2.13		
Triap1	2.10				
Tril		2.27			
Trim16		2.74			
Trim2		2.57	3.86		
Trim32	2.38		2.42		
Trim33	2.29				
Trim9	3.53				
Trit1	2.46				
Trmt10c	2.33				

Trmt12	3.06				
Trmt13	2.73				
Trp53rka	2.49				
Trpc3					4.02
Trpm3	2.34				
Trps1				2.19	
Tsc1	2.86				
Tsc22d2	2.40		2.04		
Tshz2				2.68	2.53
Tshz3			2.21		
Tspan13	2.83				
Tspan14		2.14			
Tspan18		4.59			
Tspan2		2.44			
Tspan7		3.28			
Tspan8	3.49				
Tspan9		2.12			
Tspyl1	2.49				
Ttc7b				4.41	
Ttll4	2.15				
Ttyh3		3.70			
Tub		9.57	5.46		
Tuba1a		2.82			
Tubgcp6	2.52				
Tunar	4.17				
Tut1	3.22				
Twist1			2.16		
Txnip	2.12			3.42	2.92
Txnrd3	3.05				
Tyrobp	2.01	5.79			
Uaca	2.85	2.34			
Ubald1	2.46				
Ubap1	2.19				
Ubash3b	2.09				
Ube2g1	2.08				
Ube4b	2.10				
Ube4bos1	4.10				
Ubn1	2.30				
Ubr1					2.38
Ubr5	2.23				

Ubtd1	3.27				
Ubxn7	2.95				
Uhrf1bp1l	2.12				
Uhrf2	2.48				
Ung	2.21	2.30			
Unk	2.16				
Unkl	3.45				
Usp13	3.49				
Usp24	2.33				
Usp28	2.98				
Usp29	3.14				
Usp32	2.30				
Usp33			2.20		
Usp36	2.08		2.03		
Usp47	2.08				
Usp53	2.32				
Usp54	2.17				
Usp6nl	3.54		2.27		
Uspl1	2.72				
Ust		2.32		2.15	
Uvrag				2.36	
Vash2		2.70			
Vcam1		3.17			
Vdr		2.03			
Vegfc		2.63			
Vgll4	2.25				
Vipr2		5.90			
Vkorc1l1	2.28				
Vps18	3.41				
Vps33b		2.02			
Vps37b	2.08				
Vps37c	2.41				
Vps37d	2.63				
Vsig10	2.41				
Vstm2b		4.93			
Vstm4		2.26			3.64
Vwce	2.34				
Wac	2.15				
Wdfy2	2.21	2.54	2.09		
Wdr37	2.47				

Wdr44	2.22				
Wdr53	2.60				
Wdr59	3.20				
Wfdc1		4.98	5.01		
Wfdc21	5.60				
Wipf1	3.23	3.47	4.23		
Wnt16		5.62	13.42		
Wnt5a		2.63			
Wnt5b		4.68			
Wwc2	2.23			2.32	
Wwp2	2.66				
Xbp1		2.09			
Xlr3c	6.91				
Xpo4	2.28				
Xpo6	2.48				
Xpo7	2.49				
Xylt2	2.43				
Yap1	2.14				
Ydjc		2.64	3.41		
Yif1a		2.05			
Ypel2	2.35				
Zbed4	2.18				
Zbed5	3.40				
Zbtb10	2.49				
Zbtb16	2.77	3.30		3.33	
Zbtb2	5.56		2.85		
Zbtb20				2.36	2.01
Zbtb34	2.03				
Zbtb39	2.74				
Zbtb43	2.44				
Zbtb45	3.80	3.45			
Zbtb7b	2.03				
Zbtb8a	2.89	4.33			
Zc3h3	2.28				
Zc3h7a	2.69	2.19			
Zcchc14	2.03		2.06		
Zcchc2	2.11				
Zcchc3	3.95				
Zcchc7				2.23	
Zdhhc14					4.70

Zdhhc7	2.72				
Zdhhc9		2.13			
Zeb1		2.19			
Zeb2	2.76	2.84	3.80	2.40	3.08
Zfand3	2.77				
Zfc3h1	2.14				
Zfhx3		2.06			
Zfhx4	2.09	2.37		2.92	
Zfp1	3.54				
Zfp110	3.39	2.09			
Zfp111	3.31	3.34			
Zfp120	2.13				
Zfp142	3.47				
Zfp146	2.14				
Zfp157	3.17	3.32			
Zfp185		4.49			
Zfp189	7.41				
Zfp212	2.23				
Zfp251	2.43	2.03			
Zfp260	2.03				
Zfp263	2.34				
Zfp27	4.52				
Zfp276		2.44			
Zfp28	5.55				
Zfp281	2.96				
Zfp324		4.33			
Zfp354c	2.63	3.46			
Zfp358	2.11				
Zfp383	5.81	2.13	2.28		
Zfp39	3.87				
Zfp408	4.07	2.22			
Zfp410	2.55				
Zfp449	2.31	3.20			
Zfp462	2.35	3.33			
Zfp51	2.92				
Zfp516	2.46		2.13		
Zfp521	2.60	3.16	2.84	2.63	
Zfp553	2.94				
Zfp566	2.00				
Zfp568	3.44				

Zfp574	3.37				
Zfp58	3.67				
Zfp592	2.36				
Zfp597	2.78				
Zfp606	2.52				
Zfp608				4.27	
Zfp609	2.11				
Zfp612	3.79				
Zfp618		2.21		4.96	
Zfp628	2.32				
Zfp655	2.22				
Zfp667	3.19				
Zfp703	2.03				
Zfp707		2.40			
Zfp711		3.03			
Zfp715	2.47				
Zfp72	4.03				
Zfp747	5.92				
Zfp760	2.14				
Zfp768	2.55				
Zfp772	3.48				
Zfp777	2.09				
Zfp780b	2.21				
Zfp790	4.43				
Zfp799	2.17				
Zfp81	2.00				
Zfp810		5.26			
Zfp821	3.35		2.16		
Zfp827	2.71				
Zfp839	2.05	3.69			
Zfp84	3.01				
Zfp846		2.02			
Zfp868	3.86	3.11	3.07		
Zfp870	6.01				
Zfp871	2.55		2.17		2.85
Zfp874a	2.51				
Zfp874b	4.46				
Zfp933	2.04				
Zfp942	2.24				
Zfp951	4.94				

Zfp952	2.04		
Zfp955a	7.76		
Zfp955b	2.75		
Zfp958	2.27		
Zfr2	2.22		
Zfyve1	2.71		
Zfyve26	2.47		
Zgpat	2.22		
Zhx3			2.29
Zkscan14	3.54		
Zkscan5	2.13		
Zkscan6	2.51		
Zmym4	2.30		
Znrf1	2.42		
Znrf3	2.24		
Zscan25	5.73		
Zscan29	2.12		
Zw10		2.15	
Zxdb	2.09		
Zzef1	2.09		

Table S7: Upregulated Gene Profile in TFAM;HIF1dPA Mutant versus Control from scRNA-Seq Data. Included genes meet the criteria of having a fold change (FC) ≥ 2 and a p-value ≤ 0.05.

**Mutant Upregulated Genes**						
**Gene Symbol**	**Cluster 1 FC**	**Cluster 2 FC**	**Cluster 3 FC**	**Cluster 4 FC**	**Cluster 5 FC**	**Cluster 6 FC**
Abat	2.10					
Abca6		9.16				
Abca8b		6.14				
Abcb1a	7.25					
Abcb1b	3.00	6.48				
Abcg8	10.13					
Abhd5	2.37					
Acat3	4.73					
Ace		4.10				
Acer2	5.90					
Acer3		2.11				
Acot1		3.70				
Acot12	19.88	18.39				
Acot2		5.46				
Acot7				2.34		
Acvr1c	4.38					
Acyp1		2.88				
Adad1		14.74				
Adam30	6.12	84.84				
Adam32	5.16	12.84				125.81
Adamtsl2	37.16	44.91				
Adcy10	2.82	11.21				
Adgrg2		2.41				
Adgrg3	7.10					
Adgrg6		2.51				
Adgrg7	2.81	35.88				
Adig		5.23				
Adm2	3.97	59.10	3.41			
Aebp2		2.56				
Aen		4.96				
Agbl3		11.07				
Agbl4	5.65					
Agpat3		2.21				
Ahcyl2		2.04				

Ahi1	2.01	2.34				
Ak4		4.43		2.57		24.37
Ak6				2.40		
Ak7		16.09				
Alcam			3.88			
Aldh4a1	4.33	4.47				
Aldoa		2.41		3.10	3.43	
Alk		55.96				
Alpk3		15.26				
Ampd1		76.51		12.08		
Amy1	2.90	3.51	5.01			
Amz1	2.08					
Angptl4	3.89	2.39				
Angptl6	3.82	4.46				
Ank		2.29				
Ankdd1a	33.77					
Ankrd2		15.34				
Ankrd37		5.26		28.12	4.31	
Ankrd42	4.66	9.16				
Anks1b		12.03				
Ano3		15.76		10.38		
Antxrl	3.67					
Anxa3		5.83				
Anxa8		5.09				
Aox2	2.12	8.97				
Ap1s2	2.16					
Apoa1	2.50	21.57				
Apoe	3.99					
Apol6	5.05					
Apol9b	5.42					
Aqp3	19.75					
Arap2		10.41				
Arc	6.23	84.12	10.21	3.16		
Arhgap40	7.97					
Arhgef2		2.61				
Arl4d	4.74					
Arl6ip1	2.07					
Arl9		5.74				
Armc4	20.47	6.11				
Arntl2	2.88	4.78				

Asah2	2.29				
Asb11	21.11				
Asb14		26.21			
Ascl4	11.42				
Asns	3.07	3.79		4.22	4.32
Ass1	4.79	14.68			33.67
Atcayos		44.78			
Atf3		3.02	2.25		
Atf4		2.30		2.25	
Atf5	2.64	7.84	2.87	3.15	10.93
Atox1	2.18				
Atp5k	2.01				
Atp5mpl				2.09	
Atp6v0a4		7.95	2.52		
Avil		11.02			
Axdnd1		28.05			
B230206L02Rik	2.88	12.86			
B230369F24Rik		12.62			
B3gnt6	10.18	19.31			
B4galnt2	18.89	66.51			
B530045E10Rik	5.25				
B830012L14Rik		2.61			
B930036N10Rik	3.95		4.78		
BC006965		5.59			
BC023719	35.34	34.46			
BC026762	28.91				
BC048602	31.59				
Baalc	2.00				
Baiap2	3.38				
Barx1	2.11	6.94			
Barx2				9.26	
Bax		3.82		3.13	
Bend6	2.43	3.64			
Bicdl2	6.12	46.08			
Bmp2				2.73	
Bmp6		2.78			
Bmp7		8.52			
Bmp8a	4.63	9.41		18.24	
Bmpr1b	4.34				
Bnip3	2.21	14.48	2.85	5.38	13.77

Bola2		2.24		2.10		
Bpifb2	25.27					
Bpifc	2.38	19.05				
Brca2	8.71	5.75				
Brd2					2.64	
Brip1	4.37	5.80				35.30
Bst2		4.48				
Btbd11	2.02					
Btbd16	4.83	44.36				
Btg1		2.84				
C130073E24Rik	2.51					
C1qtnf9		30.88				
C230038L03Rik		21.68				
C4b	5.46					
C530008M17Rik		2.93				
C920021L13Rik		4.51				
CN725425	12.05					
Cacng5	12.36					
Cadm2	2.05					
Camk2b	2.77					
Car6	52.89	354.47		65.67		
Car8	2.32					
Car9		7.69				
Casc1	2.74	8.50				
Casp12	2.08					
Catsperd	5.05	20.73				
Ccdc13		54.63				
Ccdc138	2.55	5.00				
Ccdc141		3.46				
Ccdc152	6.07	6.71				
Ccdc158	4.97					
Ccdc36	8.17					
Ccdc58		3.56				
Ccl2	13.51			27.70		
Ccl5	60.18					
Ccl7	26.13			2.67		
Ccne1				29.07		
Ccng1		5.22				
Ccnyl1	2.41				3.34	
Cd109		2.84		2.13		

Cd247	7.67					
Cd44				2.17		
Cd68		5.32		9.91		
Cd79a		2.27				
Cd80		38.11				
Cdkl4		8.10				
Cdkn1a	2.44	12.22	2.36	5.64	8.21	
Cdsn	107.94	41.23				
Cebpb		5.12		3.02		
Cebpg		2.04				
Cenpw	3.13					
Cfap43	2.81					
Cfap61	19.90	13.95				
Cfap70	4.33	6.84				
Cfh	5.42					
Chac1			6.19			
Chchd10	10.05	37.35		34.52	14.17	303.60
Chchd7	2.14					
Chil1	2.60					
Chka		4.01			2.84	
Chrdl2	7.42					
Ciao2b				2.14		
Cited2	2.06					
Ckap2		10.20				
Ckm	35.57					
Cks2				4.84		
Cldn11	5.78	9.35				
Cldn20	3.58					
Clec3a		6.11				
Clhc1	8.83	12.69				
Cmc1		2.07				
Cnmd		9.28				
Cnnm1	13.56					
Cobl		2.96				
Col11a2		6.03		4.22		
Col20a1	2.18	5.01				
Col2a1		8.54		6.02	3.82	
Col4a4	4.65					
Col4a5	3.92					
Col4a6	4.79					

Col9a1		7.84				
Col9a2		5.67				
Col9a3		6.54				
Colgalt2		8.23				
Corin	9.32					
Coro7	4.98	3.01				
Cox17		3.15		2.14		
Cox4i2		7.82		3.92		
Cox6a2		65.99		90.68	151.10	
Cox6b2		2.93				
Cox7a1				18.13		
Cox7a2				2.02		
Cox8a		2.02		2.03	2.04	
Cpe		3.96				
Cpeb2		4.02				
Cpne8		4.57				
Cpsf4l	6.88	10.50				
Crb1	14.52					30.19
Creb5		3.21				
Crim1		2.13				
Crispld1		2.74				
Crnde	4.21					
Crtam		8.34				
Cryba1	9.95					
Crybg3		2.50				
Csf1r	4.45					
Csgalnact1		7.53				
Cspg5		4.02				
Csrnp1				2.20		
Cst6		10.20				
Cstb		2.82				
Ctcflos	5.02					
Cth		7.31				
Ctsd		2.05				
Cx3cr1	5.61	21.63		12.96	12.75	
Cxcl1		6.30		11.09		8.78
Cxcl10	26.34					
Cyb561		5.59				
Cyb5r1	2.75	5.72		5.10	5.68	
Cycs				2.55		

Cyfip2		5.70				
Cyp26b1		4.78				
Cyp2u1	10.76	6.72				
Cyp3a13	2.72	41.21				
Cytl1	5.28	40.73				
D130043K22Rik	87.13	110.76				
D230022J07Rik	5.81					
D430041D05Rik		11.03		9.36		
D530033B14Rik	4.63					
D830025C05Rik	5.12	11.20				
Dand5	4.98	6.07		4.76		
Dbi	2.22	3.03				
Dbn1	3.20					
Dbpht2	10.17					
Dct	36.39					
Dctn3	5.26					
Dctpp1		2.61				
Dcx	2.07	12.55				
Ddit3		5.42		2.66	3.92	
Ddit4		3.19		3.51		
Ddit4l		4.40				
Ddr2	2.72					
Ddx27		2.46				
Dglucy		5.47				
Dhcr24	2.26					
Dhrs9	2.17	12.94				
Dhx58os		3.41				
Dlgap1		10.98	2.35			
Dlk1		2.32				
Dmkn	7.16					
Dmrta2		12.16				
Dnah12		33.81		66.21		
Dnah2	5.53					
Dnaja1					2.31	
Dnaja3	3.00	3.76				
Dnajb1		2.13			2.47	
Dnajb9				2.19		
Dnajc12	2.76					
Dnajc15		2.45				
Dnajc6		10.35				

Dnmt3l		81.54				
Dock5	2.25					
Drd3	34.92	110.70				
Dupd1	6.98	29.39				
Dusp5	2.67	3.29	2.60			
Dync2li1	2.27					
Dynlt1f				2.04		
Dysf	2.19					
E030030I06Rik		12.53				
E130008D07Rik	23.41					
Eda2r		20.55		6.61	28.31	
Ednra	4.96					
Efcab8	7.77	29.85	10.13	24.78		
Efna2	2.77					
Efnb2	2.18					
Egln3		5.09			6.90	
Egr1		2.41	2.27			
Egr3		3.21		2.95		
Eif1		2.42		2.23	2.48	
Eif3c		2.48		2.06		
Eif4ebp1				2.06		
Elavl3		52.51				
Ell2				3.41		
Eml6	2.03					
Eno1		5.33	2.02	7.06	7.53	5.78
Enpp2	3.52					
Epas1		3.60				
Epb41l4aos		4.22	2.24			
Epb41l5	2.28	5.62				
Epcam	3.10					
Ephx1	2.18	8.42				
Epm2a	3.35					
Eprs					2.15	
Eps8		2.46				
Eps8l2		6.12				
Erich2	5.11					
Ermap	22.32					
Ero1l		4.65		4.14	5.44	
Errfi1		2.31				
Esd		2.86		2.17		

Esm1				92.63		
Ets2		2.92				
Exoc4		2.62				
Extl1		6.44				
F13a1	13.09	12.94				
F630040K05Rik	2.28					
Faah	4.89					
Fabp4	77.76	73.77				
Fads3				2.88		
Fam162a	2.42	10.16	2.31	8.06	4.42	6.85
Fam166a		20.46				
Fam169b	10.31					
Fam177a	2.52	3.74				
Fam186b	6.06					
Fam20c		3.93				
Fam222a	4.66					
Fam25c	3.29					
Fam49a	3.99					
Fam71a		14.71				
Fam71d	6.62	22.36		23.46		
Fam71e2	8.50	36.00				
Fam71f2		17.14			40.21	
Fam83g	2.85					
Fam89a	2.07					
Fanci	6.34					
Fank1	2.65					
Far2	14.70					
Fau		2.09				
Fbln5	3.89					
Fbxo47	2.53	9.05				
Fermt3	3.10	7.67				
Fgl1	3.47	21.56				
Fhad1	3.51					
Fhl2				3.07		
Fhl5	11.06					
Flg	35.68					
Flt1	4.30					
Fmc1		2.17				
Fmo5	3.57					
Fmr1nb	25.65	52.51				

Fn1		2.11				
Fnip2		2.05				
Fosb		2.01	2.39			
Fosl1		4.53		11.36		
Foxd1		18.00				
Fry		4.91				
Frzb		2.31				
Ftcd	8.31					
Fth1		2.13				
Ftl1		3.82		2.09	2.58	
Fxyd2			2.39			
Fxyd4		6.78				
Fzd5	2.56					
G530011O06Rik	25.98	18.50			28.28	
G930009F23Rik	5.47	19.65				
Gadd45a		6.88		2.32		
Gadd45b				5.14		
Gal	74.05					
Galnt18		2.01				
Gapdh		2.13		3.50	2.96	
Gas5		3.64		2.27	3.71	
Gbe1		2.66				
Gcg		42.50				
Gch1		6.17		2.72		
Gclc		4.89		3.88	8.38	
Gdf15	35.77	69.56		13.81	5.78	
Ghitm		2.50				
Ghsr	11.70	22.88				
Gipc2	3.80	10.49		21.44		
Gjb4	7.54					
Glce		2.25				
Glis1		4.24				
Glra1	13.41	97.68				
Glrx	2.38					
Gm10000	6.35					
Gm10076	2.07	2.33			2.03	
Gm10260	119.68	169.93	20.54	77.34	22.54	108.52
Gm10371		7.56				
Gm10432						29.59
Gm10610	281.62					

Gm10635	5.62	10.44				
Gm10648	2.81					
Gm10851	3.27					
Gm10863	16.26					
Gm11077		21.24				
Gm11110	2.25	4.88				
Gm11290		5.93				
Gm11339	12.19					
Gm11361	5.52	8.33				
Gm11638	4.22	9.85				
Gm11808	2.66	2.94	2.70	2.58		
Gm11906	4.52					
Gm12227	3.93	9.68				
Gm12236	6.98	42.03				
Gm12248		7.69				
Gm12295	27.72					
Gm12299	77.50					
Gm12353		2.95				
Gm12506	11.55					
Gm12703		26.52				
Gm12724	4.53					
Gm12743	2.74					
Gm12940	3.56	4.79	3.57		3.72	
Gm13274	6.42					
Gm13853		20.38				
Gm13943	25.72					
Gm13944		10.17				
Gm14051	4.70	5.00				
Gm14168	2.52					
Gm15247	12.14	15.13				
Gm15345	4.51			11.68		
Gm15353	4.21	53.95				
Gm15533	15.02	32.24				
Gm15559		5.07				
Gm15614		2.53				
Gm15675		8.32				
Gm15726	47.21	186.59		21.77	79.55	22.48
Gm15728				19.15		
Gm15834		7.67				
Gm15867	2.32					

Gm15892		11.06		18.90	
Gm16045	4.19				
Gm1604a	6.03				
Gm1604b	6.93				
Gm16062				5.56	
Gm16070		3.75			
Gm16079	2.65	23.25			
Gm16093	3.07				
Gm16133	2.58	18.38			
Gm16145	6.77				
Gm16150		19.97			
Gm16157	15.31	25.36			
Gm16178	4.08	10.59			
Gm16268	2.73	16.65			
Gm16310	2.97	3.26		4.59	
Gm16564	6.92				
Gm16596	6.98	27.33			
Gm16638	3.51	10.57	3.62		
Gm16754	5.10	22.82		19.49	
Gm17056	4.78	33.80	8.49	11.25	
Gm17103	5.25				
Gm17173		13.74			
Gm17268				21.02	
Gm17333	32.18				
Gm17359	3.60	10.87			
Gm17484	4.07	12.73			
Gm17749	13.44	26.88			
Gm19325		29.26			
Gm19510	4.20				
Gm20597	8.35				
Gm20735	7.19				
Gm21691	12.03				
Gm21859	3.42	8.55			
Gm21860	14.43			3.51	5.95
Gm21887	2.72	3.87			
Gm26532		2.64		2.52	4.09
Gm26670		9.23			
Gm26802	5.42	32.47	16.66	3.67	
Gm26812	4.54	55.21			
Gm26917		4.47			

Gm28055		13.32				
Gm28153		13.27				
Gm28198	3.46	10.35				
Gm28376	3.96					
Gm28403	5.47	6.75		8.06		
Gm28535	23.71					
Gm2895	5.23					
Gm29050	10.32	19.46				
Gm29282	3.56					
Gm29570	2.86					
Gm29571	11.60	20.63				
Gm29681	3.79	19.45				
Gm29966	101.51	69.86				
Gm30363	4.21					
Gm30551	9.88					
Gm31508	5.89					
Gm31641	6.75					
Gm31718		2.92				
Gm32592						32.60
Gm3331	5.26					
Gm3336	7.45	216.12		13.76		
Gm33508	11.72	46.70				
Gm34030	3.64					
Gm34455	4.18	16.78	5.44	12.91		
Gm34471	2.46					
Gm3448		7.70				
Gm35019	6.88	21.58				
Gm35248	6.61					
Gm36447	5.86	12.38				
Gm36862	18.32	30.78				
Gm37245	6.53					
Gm3734		35.64				
Gm37359	6.77					
Gm37459		15.71				
Gm38155	15.99					
Gm38575		76.74				
Gm40689		35.08				
Gm41409		14.71				
Gm41496	12.51	32.31		42.59		
Gm41505	4.98	18.05				

Gm41556	7.96	22.99		9.43		
Gm41609	5.07	19.34				
Gm41790	7.76	25.42				
Gm41819	74.31					
Gm42047	4.76	6.36		3.94		
Gm4211		15.28				
Gm42196		41.21				
Gm42477	3.35					
Gm42529	25.72					
Gm42531	11.80					
Gm42743	80.67	38.76	48.24	45.00		
Gm42912	24.13	24.90				
Gm42951	47.11	199.14	9.26			
Gm43063		27.23				
Gm43660		13.80				
Gm44127	2.81					
Gm44145	14.45	66.79				
Gm44284		14.63				
Gm44956		24.49				
Gm45075	7.85	22.99				
Gm45518	11.46	24.06				
Gm45570	11.31					
Gm4566		32.55				
Gm47095	36.27	35.79				
Gm47123	8.12					
Gm47128	49.44					
Gm47283	2.67	3.43	2.78	2.98	2.32	
Gm47416	9.70					
Gm47507		2.64				
Gm47818	38.94	33.77				37.24
Gm47868	31.78					
Gm48024	7.88	42.79		13.11		
Gm48094	13.72	39.03				
Gm4869		9.63				
Gm49085		8.92				
Gm49173	3.24	9.50				
Gm49267	4.08					
Gm49454	7.38	36.72		10.98		
Gm49519		12.55				
Gm49622		103.65				

Gm49735	11.83	297.19		13.13		24.70
Gm49774	6.67	12.96				
Gm49959		2.99				
Gm50186	14.79					
Gm50225	8.34					
Gm50333	5.57	53.38				
Gm5099	7.43					
Gm5444	7.43	37.18	7.11			
Gm553	5.32	6.28				
Gm6225		7.78				
Gm6994		11.38				
Gm8251	4.14	12.90				
Gm826	14.38	9.45				
Gm867				79.47		
Gm9828		3.04				
Gng3		3.42				
Gng5		2.45		2.21		
Got1		2.82				
Gpc6		3.66				
Gpi1		3.33		2.64	3.09	
Gpr137c	8.08	8.31				
Gpr39	11.38					
Gpr84	14.56					
Gprc5a	7.70	50.75		4.72		
Gprc5d	43.70					
Grem1	4.30					
Grhpr						7.92
Gria3		2.49			43.53	
Grid2	3.14					
Grik4	3.18					
Grm4	30.78					
Gss		3.24				
Gsta4						50.24
Gstm1		4.80				
Gsto2	5.45	11.45				
Gstp1	2.95	4.72		2.77		
Gstp2		23.09				
Gtf2b	3.01					
Gtse1		18.80				
H2-Ab1	3.19					

H2afj	2.11	3.46				
H2afx	2.54					
H2afz				2.29		
H3f3b		2.47				
Hal	6.85					
Hand2	5.02					
Hand2os1	2.96					
Hao1	19.82	19.84				
Hapln1		3.86				
Has1		4.86				
Has2os		3.87				
Haus8				4.84		
Hbegf	2.18	8.14		4.21		
Hecw1	26.14	18.94				
Hes1				3.14		
Hexim1					4.08	
Hfe	2.38					
Hhip		2.18				
Hhipl1	3.16					
Higd1a	2.69	7.83	2.77	6.43	4.42	5.49
Hilpda		2.00				
Hint1				2.13		
Hip1r		2.98				
Hist1h1c		3.90				
Hist1h1t	24.11					
Hist1h2ac	3.05	14.95	4.96			
Hist1h2ae	10.04					
Hist1h2ap	4.69					
Hist1h2bc	2.79	3.49	3.05	2.97		
Hist1h2be	4.94	5.12	5.05			
Hist1h2br	4.82					
Hist1h3d	7.54					
Hist1h4d	3.39	6.04	2.88			
Hist1h4h		8.84				
Hist1h4i	3.15	7.20				
Hist2h2bb	27.96	9.94				
Hist2h3c1	5.08		7.16			
Hk2		2.98	2.07	3.39	7.57	
Hmbs	3.97					
Hmcn2	2.38					

Hmga1	2.54	9.86		9.90		
Hmga1b	5.50					
Hmga2		4.64				
Hmgb2	2.05	2.69				
Hmgcr		2.92				
Hmox1	4.75	7.89		5.55		
Homer1		4.44		4.21	4.32	
Hormad2	8.44	28.09				
Hotairm1	3.29	4.37				
Hoxd3os1	3.24					
Hoxd8	2.90					
Hoxd9	2.60					
Hpgds		4.96				
Hprt				2.03		
Hsd17b1		7.60				
Hspa1a	2.11					
Hspa1b	2.10					
Hspa2		2.70			3.87	
Hspa9		2.95			2.47	
Hspb1		2.46				
Hspe1				2.14		
Ica1		2.91				
Id4		5.83				
Idi1	2.14					
Ier2					2.30	
Ier3		2.57		4.04	3.01	5.76
Ier5		3.99		3.11		
Ier5l		2.54		2.60		
Ifi202b				4.92		
Ifi203		7.50				
Ifi207		3.68				
Ifitm10		3.50				
Ifitm3	2.95					
Ifrd1		2.30		2.20	2.39	
Igf2bp3		3.22				
Igsf9b		18.88				
Il10	4.09	24.90				
Il12rb2	8.89	7.48				
Il1rapl1		6.93				
Il27	5.07	9.62				

Impg2	5.74	3.31				
Inka2		14.24				
Inpp4b		5.70				
Insl6	3.95	16.67				
Ints6		2.27				
Ip6k3	2.88					
Ipmk	2.16					
Iqcn	18.31	63.08	23.97	5.10		
Irs2		2.73				
Irx3	2.14	5.98		7.40		
Irx5	7.78	5.16		12.28		
Isg15		3.43				
Isg20		2.92				
Itga2	6.35					
Itga6		3.73				
Itgam	5.98					
Itgb4	5.22	6.36				
Itgb6	9.07	9.75	6.31	31.41		
Itpr3		3.98				
Jade1		3.68				
Jam2		2.78				
Jhy	19.90	45.53				
Jmjd6				2.45		
Jph1	3.69					
Jpx		3.23				
Junb				2.76		
Kansl1l		2.10				
Kcna6		6.84				
Kcnip4	5.39	5.95				
Kcnj6	100.72	43.80				
Kcnq5		5.30				
Kcnrg	4.11	4.81	3.36			
Kcnt2	21.91					
Kdm3b	3.26					
Kdm6b		2.37		2.32		
Kif17	5.57	44.30				
Klb	15.66					28.46
Klf2			2.22			
Klf4		3.59		2.14	3.46	
Klhl10	5.32					

Klhl21		3.56				
Klhl40	4.10	26.68				
Klhl8	3.84					
Klk10	7.73					
Krt16	82.04	174.71	8.40			
Krt17	50.07					
L3mbtl1	3.83	33.13				
Lag3		4.03				
Lamtor3				2.03		
Larp1b					3.23	
Lax1		4.28		15.29		
Layn	2.09					
Lbh	2.07					
Lbhd2		5.60				
Lct		22.98				
Ldha		3.79		4.39	4.07	4.16
Ldlrad2		9.87				
Lef1		3.16				
Lekr1	4.01					
Letm2	2.37	5.94				
Lexm	30.45					
Lgals3		2.02				
Lgals7		10.20				
Lgr6		3.39				
Lhfpl3	4.36					
Lias	4.07					
Limch1		2.92				
Lims2		10.75				
Lix1	3.05					
Lman1l	2.58					
Lmcd1	3.18					
Lmntd1	10.22					
Loxl4		12.91				
Lpar4		2.73				
Lrp8os3	4.11					
Lrrc15	16.09					
Lrrc28	2.16	3.48				
Lrrc43		25.40				
Lrrc51		4.63				
Lsm8				2.04		

Lsmem1	14.69	49.34	25.71	9.01	6.44	36.61
Ly6c1	7.79					
Ly6m	482.74					
Lyn	3.80	2.42				
Lypd3	20.88					
Mafa	2.50					
Maff				2.85		
Mafk		3.43				
Mak16		2.26				
Malat1		2.46				
Mall		5.89				
Manba	3.03					
Map3k12	2.31					
Map3k15	6.28	10.17				
Map3k19		3.97				
Map3k20		4.32				
Map7	4.75	9.90				
Map9	3.76	5.21				
Mapk9	2.62					
Mapkbp1	2.04					
Mapt	4.60	16.52				
Masp2		28.25				
Mb	8.42	51.07		23.49		
Mc5r	4.23					
Mcf2l	5.87					
Mcmdc2	5.39	14.73				
Mdc1	2.88					
Mdm2		12.86				
Mecom	2.14					
Med12l	3.21					
Mef2a		2.31				
Meis2	2.59					
Meltf		9.28				
Mep1b	12.38	68.54				
Mfsd4b5	32.46					
Mgarp		29.59	2.84	94.98		32.25
Mgmt		5.52				
Mgp			2.99			
Mia		4.18				
Mical2	3.20					

Mid1	6.29					
Midn					2.84	
Mif		4.81		8.01	7.21	8.40
Milr1	14.78	36.77	6.43	4.72		
Mir155hg	7.80	14.14	13.57	5.49		
Mirg		2.61				
Mknk2	2.54	2.56			3.24	
Mlf1	3.17	5.16				
Mlph	6.78					
Mmp25	3.80	16.79				
Mmp28	2.91					
Mmp9		2.09				
Mmrn2		36.72				
Mocos	4.77					
Morn1	3.43	4.05				
Morn3	8.97					
Moxd1		15.98	2.74			
Mpp4	2.85	16.91				
Mreg		17.15				
Mrnip	5.15	51.90		11.54		
Mrpl33	2.18					
Mrpl52		2.04		2.18		
Mrps18c		2.07				
Mrps6		2.82		2.37		
Msh4	2.87	8.17				
Mss51		3.16				
Msx1	2.16					
Mt1	2.26	6.62		3.33	3.09	
Mt2	2.99	5.47		3.37	3.94	
Mtfr2	3.19	15.56				
Mthfd1l		7.51	2.41			
Mthfd2				2.16	8.22	
Mtif2		2.14				
Mtln		2.66				
Mtmr7		10.58				
Mtnr1a		70.75				
Mtnr1b		48.43				
Mttp		7.55				
Muc19	25.73					
Mup5	22.88	174.10				

Musk	7.85					
Mybbp1a		2.11				
Mybl1		7.27		5.77		
Mybph	9.84					
Myc		3.30		2.46		
Mylip	2.10					
Mymk	12.94					
Myo15		8.14				
Myo16	2.68					
Myo18b	6.98					
Myo3b	10.62	72.86		27.15		
Myo5c	5.88	22.04				49.92
Myo6			2.01			
Myocd	5.41					
Myocos		29.00				
Myom1	2.66					
Myoz3	3.48	9.25				
N4bp2l1		4.08				
Nabp1	9.36	3.45			10.87	
Nars		2.10				
Nckap5		4.02				
Ncl		2.08				
Ncmap		8.10				
Ndrg1		3.12		2.24		
Ndufa11	2.07					
Ndufa3	2.40					
Ndufa4l2		15.92		7.25	2.35	38.48
Ndufa5	2.11	2.04				
Ndufa7		2.08				
Ndufaf8		2.28				
Ndufb6				2.38		
Ndufc1	2.12					
Neb		14.34				
Nebl		3.90				
Nedd4l		5.06				
Nek10	10.33	171.88	14.17	14.94	37.45	61.33
Nek11	3.27	15.04				40.99
Neu2	3.71	11.61	3.13			
Neurl1a	4.82					
Nfasc		3.23				

Nfatc1		3.06			
Nfe2l1		2.40			
Nfe2l2		2.44			
Nfe2l3	6.64	32.91	17.98		
Nfkb1				2.42	
Nfkbia		2.60		4.89	
Nfu1		2.56			
Nim1k	2.78	5.86			
Ninj1		3.12			
Nipal1	4.08				
Nkx6-1		20.23			
Nol8		2.41			
Nop10		2.36			
Npas3		5.85			
Nqo1		13.82			
Nr4a1		3.10		2.59	
Nr4a2				2.79	
Nr4a3				3.77	2.32
Nrg4		23.33			
Nrip2		8.61			
Nrn1	2.57				
Nsa2					2.51
Nsun7	8.07	6.05			
Nt5e		3.58			
Nup210l	2.21	7.19			
Nupr1				3.35	6.65
Nxph4		16.08		8.88	
Nyx	6.38				
Oacyl		17.31			
Oca2	7.37	33.67			
Odc1	2.11				3.11
Oit3	7.45				
Olfr267		42.01			
Olfr920		7.12			
Orc1		6.48			
Orm2	30.80				
Osr2	2.17	7.02			
Ost4				2.29	
Ostn				24.46	59.13
Otulinl	3.68				

Oxnad1	3.22					
P4ha1		2.61	2.39	2.51		3.94
Pabpc1l		8.04				
Pacrg	2.13	2.97				
Padi2	10.16					
Pafah1b3				2.54		
Paip2b		2.52				
Pam16		2.42			2.10	
Pappa		3.95				
Papss2		2.78				
Paqr8	3.10					
Pask	7.65					
Patl2	7.64	30.03				
Pcbd2		2.31				
Pcdh11x	2.11	14.75				
Pcdh17	9.48	26.40	13.39	19.70		
Pcdh19	7.44					
Pcf11		2.59				
Pcgf5		2.38				
Pcsk5	2.43					
Pcx		5.45				
Pdcd1	4.53					
Pde4c	6.03	119.36		17.17		
Pdgfd	3.20					
Pdk1		3.48		5.21	4.39	
Pdk4		10.02				
Pecam1	20.87	53.23	12.75	18.89	5.00	19.60
Penk	2.05					
Perp	3.84	14.31				
Pfdn2		2.10				
Pfkfb3		2.55	2.26			
Pfkl		4.38		5.09	4.74	6.93
Pfkp		2.08				
Pgam1		2.63		3.88	2.82	
Pgd		3.24				
Pgk1		4.67		5.33	5.68	4.89
Pgpep1l	7.25	14.82		8.75		
Pgrmc2		2.00				
Phex	3.78	2.62				
Phgdh					12.74	

Phkg1	8.06	13.51	28.05		
Phlda1		4.96		2.69	
Phlda3		2.87			
Piezo1		2.80			
Pigf		2.47			
Pik3r3	3.85				
Pim1				2.04	
Pim3				2.96	
Piwil2		4.55			
Pkm				2.56	2.37
Pla2g12b	35.10	6.04			
Pla2g4a	2.36				
Plac9b	2.36	4.79			
Plaur				2.60	
Plcb1		2.67			
Plcd4		11.56			
Plch2	16.87	21.83			
Plet1	2.61				
Plk2	2.14			2.46	
Pmaip1		13.92			
Pnldc1	3.44				
Pnp2	17.52	10.52			
Pnrc1		2.82		2.36	2.27
Podn		3.20			
Polg2	3.90	12.07			
Polr2a	2.91	3.19			
Polr2k		2.31			
Polr2l	3.13	2.69	2.70		
Polr3b		2.79			
Pomc		6.06			
Popdc3	6.78	59.38			
Pou2f3	6.33				
Pp2d1		9.50			
Ppef1	27.53				
Ppm1l	2.46				
Ppm1n	10.03	177.24			
Ppp1r10		2.71		2.35	
Ppp1r11				2.03	
Ppp1r15a		3.44		2.18	
Ppp1r1b		33.87			

Ppp1r1c	22.28	19.32				
Ppp1r36		11.65				
Prelid1				2.42		
Prelid2				4.67		25.05
Prg4	3.57	15.35			24.50	
Prkcg		8.77				
Prkg2		26.61				
Prlh	4.59					
Prnd		23.79				
Prnp		3.26				
Procr		17.01				
Prpsap2	3.74					
Prrg4		58.13				
Prss22	17.76					
Prss27		15.16				
Prtn3	10.10					
Psat1	2.68			3.42		
Psmg4		2.17				
Psrc1		32.12				
Pth2r	19.64	40.03				
Ptprb				13.47		
Ptpre		3.27				
Pvr				2.31		
Pvt1		4.25				
Pycard	4.84	3.76				
Pygm		4.17				
R3hdml		6.25				
Rab11fip4		5.07				
Rab11fip4os1		16.59				
Rab26os		3.25	3.31			
Rad23a				2.00		
Rad54b		4.42				
Rapgef4		2.41				
Rasgef1c	19.65	34.51		64.50		
Rbm38					2.97	
Rbm44	9.78	34.35				
Rbpjl	6.57					
Rcan1		4.57				
Rcc1	4.46	2.11				
Rcc2		2.10				

Rec114	3.40	8.32			
Reep2	10.10				
Reg4	12.74	74.78			
Rgcc	2.76			3.11	3.88
Rgma		2.88			
Rgs20		9.89			
Rgs5	67.62				
Rhbdf2		4.89			
Rhob	2.89	2.14	3.34	2.28	
Rnf19b					2.46
Rnps1				2.16	
Rpl10					2.20
Rpl18a					2.05
Rpl22		2.11			2.10
Rpl23		2.02			2.27
Rpl26					2.01
Rpl30		2.13			2.24
Rpl32		2.18			2.28
Rpl34		2.16			2.02
Rpl35	2.01	2.47	2.15		2.25
Rpl35a		2.24			2.10
Rpl36		2.22			2.03
Rpl36a		2.13			
Rpl37		2.28			
Rpl38		2.14			
Rpl39		2.15			2.07
Rpl39l				6.00	
Rpl41		2.30		2.05	2.07
Rplp0					2.04
Rplp1		2.20			2.51
Rplp2		2.21			
Rpp21		2.02		2.25	
Rps10					2.21
Rps12					2.47
Rps15a					2.05
Rps17					2.07
Rps19		2.38			2.30
Rps2					2.20
Rps20					2.27
Rps23					2.12

Rps24		2.02			2.62	
Rps26		2.39			2.26	
Rps27		2.48	2.12		2.36	
Rps27l	2.15	3.48		2.94	2.85	
Rps28	2.05	2.47	2.01		2.30	
Rps29	2.12	2.52	2.33		2.36	
Rrm2b		5.69				
Rs1	6.29	25.47				
Rsrp1		2.23		2.02		
Rtn1	7.77					
Rtn2	12.64	63.79	19.98	10.24		
S100a10				2.17		
S100a2	3.37					
S100a3	5.19					
S100a6	2.41					
S100b		2.12				
Samd12	3.77	26.40				
Sars		2.90		2.45	3.20	
Sat1		2.62				
Scgn	13.83	92.94				
Scin	6.13	37.27		44.51		38.16
Scn4a	17.02					
Scrg1		7.11				
Sdc4		2.11			2.51	
Sdk2		2.78				
Selenok-ps1	4.40					
Sema3d	2.29					
Sema3e		2.25				
Sema4a	5.89	24.65				
Sema6b	3.97					
Serpinc1		7.62				
Serpine1	2.66	2.43	3.20	9.55		
Serpine2	2.34	2.12				
Serpine3	4.82	30.15				
Serpini1	4.46	5.25				
Sertad1		3.94		3.00	6.24	
Sertad2		2.40				
Sesn2	3.25	5.94	3.32	4.88		
Sfn	10.28	8.78				
Sgo2b	10.00					

Sh2d4a	3.24					
Sh2d4b		6.42				
Sh2d6				5.32		
Shmt2					4.36	
Sim1	69.40	52.24				
Simc1	2.06	2.65		2.30	4.88	
Siva1		2.22				
Skap2		2.20				
Skil		2.02				
Slc10a1	2.45	8.07				
Slc12a8	7.54	5.81				
Slc13a4		26.66				
Slc16a10	4.40					
Slc16a3		3.72		6.50	3.60	6.22
Slc17a4	61.59					
Slc19a2		8.76				
Slc1a5		2.14				
Slc20a1		5.60		2.66		
Slc22a1		17.83				
Slc22a14	2.45	9.27				
Slc22a2	15.09					
Slc22a4		6.28				
Slc24a3		3.69				
Slc25a33		3.73				
Slc25a34		29.48				
Slc29a1		2.24				
Slc2a1		7.37		5.39	5.60	
Slc38a3		8.66				
Slc43a3		3.30				
Slc45a1	9.90	32.73				
Slc48a1		3.53				
Slc4a5		28.09				
Slc4a7		2.40				
Slc5a10	8.53	30.76				
Slc5a11	8.37					
Slc5a3		2.65				
Slc6a20a	13.62					
Slc6a20b	4.08					
Slc6a9	2.57	3.86				
Slc7a11		2.76				

Slc7a2		3.52			
Slc7a3	7.45	11.92	9.37		
Slc7a5		3.28		3.16	11.77
Slc8a2		10.40			
Slco1b2	14.69	33.41			
Slco1c1	5.67	13.56	4.72		4.83
Slpi	4.74				
Slurp1	17.12	47.95			
Smco2	5.05	43.45			
Smim26		2.41			
Smim27		2.05			
Smim4	2.11				
Smoc1		3.07			
Smoc2	2.06				
Sms		2.75			
Smu1	2.08				
Snhg1		3.23		2.70	3.26
Snhg12		3.92		2.56	
Snhg15		9.55		4.73	3.92
Snhg3	2.45		2.04		
Snhg6		3.83			
Snhg8	2.25	3.97	2.65		
Snorc		11.73			
Snrpf				2.18	2.37
Soat1		2.36			
Soat2	7.15	9.87			
Sod2		2.04			
Sod3	2.10				
Sorbs1		2.98			
Sorbs2		2.06			
Sox10	26.18				
Sox5		2.68			
Sox6		2.02			
Sox9		3.98			
Sp3os		4.26			
Spag1	3.32	4.56			
Sparcl1		4.71			
Spata1		3.05			
Spata32	3.81				
Spatc1l	17.25				

Spc24	3.04					
Spp1	7.76	3.78				
Sprr1a	144.90					
Spry2		3.28				
Sqstm1		2.92		2.31	3.17	
Srxn1	2.22					
Ss18l2		2.55				
Sstr2	4.55					
St14	13.00	47.70				22.12
St3gal6		2.83				
Stab2	7.91	49.95				
Stard5	2.50					
Stat1	2.54					
Stbd1		16.48		20.08		
Stc2	2.22	2.98				
Steap1		5.82				
Stfa2	7.55					
Stk10		5.66		3.61		
Stk26		3.77				
Stk31	9.87					
Stk32b	2.80					
Stmn1						6.54
Stpg4	6.09	28.46	8.26	4.08		
Strip2	5.69	26.83				
Sugct	2.22					
Svop		72.72				
Svopl	5.45	66.03				
Sycp3	9.30					
Syn1	8.66	4.15	3.36			
Syn3	2.06					
Synpo2		4.33				
Syt2	3.41	74.92				
Sytl1	5.21	20.84				
Taf1d		2.27				
Taf4b	2.45	3.06	2.47		3.43	
Tafa4	13.11					
Tas1r2	13.12					
Tbpl1		2.45				
Tcp11	3.72					
Tek	11.58					

Tenm4	13.64	6.72		12.02	13.38
Terb2	50.06	39.86			
Tesk2	4.21				
Tesmin	6.08				
Tex14	4.63	10.32	9.74	2.98	
Tex35		16.48			
Tfap2b	24.96				
Tfb1m	7.57	3.67			
Tfcp2l1	5.55	21.39			
Tfrc		4.34		5.97	4.37
Tgfa		7.32			
Tgif1		2.77		2.23	
Thbs4	2.65				
Thsd1		5.31			
Thyn1		2.59			
Tigit	3.62	47.74			
Timp1	3.10			3.44	
Tiparp				2.29	
Tjp2				2.55	
Tktl1	6.35				
Tlcd2					5.56
Tll2	11.58	2.38			
Tm4sf1	3.92				
Tm4sf4		34.19			
Tmc3	15.77	22.43			
Tmc6	2.07				
Tmeff2	26.62				
Tmem116		12.51			
Tmem132b	4.74	17.16			
Tmem163	2.74				
Tmem212		13.74			
Tmem213	2.17	9.11			
Tmem232		9.97			
Tmem236		12.17			
Tmem265		2.82			
Tmem267	6.74				
Tmem30c		10.68			
Tmem88		44.32			
Tmsb10	2.46				
Tmx4	2.34				

Tnfaip3				2.81	
Tnfrsf10b		5.72			
Tnfrsf11b		4.31			
Tnfrsf12a				2.59	3.72
Tnfsf10	19.45	56.83		21.57	
Tnfsf9		3.60		5.81	
Tnnt3		3.93			
Tob1		2.58		2.22	4.74
Tomm6		2.65			
Tomm7		2.26			
Tpd52l1		6.86		9.47	
Tpi1		2.26		3.31	
Tpp1		2.17			
Tpt1					2.05
Trappc6a		2.94			
Treh		31.44			
Trib1		5.11			
Trib3	3.52	44.51	6.31	25.38	79.84
Trim11		2.94			
Trim17	5.64	6.76	8.03		
Trim69		5.83			
Trim7		5.74			
Trim72	10.30	44.55			
Trp53cor1	7.47	9.27			
Trp53inp1		5.17			
Trpm6		3.60			
Trpv4		5.59			
Tsga10	3.55				
Tshr	17.53				
Ttc21a	8.05	265.74		53.85	
Ttc23l	7.36	74.40			
Ttc27		2.72			
Tuba1c		2.05			
Tubb4b	2.03	4.79		2.82	
Tulp1	11.70				
Txn1		2.12			
Txnrd1		2.58			
Uap1					4.03
Ubc		2.39		2.53	2.46
Ube2cbp	2.25				

Ube2s				2.06		
Ube2u	35.13	312.36		46.63		
Ucma		3.68				
Ugdh		3.32			4.01	
Ugp2		2.22				
Ulk2	3.11					
Ulk4	2.94					
Uox	7.56					
Uqcc2	2.12				2.83	
Ushbp1	9.79					
Usp50	2.30	5.22				
Utp20		2.67				
Vegfa		5.48	2.26	3.81	2.98	4.86
Vit		6.28				
Vldlr		2.08	2.38	2.75	3.53	
Vmp1		2.02				
Vps11	2.05					
Vps13b		2.08				
Wdr78	2.92					
Wdr95		13.72				
Wdsub1		2.86				
Wif1		3.06				
Wnt4	2.56					
Wsb1					2.35	
Xirp2	36.34					
Xkr9	21.43					
Xylt1		2.93				
Ybx3					2.34	
Zar1l	11.01	60.65				
Zbp1		17.13				
Zc2hc1b		25.24				
Zc3hav1		2.88				
Zcrb1				2.07		
Zfas1		6.77		2.70	3.75	
Zfp365		3.90				
Zfp385a	3.26	5.56				
Zfp385b		5.17				
Zfp773	5.45					
Zfp804b	3.23					
Zfpm2		2.20				

Zglp1		23.35
Zmat3		3.08
Zmym1	2.37	
Zwilch	5.33	
Zwint		2.28

Table S8: Primer Sequence.

Gene	Direction	Sequence
Prx1-Cre	Forward	5'-CAGGCGCCGCACAAAGGC-3'
	Reverse	5'-TGAACGAACCTGGTCGAAATC-3'
Tfam	Forward	5'-CTGCCTTCCTCTAGCCCGGG-3'
	Reverse	5'-GTAACAGCAGACAACTTGTG-3'
Hif1dPA	Forward Mutant	5'-CCGGTAGAATTCCTGCAGGTCGAGGG-3'
	Reverse WT	5'-GGAGCGGGAGAAATGGATATG-3'
	Forward WT	5'-CGTGATCTGCAACTCCAGTC-3'
Ai14 Tomato	Forward WT	5'-CTCTGCTGCCTCCTGGCTTCT-3'
	Reverse WT	5'-CGAGGCGGATCACAAGCAATA-3'
	Reverse Mutant	5'-TCAATGGGCGGGGGTCGTT-3'
β_2-Microglobulin	Forward	5'-TCATTAGGGAGGAGCCAATG-3'
	Reverse	5'-ATCCCCTTTCGTTTTTGCTT-3'
mt-CytB	Forward	5'-TGAGGGGGCTTCTCAGTAGA-3'
	Reverse	5'-TAGGGCCGCGATAATAAATG-3'
mt-Cox3	Forward	5'-TAACCCTTGGCCTACTCACC-3'
	Reverse	5'-TAGGGCCGCGATAATAAATG-3'
mt-16SrRNA	Forward	5'-GGGATAACAGCGCAATCCTA-3'
	Reverse	5'-ATTGGGATGTCCTGATCCAA-3'

SUPPLEMENTAL FIGURES

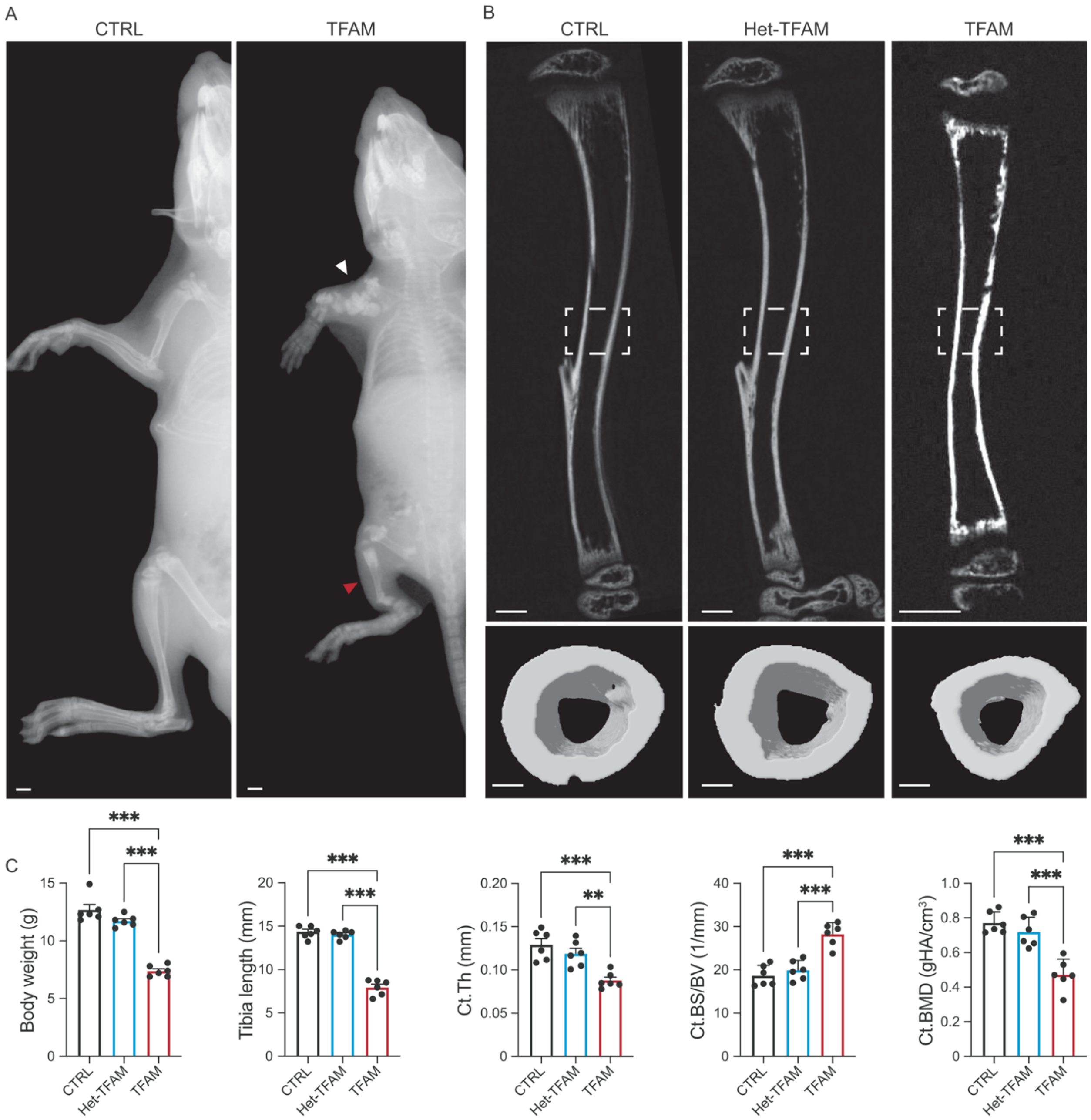

Supplemental Figure 1. Loss of TFAM in PRX lineage cells results in low cortical bone mass and spontaneous fractures in male mice.

(A) X-ray images of postnatal day 21 (p21) *TFAM*$^{fl/fl}$ (CTRL) and *PRX;TFAM*$^{fl/fl}$ (TFAM) mutant male mice. Arrows indicate multiple midshaft fractures in the forelimb (white arrow) and hindlimb (red arrow) of mutant mice. Scale bars: 1 mm. (B) Longitudinal (top) and mid-shaft transverse (bottom) micro-CT scans of tibias isolated from CTRL, *PRX;TFAM*$^{fl/+}$ (Het-TFAM), and TFAM p21 male mice. Scale bars: 500 µm for the longitudinal section and 100 µm for the transverse section. (C) Analysis of morphometric and micro-CT parameters, including body weight, tibia length, cortical thickness (Ct.Th), ratio of cortical bone surface to bone volume (Ct.BS/BV), and cortical bone mineral density (Ct.BMD). Evaluations were conducted on mutant mice and their respective

control littermates, with a minimum of five mice per group. Statistical analysis employed one-way ANOVA complemented with Bonferroni's post-hoc test for multiple comparisons. Data are presented as mean ± SD. Significance levels are denoted as $^{*}P \leq 0.05$, $^{**}P \leq 0.01$, $^{***}P \leq 0.001$.

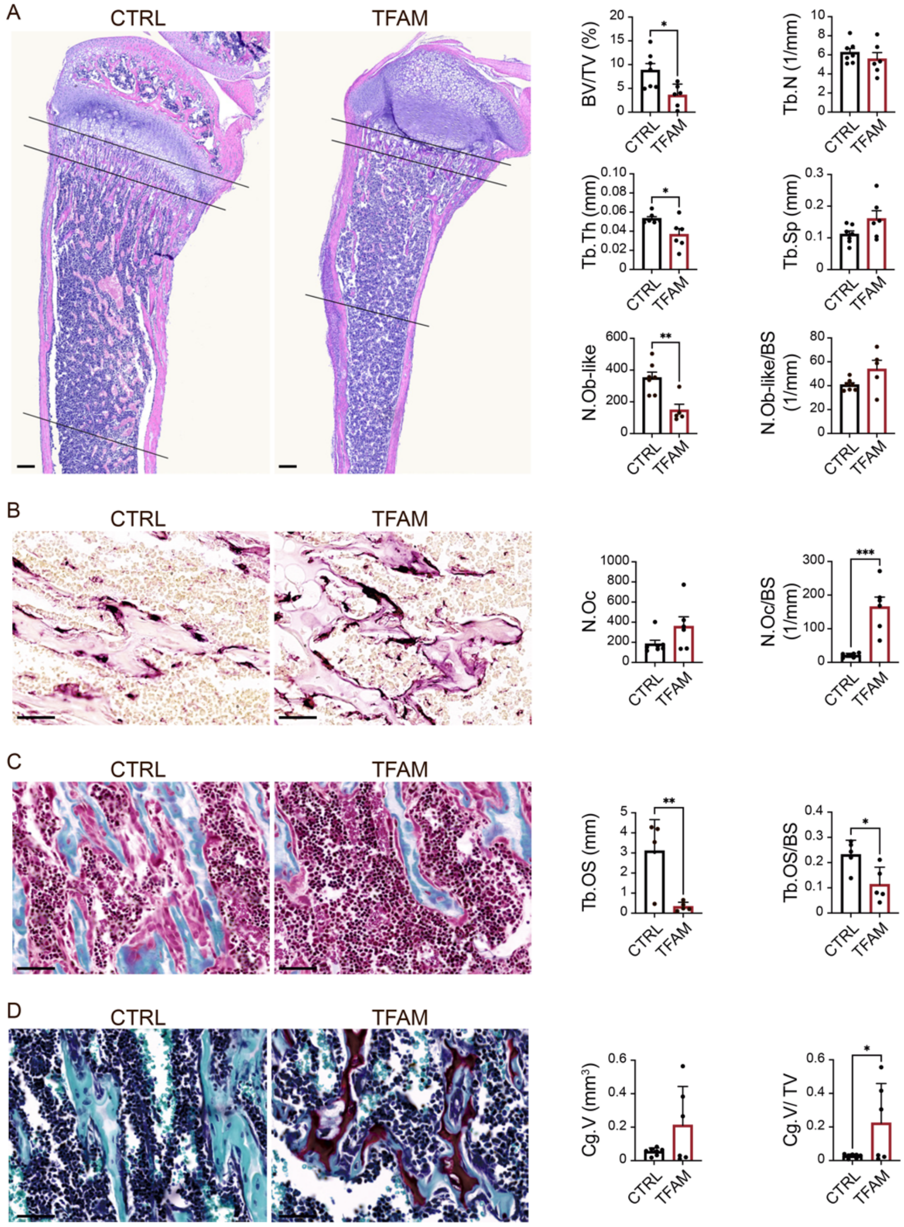

Supplemental Figure 2. Loss of TFAM in PRX lineage cells results in a low trabecular bone mass phenotype.

(A) On the left, H&E staining of longitudinal paraffin sections of p21 tibias. On the right, static histomorphometry analysis of trabecular bone. Quantifications of bone volume to tissue volume ratio (BV/TV), trabecular number (Tb.N), trabecular thickness (Tb.Th), trabecular separation (Tb.Sp), number of osteoblast-like cells (N.Ob-like), and number of Ob-like cells per bone surface (N.Ob-like/BS) are shown. Scale bar: 300 µm. (B) On the left, TRAP staining of longitudinal paraffin sections of p21 tibias with corresponding graphs quantifying osteoclast number (N.Oc) and osteoclasts per bone surface (N.Oc/BS) on the right. Scale bar: 100 µm. (C) On the left, Goldner's trichrome staining of longitudinal paraffin sections of p21 tibias with corresponding graphs quantifying osteoid volume (Tb.Os) and osteoid per bone surface (Tb.Os/BS) on the right. Scale bar: 100 µm. (D) On the left, Safranin-O staining of longitudinal paraffin sections of p21 tibias with corresponding graphs quantifying cartilage remnants volume (Cg.V) and cartilage

volume over trabecular volume (Cg.V/TV) on the right. Scale bar: 100 µm. Evaluations included mutant mice and their respective control littermates with a minimum of five mice per group. Data are presented as mean ± SD. Statistical analysis was performed using Student's t-test. Significance levels are indicated as *$P \leq 0.05$, **$P \leq 0.01$, ***$P \leq 0.001$.

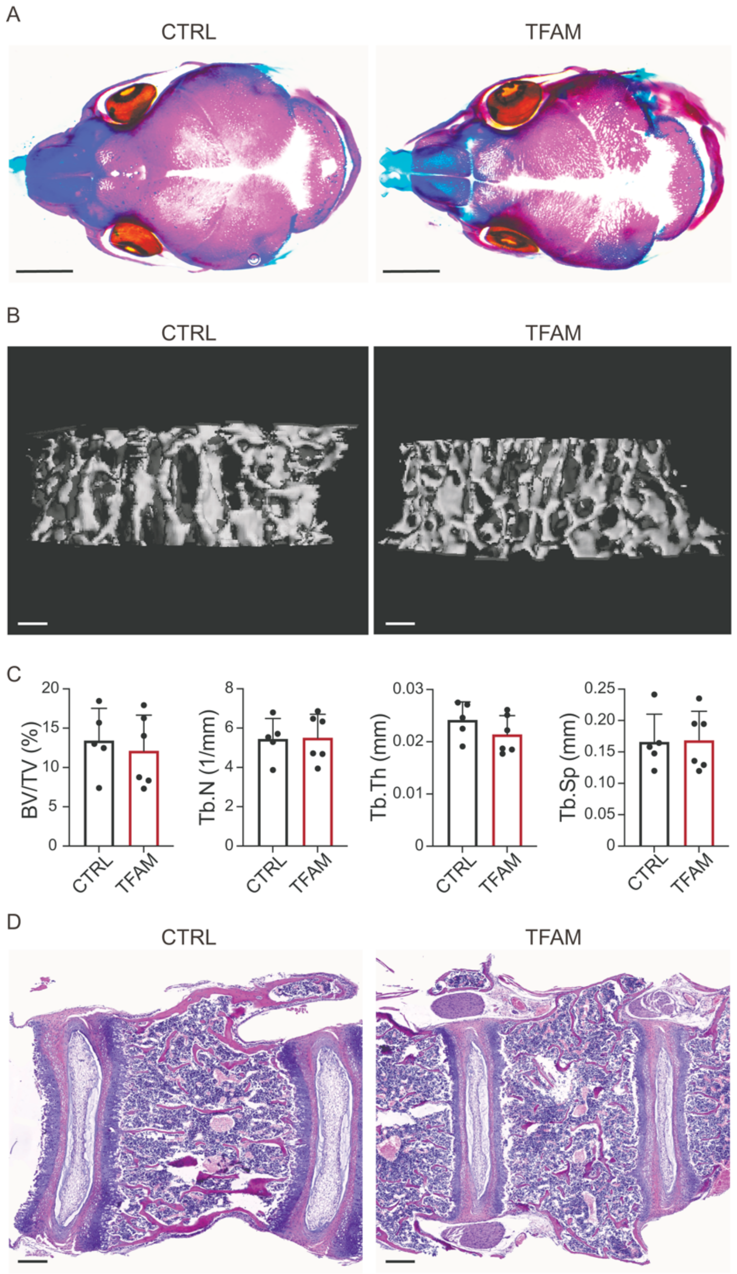

Supplemental Figure 3. The low bone mass phenotype observed in TFAM mutant limbs is unrelated to either altered growth plate development or systemic factors.
(A) Whole-mount Alizarin red and Alcian blue staining of postnatal day 0 (p0) skulls isolated from CTRL and TFAM mice. Scale bar: 2 mm. (B) Micro-CT scans of vertebrae (L5) isolated from CTRL and TFAM p21 male mice. Scale bar: 100 µm. (C) Histomorphometric analysis of CTRL and TFAM vertebrae at p21. Quantification of bone volume/tissue volume (BV/TV), trabecular number (Tb.N), trabecular thickness (Tb.Th), and trabecular separation (Tb.Sp) are shown. (D) H&E staining of CTRL and TFAM vertebrae at p21. Scale bar: 100 µm. Evaluations included mutant mice and their respective control littermates, with a minimum of five mice per group. Data are presented as mean ± SD. Statistical analysis was performed using Student's t-test. Significance threshold was set at $P \leq 0.05$.

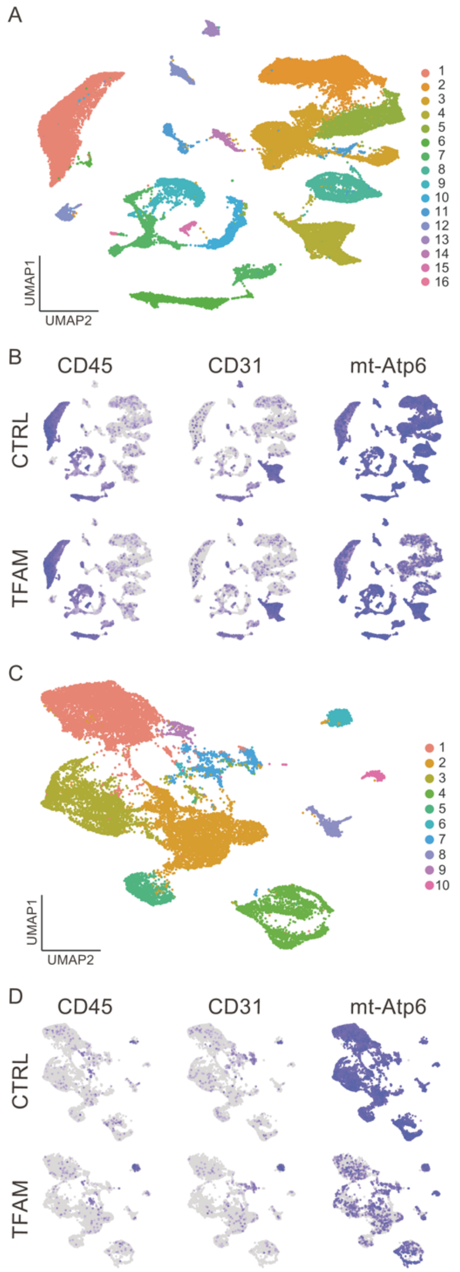

Supplemental Figure 4. Sequential optimization of periosteal cell subtype clusters.
(A) Uniform Manifold Approximation and Projection (UMAP) visualization of periosteal cells at resolution 0.1, displaying 16 original cell subtype clusters. (B) UMAP analysis comparing CTRL and TFAM clusters employing markers such as *CD45* and *CD31* mRNAs along with mitochondrial ATP synthase subunit 6 (*mt-Atp6*) to gate out hematopoietic ($CD45^+$) and endothelial ($CD31^+$) cell clusters and cells with high TFAM activity. (C) UMAP visualization following the removal of $CD31^+$ and $CD45^+$ cells and cells with high levels of expression of *mt-ATP6* mRNA indicating persistent TFAM activity. (D) Final UMAP assessment upon exclusion of both $CD45^+$ hematopoietic and $CD31^+$ endothelial cells and cells with high *mt-ATP6* mRNA expression. Biological and technical duplicates were used in the analysis.

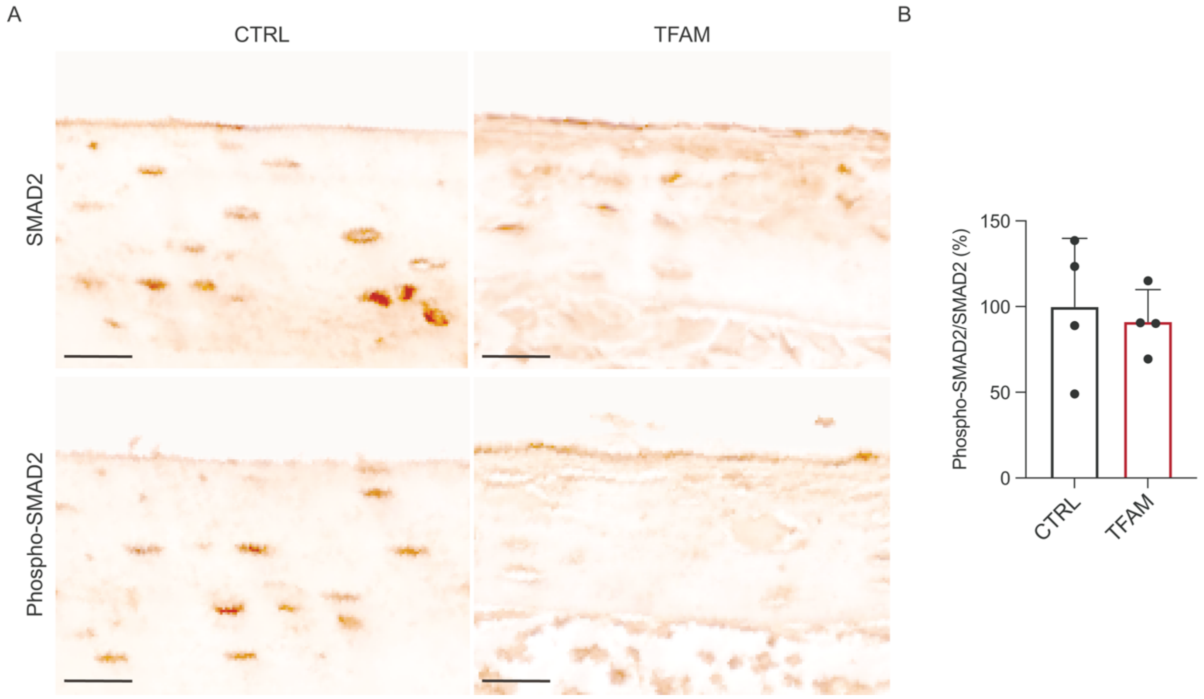

Supplemental Figure 5. Analysis of canonical TGFB signaling in periosteal cells.
(A) Immunohistochemistry (IHC) staining for SMAD2 (top) and phospho-SMAD2 (bottom) in longitudinal paraffin section of p21 tibias. Scale bar: 100 µm. (B) Quantification of the ratio of phospho-SMAD2 to SMAD2 in periosteal cells. Statistical significance was determined using unpaired t-test, based on data from 2 biological replicates. Data are presented as mean ± SD and normalized to control (CTRL) levels to account for experimental variability. Significance threshold was set at $P \leq 0.05$.

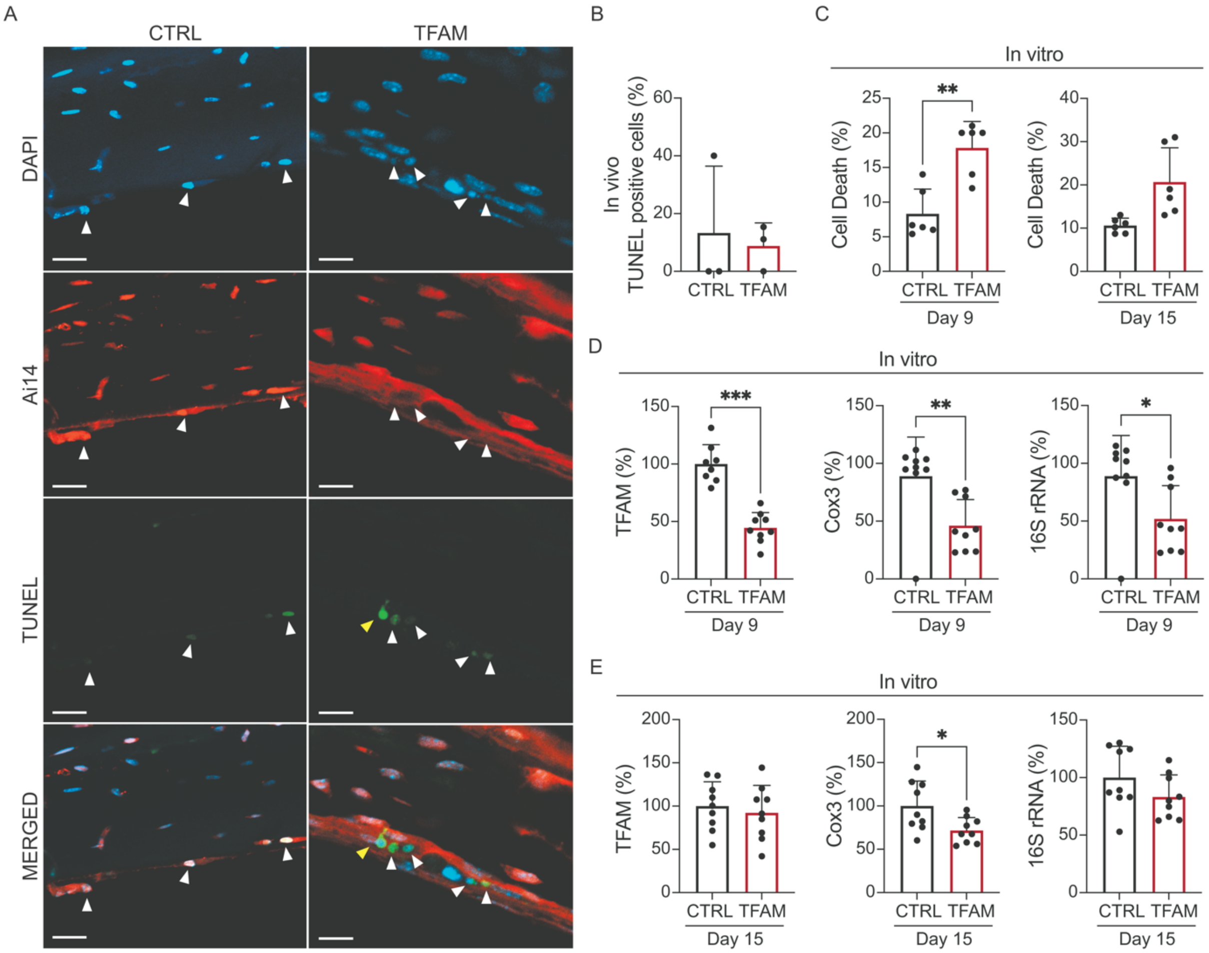

Supplemental Figure 6. Loss of TFAM does not affect cell survival in vivo.
(A) TUNEL staining of longitudinal fixed frozen sections of p21 tibias. Scale bar: 100 µm. White arrows indicate TUNEL-positive cells. The yellow arrow indicates an artifact. (B) Quantification of the percentage of TUNEL-positive cells over total number of Ai14-positive cells in vivo. (C) Percentage of cell death measured by trypan blue exclusion after 9 and 15 days of in vitro culture. (D) Efficiency of recombination of TFAM floxed allele at day 9. (E) Efficiency of recombination of TFAM floxed allele at day 15. Statistical significance was determined using unpaired t-test. Data are presented as mean ± SD and normalized to control (CTRL) levels. Significance threshold was set at P ≤ 0.05. Biological duplicates and technical duplicates or triplicates were used.

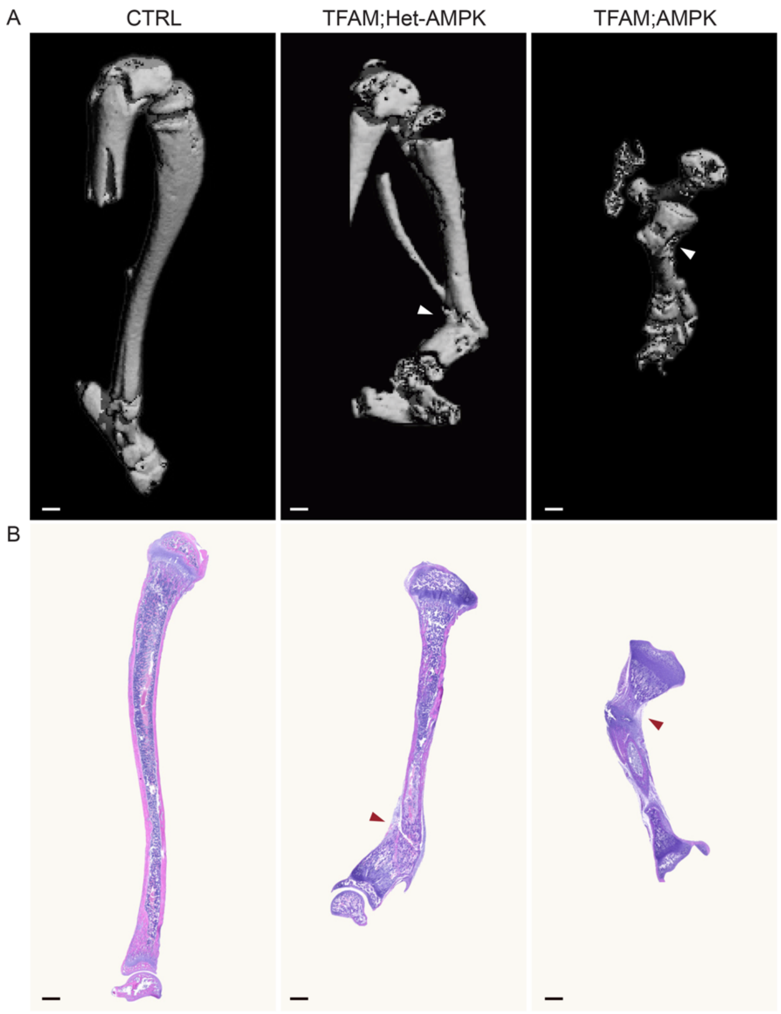

Supplemental Figure 7. AMPK deletion exacerbates the phenotype in TFAM-deficent mice. (A) Longitudinal micro-CT scans of tibias isolated from CTRL, *PRX;TFAM*$^{fl/fl}$*;AMPK*$^{fl/+}$ (TFAM;Het-AMPK), and *PRX;TFAM*$^{fl/fl}$*;AMPK*$^{fl/fl}$ (TFAM;AMPK) p21 female mice. Scale bars: 100 µm. (B) H&E staining of longitudinal paraffin sections of p21 tibias. Scale bars: 100 µm. The data presented are representative of three biological replicates. White and red arrows indicate the locations of fractures.

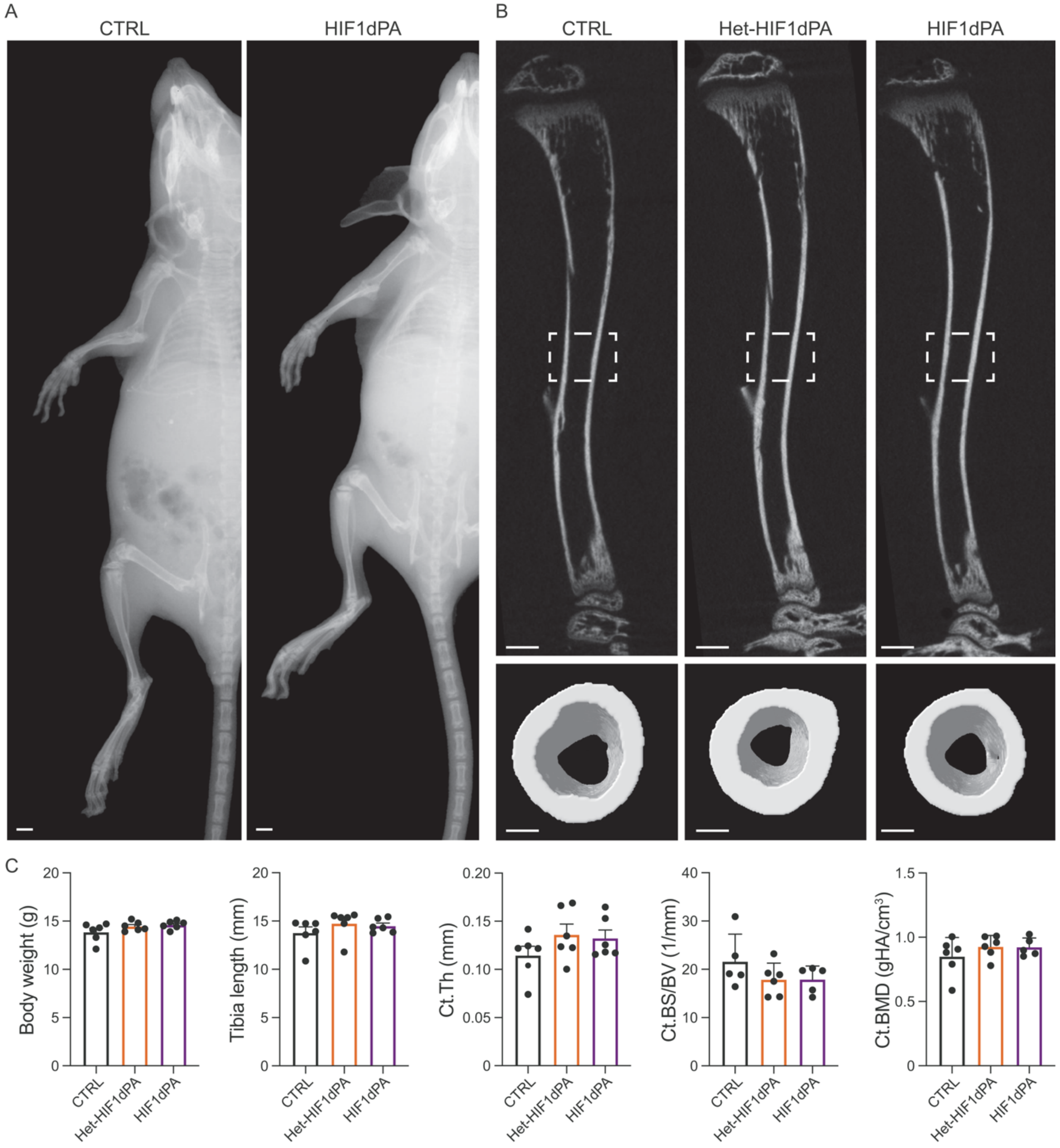

Supplemental Figure 8. HIF1dPA expression in PRX lineage cells does not affect cortical bone mass in long bones of female mice at p21.
(A) X-ray images of p21 *HIF1dPA$^{fl/fl}$* (CTRL) and *PRX;HIF1dPA$^{fl/fl}$* (HIF1dPA) mutant female mice. Scale bars: 1 mm. (B) Longitudinal (top) and mid-shaft transverse (bottom) micro-CT scans of tibias isolated from CTRL, *PRX;HIF1dPA$^{fl/+}$* (Het-HIF1dPA), and HIF1dPA p21 female mice. Scale bars: 500 µm for the longitudinal section and 100 µm for the transverse section. (C) Analysis of morphometric and micro-CT parameters, including body weight, tibia length, cortical thickness (Ct.Th), ratio of cortical bone surface to bone volume (Ct.BS/BV), and cortical bone mineral density (Ct.BMD). Evaluations were conducted on mutant mice and their respective control littermates, with a minimum of five female mice per group. Statistical analysis employed one-way ANOVA complemented with Bonferroni's post-hoc test for multiple comparisons. Data are presented as mean ± SD. Significance threshold was set at P ≤ 0.05.

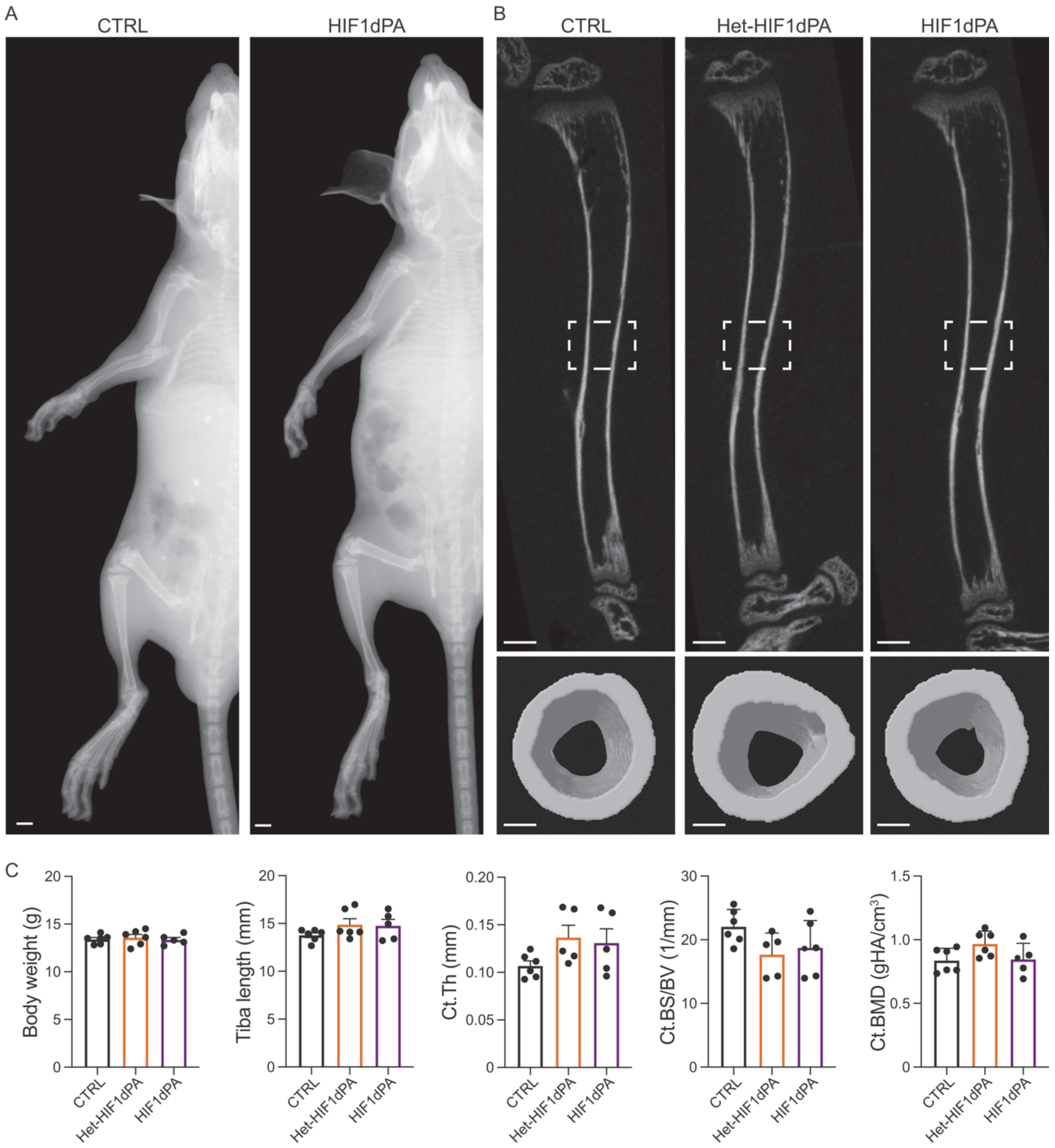

Supplemental Figure 9. HIF1dPA expression in PRX lineage cells does not affect cortical bone mass in long bones of male mice at p21.

(A) X-ray images of p21 *HIF1dPA*$^{fl/fl}$ (CTRL) and *PRX;HIF1dPA*$^{fl/fl}$ (HIF1dPA) mutant male mice. Scale bars: 1 mm. (B) Longitudinal (top) and mid-shaft transverse (bottom) micro-CT scans of tibias isolated from CTRL, *PRX;HIF1dPA*$^{fl/+}$ (Het-HIF1dPA), and HIF1dPA p21 male mice. Scale bars: 500 μm for the longitudinal section and 100 μm for the transverse section. (C) Analysis of morphometric and micro-CT parameters, including body weight, tibia length, cortical thickness (Ct.Th), ratio of cortical bone surface to bone volume (Ct.BS/BV), and cortical bone mineral density (Ct.BMD). Evaluations were conducted on mutant mice and their respective control littermates, with a minimum of five male mice per group. Statistical analysis employed one-way ANOVA complemented with Bonferroni's post-hoc test for multiple comparisons. Data are presented as mean ± SD. Significance threshold was set at $P \leq 0.05$.

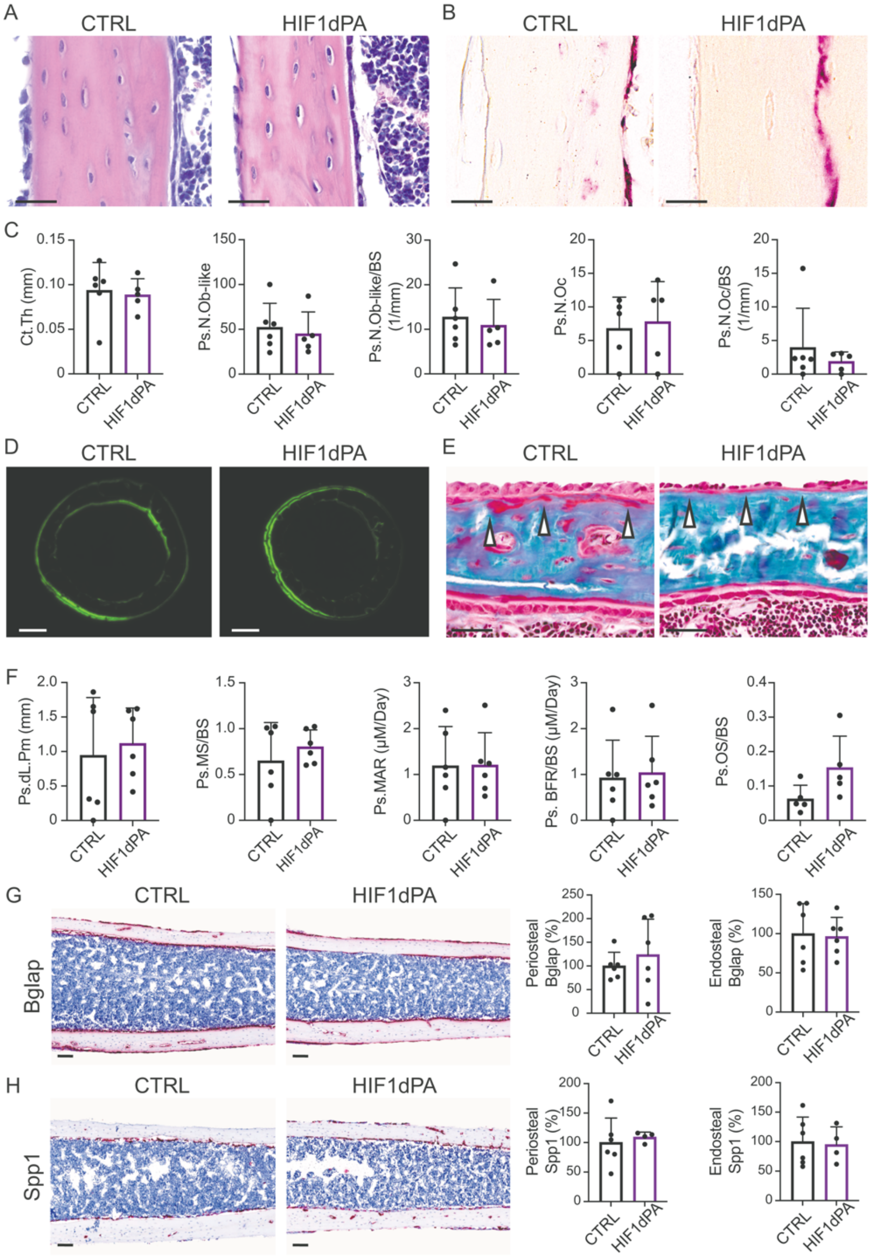

Supplemental Figure 10. HIF1dPA expression in PRX lineage cells does not induce significant alterations in either cellularity or osteoblastic activity in the periosteum of long bones of female mice at p21.

(A) H&E staining of longitudinal paraffin sections of p21 tibias, illustrating cortical bone at midshaft. Scale bar: 100 µm. (B) TRAP staining of longitudinal paraffin sections of p21 tibias. Scale bar: 100 µm. (C) Static histomorphometry analysis conducted on longitudinal paraffin sections of p21 tibias, quantifying cortical thickness (Ct.Th), number of periosteal osteoblast-like cells (Ps.N.Ob-like), osteoblast-like cells per bone surface (Ps.N.Ob-like/BS), number of periosteal osteoclasts (Ps.N.Oc), and osteoclast-to-bone surface ratio (Ps.N.Oc/BS). (D) Calcein labeling in transverse MMA sections of p21 tibias, showing cortical bone at midshaft. Scale bar: 200 µm. (E) Goldner's Trichrome staining of longitudinal MMA sections of p21 tibias, showing cortical bone at midshaft. White arrows indicate the presence of osteoid. Scale bar: 100 µm.

(F) Dynamic histomorphometry analysis performed on transverse MMA sections of p21 tibias, quantifying periosteal double-labeled surface (Ps.dL.PM), mineralizing surface over bone surface (Ps.MS/BS), mineral apposition rate (Ps.MAR), bone formation rate over bone surface (Ps.BFR/BS), and osteoid accumulation (Ps.OS/BS). (G, H) RNAscope analysis conducted on longitudinal paraffin sections of p21 tibias, showing cortical bone at midshaft. The levels of expression of *Bglap* (G) and *Spp1* (H) mRNAs were investigated, with quantification of the signal provided in both periosteum and endosteum. Scale bar: 100 µm. Evaluations included a minimum of five mice per group for both mutants and their respective control littermates. Data are expressed as mean ± SD. Statistical analysis utilized Student's t-test. Significance threshold was set at $P \leq 0.05$.

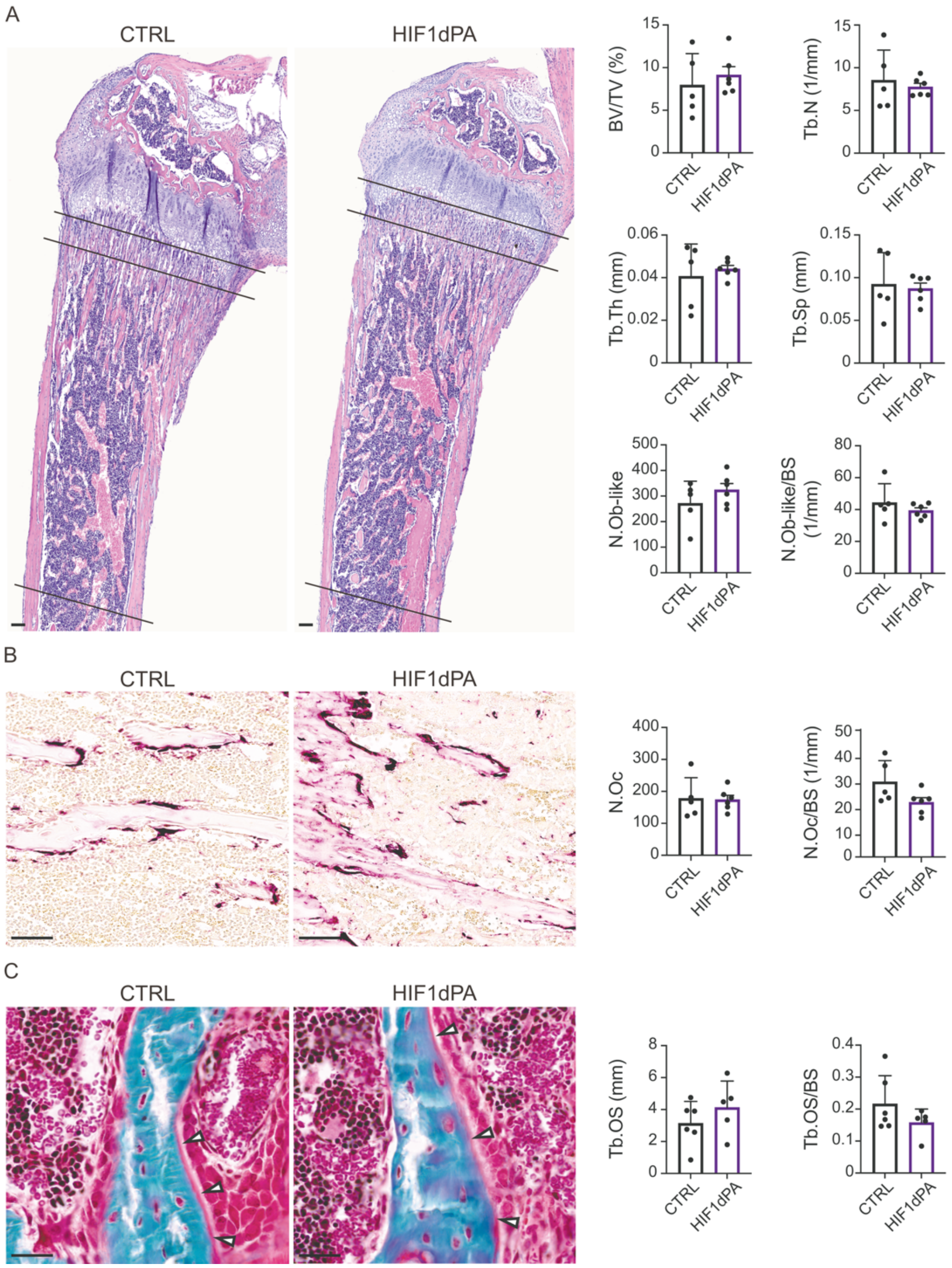

Supplemental Figure 11. HIF1dPA expression activity in PRX lineage cells does not affect trabecular bone in long bones of female mice at p21.

(A) On the left, H&E staining of longitudinal paraffin sections of p21 tibias. On the right, static histomorphometry analysis of trabecular bone. Quantifications of bone volume to tissue volume ratio (BV/TV), trabecular number (Tb.N), trabecular thickness (Tb.Th), trabecular separation (Tb.Sp), number of osteoblast-like cells (N.Ob-like), and number of Ob-like cells per bone surface (N.Ob-like/BS) are shown. Scale bar: 300 μm. (B) On the left, TRAP staining of longitudinal paraffin sections of p21 tibias with corresponding graphs quantifying osteoclast number (N.Oc) and osteoclasts per bone surface (N.Oc/BS) on the right. Scale bar: 100 μm. (C) On the left, Goldner's Trichrome staining of longitudinal paraffin sections of p21 tibias with corresponding graphs quantifying osteoid volume (Tb.Os) and osteoid per bone surface (Tb.Os/BS) on the right. Scale bar: 100 μm. Evaluations included mutant mice and their respective control littermates with

a minimum of five female mice per group. Data are presented as mean ± SD. Statistical analysis was performed using Student's t-test. Significance threshold was set at $P \leq 0.05$.

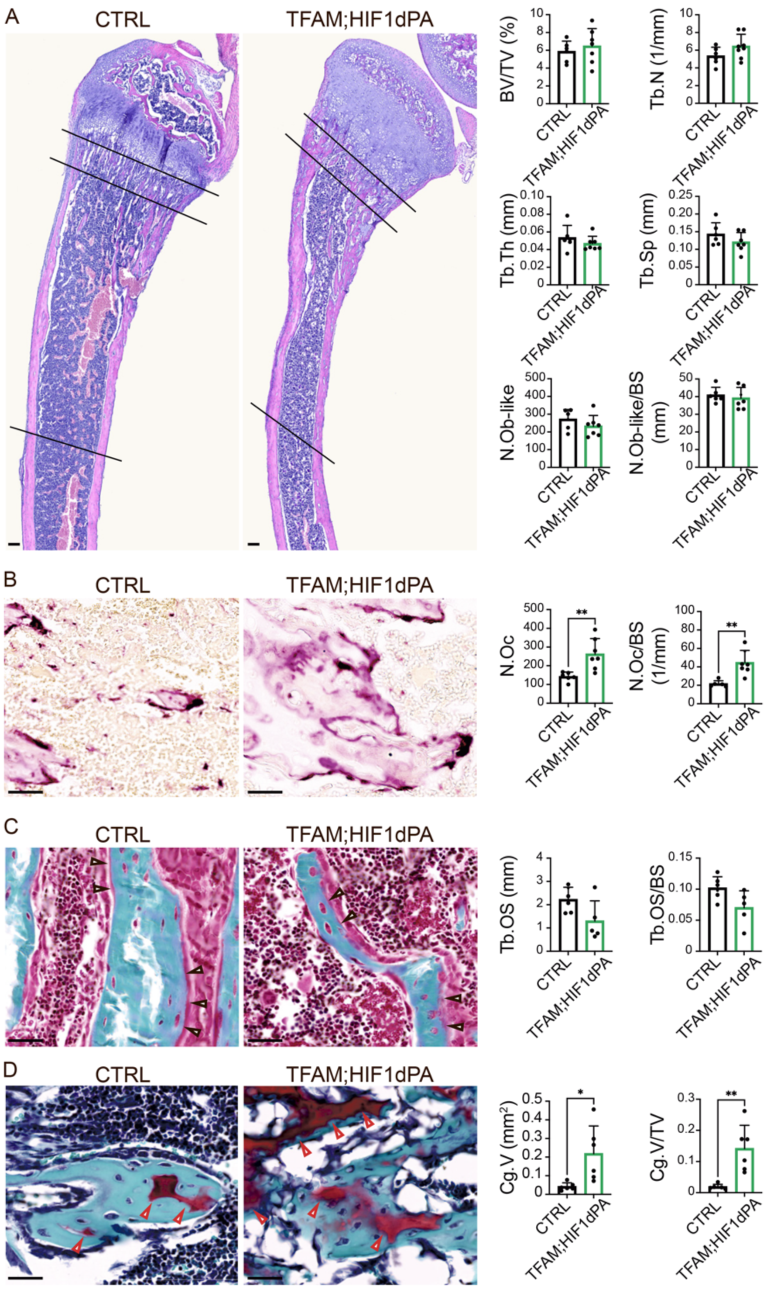

Supplemental Figure 12. HIF1dPA corrects the low trabecular bone mass phenotype observed in TFAM mice.

(A) On the left, H&E staining of longitudinal paraffin sections of p21 tibias. On the right, static histomorphometry analysis of trabecular bone. Quantifications of bone volume to tissue volume ratio (BV/TV), trabecular number (Tb.N), trabecular thickness (Tb.Th), trabecular separation (Tb.Sp), number of osteoblast-like cells (N.Ob-like), and number of Ob-like cells per bone surface (N.Ob-like/BS) are shown. Scale bar: 300 μm. (B) On the left, TRAP staining of longitudinal paraffin sections of p21 tibias with corresponding graphs quantifying osteoclast number (N.Oc) and osteoclasts per bone surface (N.Oc/BS) on the right. Scale bar: 100 μm. (C) On the left, Goldner's Trichrome staining of longitudinal paraffin sections of p21 tibias with corresponding graphs quantifying osteoid volume (Tb.Os) and osteoid per bone surface (Tb.Os/BS) on the right.

Scale bar: 100 µm. (D) On the left, Safranin-O staining of longitudinal paraffin sections of p21 tibias with corresponding graphs quantifying cartilage remnants volume (Cg.V) and cartilage volume over trabecular volume (Cg.V/TV) on the right. Scale bar: 100 µm. Evaluations included mutant mice and their respective control littermates with a minimum of five female mice per group. Data are presented as mean ± SD. Statistical analysis was performed using Student's t-test. Significance levels are indicated as $^{*}P \leq 0.05$, $^{**}P \leq 0.01$.

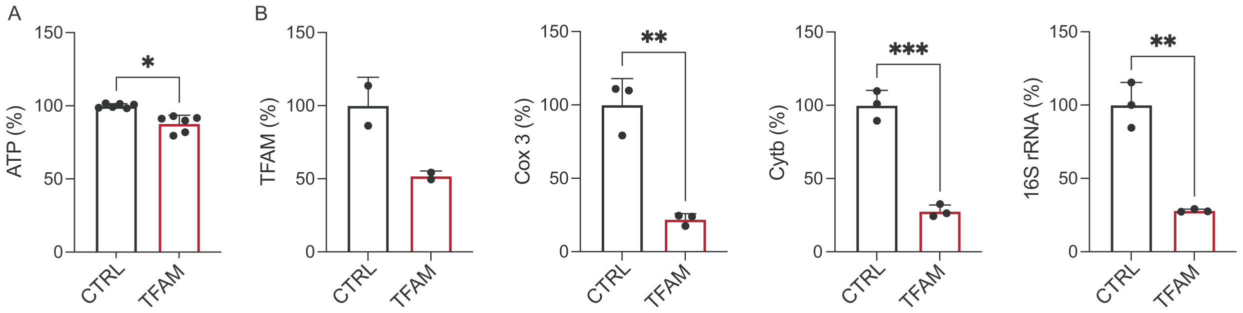

Supplemental Figure 13. Loss of TFAM reduces steady-state intracellular ATP levels in unsorted periosteal cells.

(A) Quantitative analysis of ATP concentrations in cultured periosteal cells comparing TFAM$^{fl/fl}$ (CTRL), PRX;TFAM$^{fl/fl}$ (TFAM) periosteal cells. (B) Quantification of TFAM floxed allele recombination efficiency using 2loxP qPCR in TFAM mutant periosteal cells relative to CTRL, alongside qPCR quantification of mitochondrial gene expression, focusing on Cytochrome c oxidase 3 (*Cox3*), Cytochrome B (*Cytb*), and 16S ribosomal RNA (*16S rRNA*) genes in CTRL and TFAM periosteal cells. Experiments employed biological and technical duplicates or triplicates. Statistical significance was determined using a Wilcoxon matched-pairs signed-rank test or an unpaired t-test as appropriate, based on the data distribution and variance characteristics. Data are expressed as mean ± SD, with normalization to CTRL levels to account for experimental variability. Significance thresholds are indicated as follows: *$P \leq 0.05$, **$P \leq 0.01$, ***$P \leq 0.001$.
